# epialleleR: an R/Bioconductor package for sensitive allele-specific methylation analysis in NGS data

**DOI:** 10.1093/gigascience/giad087

**Published:** 2023-10-31

**Authors:** Oleksii Nikolaienko, Per Eystein Lønning, Stian Knappskog

**Affiliations:** K. G. Jebsen Center for Genome-Directed Cancer Therapy, Department of Clinical Science, University of Bergen, Bergen 5021, Norway; K. G. Jebsen Center for Genome-Directed Cancer Therapy, Department of Clinical Science, University of Bergen, Bergen 5021, Norway; Department of Oncology, Haukeland University Hospital, Bergen 5021, Norway; K. G. Jebsen Center for Genome-Directed Cancer Therapy, Department of Clinical Science, University of Bergen, Bergen 5021, Norway; Department of Oncology, Haukeland University Hospital, Bergen 5021, Norway

**Keywords:** epigenetics, DNA methylation, somatic mosaicism, epigenetic mosaicism, methylation sequencing

## Abstract

Low-level mosaic epimutations within the *BRCA1* gene promoter occur in 5–8% of healthy individuals and are associated with a significantly elevated risk of breast and ovarian cancer. Similar events may also affect other tumor suppressor genes, potentially being a significant contributor to cancer burden. While this opens a new area for translational research, detection of low-level mosaic epigenetic events requires highly sensitive and robust methodology for methylation analysis. We here present epialleleR, a computational framework for sensitive detection, quantification, and visualization of mosaic epimutations in methylation sequencing data. Analyzing simulated and real data sets, we provide in-depth assessments of epialleleR performance and show that linkage to epihaplotype data is necessary to detect low-level methylation events. The epialleleR is freely available at https://github.com/BBCG/epialleleR and https://bioconductor.org/packages/epialleleR/ as an open-source R/Bioconductor package.

## Introduction

Cancer is a major health threat and cause of death worldwide. While the minority of cases are due to highly penetrant germline pathogenic variants (inherited cancers), the majority are considered sporadic cancers with no known germline genetic component.

In addition to genetic aberrations like single-nucleotide variants, indels, copy number alterations, and rearrangements, cancers are known to harbor epimutations [1, 2] (i.e., epigenetic disturbances) that lead to aberrant transcriptional up- and downregulation. Such aberrations are often studied at the level of cytosine DNA methylation. As typical promoters of active genes are hypomethylated, epimutations within such regions are manifested as DNA hypermethylation—the common mechanism of gene repression in cancer [3]. For example, aberrant DNA hypermethylation events (epimutations) within promoters of tumor suppressor genes *BRCA1, MGMT*, and *MLH1* were shown to be associated with downregulation of expression of these genes [4–6], and the presence of such epimutations further guides treatment strategies in clinical practice [7–9].

Epigenetic aberrations may arise during different stages of carcinogenesis as somatic epimutations (mirroring somatic mutations) or *in utero* (affecting several germline layers) as constitutional normal tissue epimutations. Several studies in large cohorts [10, 11] have linked constitutional (prenatal), mosaic (affecting a small subset of cells only) epimutations to breast and/or ovarian cancer risk. Research and interest in this field, however, have been limited by the fact that all these studies were conducted on patients already diagnosed with their cancers, questioning whether normal tissue methylation in these patients may be a cancer-initiating event or a secondary effect of the disease itself. Recently, we found frequent (occurring in >5% of healthy women) though low-level (down to 0.03% of affected alleles) mosaic epimutations within the *BRCA1* gene promoter to be associated with a significantly elevated risk for subsequent high-grade ovarian as well as triple-negative breast cancer, in a large, population-based prospective cohort [12]. This finding raises a provoking question of whether similar low-level mosaic epimutations may affect other tumor suppressor genes and be associated with an elevated risk of other cancer forms as well. While this opens a new research area related to cancer risk, there are technical issues to account for, as the low frequency of such mosaic epimutations limits the amplitude of observed changes in methylation. Thus, to explore such hypotheses, there is a need for robust and sensitive epimutation detection techniques.

Currently, the most widely used methods for DNA methylation profiling are BeadChip arrays (such as Illumina HumanMethylation450 or HumanMethylationEPIC) and a variety of methylation sequencing techniques (for details see [13]). These methods have different pros and cons: arrays allow genome-wide assessment at a reduced cost, while the sequencing provides additional information on haplotype specificity of DNA methylation. The typical bioinformatic workflows designed to analyze both types of data usually result in sets of beta values (ratio of a count of methylated cytosines to the total sum of methylated and unmethylated bases) for each genomic position covered [14–16]. While this approach is suitable for addressing large differences in DNA methylation profiles between 2 sets of samples (e.g., cases and controls), it lacks sensitivity for low-level mosaic epimutation detection, as the detection is hindered by sometimes much more common biological variation [17, 18] or technical artifacts [19, 20]. Moreover, the lack of haplotype linkage makes such analysis difficult in BeadChip array-based datasets and therefore requires nontrivial approaches [21]. Gene promoter methylation present in a low fraction of molecules may be detected by conventional methylation-specific quantitative polymerase chain reaction (MS-qPCR), but the discrimination between methylated and unmethylated alleles is limited to the CpGs directly covered by the primers/probes [10]. In contrast to other methods, analysis of next-generation sequencing (NGS)-based data can provide much higher sensitivity when the base resolution methylation data are combined with information on allelic belongingness (epihaplotype linkage).

Here, we present a computational framework for sensitive detection and quantification of low-frequency, mosaic epimutations in methylation sequencing data. The provided methods can be used for the discovery of low-frequency epialleles (mitotically and/or meiotically heritable DNA methylation patterns [22]) connected to disease risk (as done previously in [12, 23]), as well as for purposes allowing less sensitivity, such as assessments related to treatment response [24, 25], or to the development of treatment resistance [26]. Importantly, the framework also allows one to connect DNA methylation status with potential underlying *cis*-factors, such as single-nucleotide variations or mutations within the immediate proximity.

The versatility of the framework makes it applicable for analysis of data from any methylation sequencing experiment, given that methylation in these data can be called at individual cytosine residues. Both single-end and paired-end sequencing alignment files can be used as an input, and in cases where methylation calls are not available, this framework allows one to call cytosine methylation and permanently store calls in a binary sequence alignment/map (BAM) file.

Similar to other tools that transform NGS reads into counts of bases or molecules, the framework is not designed to determine preanalytical bias, such as cell-type heterogeneity. Appropriate methods must be used to control confounders in the downstream analyses [27, 28].

## Results

### epialleleR implementation

The presence of hypermethylated *BRCA1* alleles (epimutations) in normal tissue (white blood cells [WBCs]) has been shown *qualitatively* for 5–8% of adult women [10]. However, the associated *quantitative* changes in DNA methylation at the level of individual CpGs are typically small (in most cases, the intraindividual frequency of epimutations is between 0.03% and 1% [12]) and therefore indistinguishable from the background methylation level due to inherent biological (potentially spurious single-base methylation events) and technical (sequencing errors) variance [17]. Methylation statuses of neighboring CpGs are often concordant [29], and such spatially extended epigenetic changes are often associated with a gene expression silencing [30]. Given the potential biological (gene inactivation) and clinical (cancer risk) importance of epimutations, we focused on quantification of hypermethylation events that span over several CpGs, accounting for both methylation status of individual CpGs within the sequence read as well as the average methylation level of the sequence read itself. This is possible in NGS-based data sets, while it is not in array-based data where methylation information of different CpGs cannot be connected to each other as in haplotype data.

**Figure 1:**
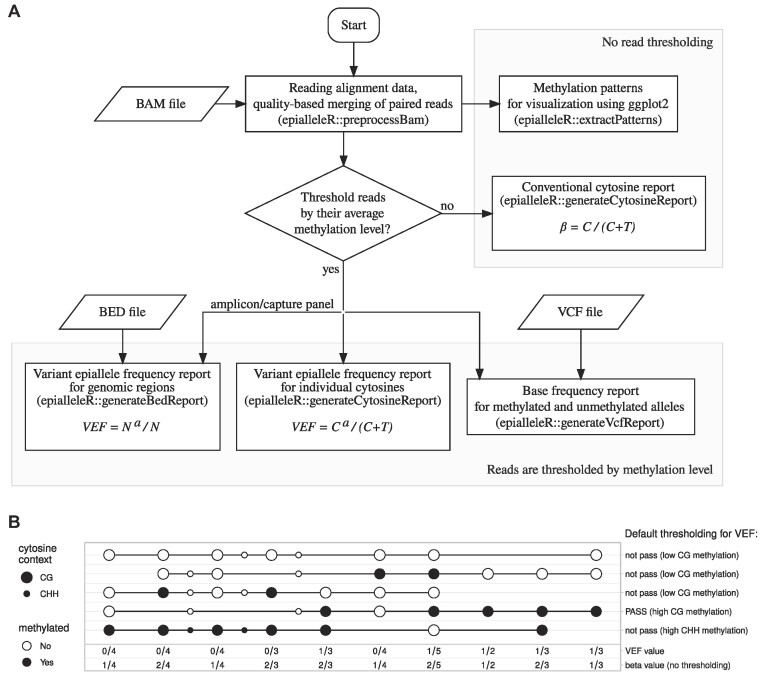
(A) Flowchart of epialleleR package data-processing steps. The formulas using to calculate conventional beta as well as VEF values are given in boxes. *C* and *T*, total number of cytosines and thymines at particular genomic position, respectively; *C^a^*, number of cytosines at particular genomic position within read pairs passing a particular methylation threshold (*C^a^* ≤ *C*); *N*, total number of read pairs, mapped to a particular genomic region; *N^a^*, number of mapped read pairs, passing a particular methylation threshold (*N^a^* ≤ *N*). (B) Schematic illustration of cytosine methylation (circles) within epialleles (horizontal lines) and results of thresholding by average read methylation level (labels on the right) using default parameters (i.e., at least 2 CpGs in CG context, at least 50% methylation within CG context, at most 10% methylation outside of CG context). These default thresholding parameters were chosen to detect hypermethylated alleles with biological relevance in tumor suppressor genes; detection of epimutations of a different nature may require adjustments to the default parameter values. Resulting per-cytosine beta and VEF values are given under each CpG (large circles). In the context of a typical CpG-rich regulatory region of an actively transcribed gene, the 3 hypomethylated epialleles on the top represent typically abundant scattered methylation or sequencing artifacts (only a minority of cytosines in CG context are called as methylated), the epiallele at the bottom represents the product of incomplete bisulfite conversion (cytosines in GG and non-CG contexts are methylated), and the second epiallele from the bottom represents a true biologically relevant epimutation (hypermethylation) that leads to gene silencing (majority of cytosines in CG context are methylated, while no methylation is detected in the non-CG context).

As number of events that lead to variance in methylation (base deamination, random single-base methylation events, and sequencing errors) is limited at the level of individual reads (only a fraction of CpGs might be affected within the same read), the average methylation level of the read will be moderately affected by such events and can help distinguish hyper- from hypomethylated epialleles (where methylation statuses of the majority of CpGs are concordant and average methylation level is either close to 0% or 100%). We therefore hypothesized that thresholding sequence reads by their average methylation level will reduce the effect of biological and technical variance and facilitate the detection of infrequent hypermethylation events. As no suitable generic solution was publicly available, we implemented it using R software environment for statistical computing [31], a *de facto* standard for scientific data analysis. The implemented solution, epialleleR, loads methylation call strings and short sequence reads from the supplied BAM file, optionally thresholds read pairs according to their methylation properties, and produces methylation reports for individual cytosines as well as genomic regions of interest (Fig. 1A). During BAM loading, pairs of sequence reads and corresponding methylation call strings are merged according to Phred quality score values (i.e., base with the highest score is chosen) to preserve information of the highest quality. In contrast to approaches that involve simple trimming of overlapping parts of read2, the following approach might retain more information when higher-quality fragments of read2 (5′-end or middle) overlap with lower-quality fragments of read1 (3′-end). The optional thresholding defines a subpopulation of epialleles of interest and is based on the minimum number and the average methylation level of cytosines in various sequence contexts (e.g., CG, CHG, or CHH). The thresholding parameters are fully adjustable to target desired population of epialleles; their default values (minimum 2 CpG sites, minimum average methylation beta value of 0.5 for CpG sites, maximum average methylation beta value of 0.1 for non-CpG sites) performed well in the study linking mosaic *BRCA1* epimutations and cancer risk [12] and were used here in all downstream analyses.

The optional thresholding of sequence reads defines 2 modes of epialleleR (v.1.3.5, RRID:SCR_023913 [32]) function. Without thresholding, epialleleR produces conventional cytosine reports similar to the ones produced by other tools (e.g., Bismark [14]). In this case, methylation beta value for every genomic location is computed as a ratio of a number of methylated cytosines to the total number of methylated and unmethylated cytosines: *β = C*/(*C + T*).

When read thresholding is performed (default mode of action), the level of methylation per every genomic position, denoted as a variant epiallele frequency (VEF), is calculated as a ratio of a number of methylated cytosines in read pairs passing the threshold (*C^a^*) to total number of methylated and unmethylated cytosines in all read pairs: *VEF = C^a^*/(*C + T*) (see Fig. 1B for an example). When the report is prepared at a level of extended genomic regions rather than individual bases, VEF equals the ratio of a number of read pairs passing threshold (*N^a^*) to the total number of read pairs (*N*) overlapping the region of interest: *VEF = N^a^/N*. The term “variant epiallele” here represents a group of epialleles (i.e., individual methylation patterns) with similar methylation properties that is defined by thresholding; therefore, VEF effectively represents the frequency of this group of epialleles passing the threshold at the level of individual cytosines or extended genomic regions.

Methylation beta values (from conventional reporting) as well as VEF values (from default reporting mode with read thresholding) can be produced from any number of BAM files with no prior hypothesis, as long as experimental setup allows to call methylation on a per-base level. Both of these values effectively represent methylation levels per genomic position and, as such, can be directly used further as an input for other bioinformatic tools including, but not limited to, differential methylation analysis tools.

If methylation statuses of cytosines were not determined, epialleleR allows to create and store methylation calls, allowing analysis of BAM files created by various methylation sequencing alignment tools.

When optional data on single-nucleotide variants are provided (as a variant call format, VCF file, or a VCF object), epialleleR quantifies the balance or skewness of methylation between alleles, thereby enabling assessment of potential allele specificity of epimutations. In particular, this information is important for distinguishing epimutations that occurred through a single event followed by clonal expansion (e.g., prenatal epimutations that are present on the same allele in all affected cells, as in [12, 23]) from the ones that occurred in different cells independently and therefore present on both alleles. In some cases, allele specificity also allows to infer causality of epimutations in cancer development [23].

To provide a comprehensive range of means for epiallele analysis, the package also offers methods allowing visualization and characterization of *all* individual epialleles (methylation patterns) in a sample (see Fig. 1 and Supplementary figures for details). If required, extracted patterns can include other, noncytosine bases of interest (e.g., single-nucleotide variations), which allows to connect methylation properties of epialleles with sequence features in proximity. During methylation pattern extraction, every epiallele is characterized by number of context sites and methylation level (average beta value) and is assigned with a unique identifier (Fowler-Noll-Vo FNV-1a non-cryptographic hash [33]) that solely depends on positions of included cytosine (and other optional) bases and their methylation states (or nucleotide symbols for optional bases), enabling to not only group epialleles by their methylation properties but also reliably and consistently track individual epialleles of high importance across different samples or even studies. The average beta values for all extracted patterns as well as patterns themselves can be explored to optimize thresholding parameters for a genomic region of interest.

Increasing scale and depth of methylation sequencing experiments impose a requirement on the speed of data processing. Therefore, all time-consuming subtasks were implemented using optimized C/C++ subroutines and, whenever possible, linked to HTSlib, the unified C library for high-throughput sequencing data processing [34]. The R package epialleleR is freely available at the Bioconductor package repository [32].

### Reporting accuracy analyses

First, we sought to validate the accuracy of methylation reporting by epialleleR in its conventional mode (no read thresholding) as compared with 3 other commonly used tools for which read thresholding is not available: Bismark [14], methylKit [35], and Illumina DRAGEN Bio-IT Platform. For this purpose, we simulated large sets of paired-end bisulfite sequencing reads (2 × 151bp, 100 million read pairs covering human chromosome 19). In contrast to real datasets, simulated data allow to calculate “ground-truth” methylation levels for unbiased comparison. Simulation parameters were selected to obtain exact methylation levels of 50% for cytosines in the CG context (*n* = 2,211,240) and methylation level of approximately 0.25% (bisulfite conversion rate of ∼99.75%) for cytosines in the CHG and CHH contexts (*n* = 6,593,900 and 19,210,572, respectively). In addition to endogenous deamination events [17], bisulfite treatment-induced changes [19], and variation in conversion rates [36], sequencing itself can introduce errors that vary in range depending on assay type and sequencing technology [20]. Therefore, we introduced variable level of artificial sequencing errors (0%, 0.1%, 0.3%, or 0.6%) and evaluated their effect on the accuracy of reported methylation metrics, applying a selected set of methods (for comparison see Table 1). Analysis on exactly the same task (BAM file to cytosine report) revealed that reported values were close to their theoretical expectations for all methods, with epialleleR being the least affected by sequencing errors, that is, maintaining the smallest deviance of reported versus expected methylation beta values for all samples with sequencing errors introduced, possibly owing to read quality–assisted merging of paired reads (Table 2, further details in Supplementary Table S1).

**Table 1:** Selected characteristics of software/hardware solutions for cytosine methylation reporting

Method	Requires reference (genomic) sequence	Removes overlaps within read pairs	Outputs epiallele frequencies	Processing speed, read pairs per second
Bismark	yes (genome-wide cytosine reports) / no (bedGraph reports)	yes (trims read2)	no	40–2,800
methylKit	no	yes (trims read2)	no	9,900–15,400
DRAGEN	yes	yes (trims read2)	no	2,000–183,000
epialleleR	no	yes (base with the highest quality is chosen)	yes	129,000–231,000

**Table 2:** Selected accuracy metrics (average beta values and their variance) of cytosine methylation reporting. Average reported beta values that are closest to the expected beta values (0.0025 for cytosines in the CHG/CHH contexts and 0.5 for cytosines inthe CG context) and lowest variance values are shown in bold.

		CHH		CHG		CG	
Sequencing error rate	Method	Mean	Variance	Mean	Variance	Mean	Variance
0.00%	DRAGEN	**0.002501**	1.28E-05	**0.002500**	1.27E-05	0.499997	5.96E-07
	Bismark	0.002501	1.34E-05	0.002500	1.33E-05	**0.499997**	**5.92E-07**
	methylKit	0.002503	**1.28E-05**	0.002501	**1.27E-05**	0.499997	5.97E-07
	epialleleR	**0.002501**	1.28E-05	**0.002500**	1.27E-05	0.499997	5.96E-07
0.10%	DRAGEN	0.002661	1.37E-05	0.002662	1.36E-05	0.499835	1.73E-06
	Bismark	0.002646	1.42E-05	0.002648	1.41E-05	0.499850	1.69E-06
	methylKit	0.002662	1.37E-05	0.002664	1.36E-05	0.499835	1.75E-06
	epialleleR	**0.002624**	**1.35E-05**	**0.002626**	**1.34E-05**	**0.499872**	**1.54E-06**
0.30%	DRAGEN	0.002977	1.53E-05	0.002980	1.53E-05	0.499504	3.22E-06
	Bismark	0.002928	1.57E-05	0.002931	1.57E-05	0.499554	3.03E-06
	methylKit	0.002978	1.52E-05	0.002982	1.52E-05	0.499506	3.21E-06
	epialleleR	**0.002857**	**1.47E-05**	**0.002860**	**1.47E-05**	**0.499628**	**2.59E-06**
0.60%	DRAGEN	0.003498	1.79E-05	0.003506	1.78E-05	0.498942	1.29E-05
	Bismark	0.003393	1.81E-05	0.003402	1.81E-05	0.499051	1.25E-05
	methylKit	0.003497	1.78E-05	0.003501	1.78E-05	0.498952	1.28E-05
	epialleleR	**0.003237**	**1.66E-05**	**0.003241**	**1.65E-05**	**0.499222**	**1.14E-05**

Of note, epialleleR does not require reference sequence in order to determine the correct sequence context of cytosine bases. All observed contexts for every genomic position are counted, and the most frequent context is assumed to be correct and therefore reported. This approach allows reporting of methylation events within *de novo* (not present in the reference genome) contexts, being at the same time not affected by sequencing errors that change sequence context of cytosine bases (Supplementary Table S1).

### Sensitivity analyses

**Figure 2:**
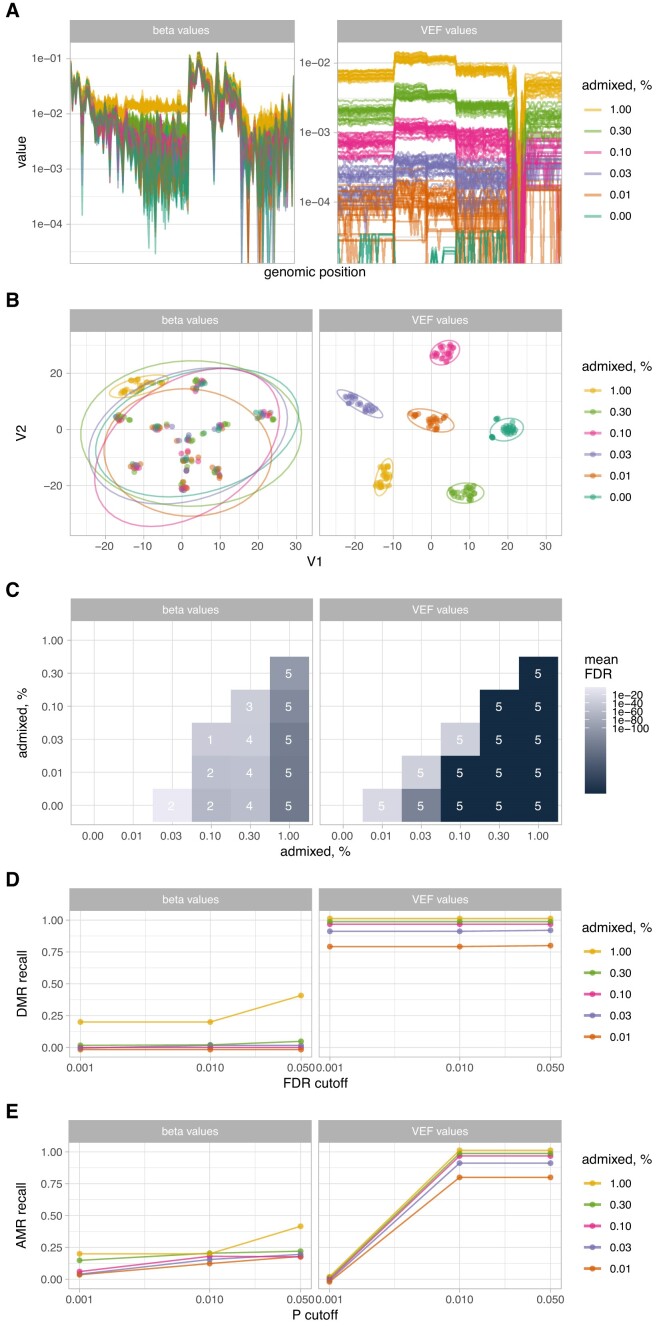
(A) Line plots of beta (left panel) and VEF (right panel) values for individual samples, color-coded according to the amount of admixed methylated reads. Each line represents a sample; y-axis, methylation value of all CpGs (*n* = 138) sorted by their genomic position (categorical x-axis). (B) Embedding plots for t-SNE analysis using beta (left panel) and VEF (right panel) values. Ellipses represent 95% confidence levels. (C) Heatmap of mean false discovery rate for differentially methylated regions (DMRs) identified by DMRcate. Labels indicate the number of DMRs found (of a total of 5 regions possible). (D) Recall rate for DMR identification using DMRcate for varying FDR cutoffs. (E) Recall rate for aberrantly methylated region (AMR) identification using ramr for varying *P* value cutoffs.

Concordantly methylated alleles (alleles with most of their CpGs having the same methylation status) may possess high biological importance [12, 23, 26]. Spontaneous 5-methyl cytosine (5mC) deamination, sequencing errors, and genuine single-nucleotide methylation/demethylation events affect observed background methylation level and can therefore hinder the detection of low-frequency hyper- or hypomethylated alleles. Differences in experimental conditions provide an additional level of variability, which can sometimes be tackled by normalization during postprocessing [37]. In contrast to the DNA methylation analysis using BeadChip arrays (such as Illumina HumanMethylation450 and HumanMethylationEPIC), which report average methylation values at the level of individual cytosines only, next-generation sequencing provides an additional data dimension by linking methylation levels of individual nucleotides within a genomic region covered by a sequencing read (epihaplotypes). However, this information is lost when methylation is assessed and reported without accounting for its allelic distribution. To evaluate the sensitivity of detection for low-frequency monoallelic hypermethylation events in next-generation sequencing data, we simulated an extended set of samples using real, amplicon-based bisulfite sequencing data for human WBCs (*n* = 10 with almost no hypermethylated alleles, as described in Materials and Methods) and fully methylated control DNA samples. Combining real WBC DNA bisulfite sequencing data allowed to introduce sample-to-sample variability although maintaining biologically relevant background methylation levels across sequenced regions, while admixing fully methylated reads simulated low-frequency, concordant methylation events. The amplicons used covered promoter regions of the tumor suppressors *MLH1, CDKN2A, MGMT, CDH1*, and *BRCA1*. The distributions of per-read beta values (Supplementary Fig. S1) and methylation patterns (Supplementary Fig. S2) of admixed samples show the expected abundance of hypermethylated (average *β* ≥ 0.5) alleles and confirm their high similarity to the real samples (Supplementary Figs. S3 and S4). Conventional cytosine reports (no read thresholding) as well as VEF reports (with read thresholding) were prepared and used for unsupervised clustering of samples and differentially methylated region (DMR) discovery. Despite quite a low overall methylation level of amplified regions (average beta value of 0.014, median of 0.005; Fig. 2A), t-distributed stochastic neighbor embedding (t-SNE) analysis based on beta values was not able to discriminate between samples with 0.01%, 0.03%, 0.10%, and 0.30% of methylated reads or no methylated reads added (Fig. 2B, left panel). On the other hand, VEF value-based t-SNE analysis resulted in spatially well-separated clusters that corresponded to each level of admixed methylated reads (Fig. 2B, right panel). Intergroup DMR discovery based on beta values (Fig. 2C, left panel) showed fewer number of regions found as well as higher associated false discovery rate (FDR), while discovery based on VEF values resulted in all 5 possible regions identified for all possible intergroup comparisons as well as generally lower associated FDR. When each sample with admixed methylated reads was compared against the group of samples without admixed methylated reads, recall metrics for differential (by DMRcate [38]; Fig. 2D) or aberrant (by ramr [21]; Fig. 2E) methylation analysis were notably higher for analyses based on VEF values (Fig. 2D, E, right panels) in comparison with analyses based on beta values (Fig. 2D, E, left panels). This shows that VEF values are more valuable for detection and analysis of low-frequency (≤1%) hypermethylation events than methylation beta values.

**Figure 3:**
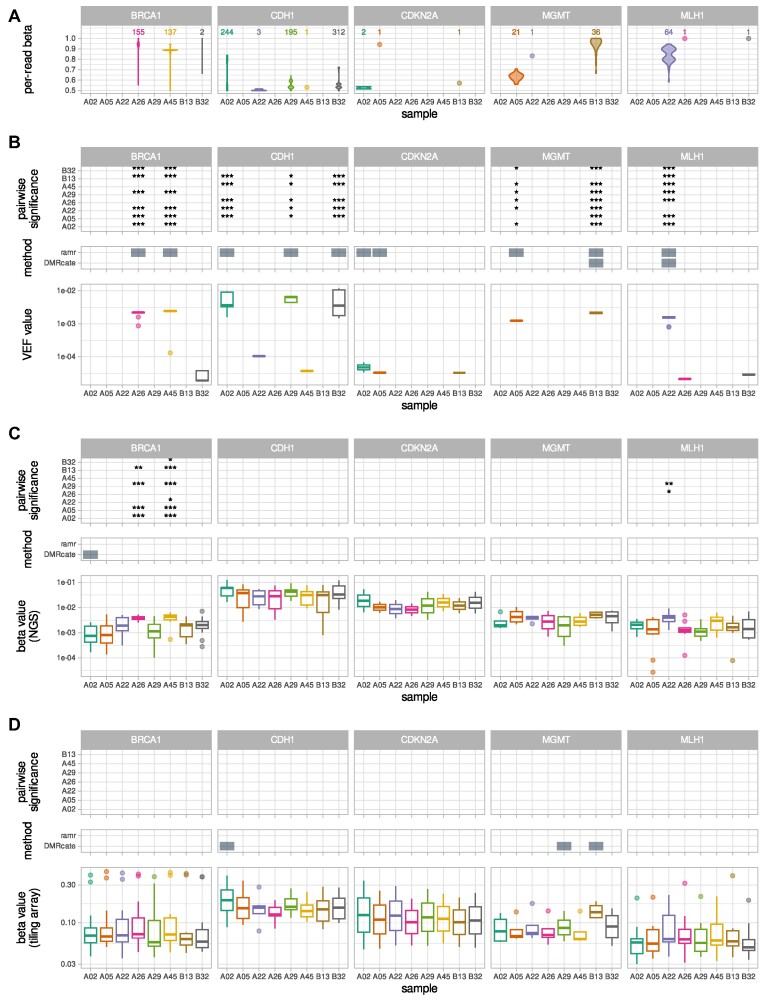
(A) Distribution of per-read beta values for NGS read pairs covering CpGs that are common for NGS and BeadChip array. For clarity, only the reads with average beta of at least 0.5 (i.e., representing hypermethylated epialleles) are included. Single observations are shown as dots; number of observations is given above. Complete density plots are provided in the Supplementary Fig. S3. Corresponding methylation patterns are provided in the Supplementary Fig. S4. (B) Lower panel: boxplots of NGS-derived VEF values for individual CpGs; middle panel: significant aberrantly or differentially methylated regions identified by ramr or DMRcate, respectively, based on VEF values; upper panel: significance levels from pairwise comparison of VEF values. (C) Lower panel: boxplots of NGS-derived beta values for individual CpGs; middle panel: significant aberrantly or differentially methylated regions identified by ramr or DMRcate, respectively, based on NGS-derived beta values; upper panel: significance levels from pairwise comparison of NGS-derived beta values. (D) Lower panel: boxplots of BeadChip array-derived beta values for individual CpGs; middle panel: significant aberrantly or differentially methylated regions identified by ramr or DMRcate, respectively, based on BeadChip array-derived beta values; upper panel: significance levels from pairwise comparison of BeadChip array-derived beta values. (B–D) The lower and upper hinges of boxes correspond to the first (Q_1_) and third (Q_3_) quartiles; the bar in the middle corresponds to the median value; the upper and lower whisker extend to Q_3_ + 1.5 * IQR and Q_1_ − 1.5 * IQR, respectively, while the values outside this range (outliers) are plotted as dots. Zero values are not plotted. ***P < 0.001, **P < 0.01, *P < 0.05, blank *P* ≥ 0.05.

BeadChip arrays, such as Illumina HumanMethylationEPIC, are another widely used, amplification-free method to assess genome-wide DNA methylation for a reduced cost. In order to directly compare the sensitivities of targeted NGS and of the BeadChip arrays for the detection of low-frequency DNA methylation events, we employed both of the methods to analyze small set of samples (*n* = 8) carrying low-frequency methylation in at least one of the assayed regions (promoter regions of *MLH1, CDKN2A, MGMT, CDH1*, and *BRCA1*). Sample distributions of per-read beta values (Supplementary Fig. S3) and methylation patterns (Supplementary Fig. S4) show that these samples indeed contain varying frequencies of hypermethylated (average *β* ≥ 0.5) alleles. For unbiased comparison, we limited the corresponding data sets to the CpGs assayed and sufficiently covered by both techniques. Analysis revealed that VEF values of samples with many hypermethylated alleles (e.g., A26 and A45 for *BRCA1*; as apparent from Fig. 3A) differ significantly (Fig. 3B) from VEF values of samples with only a few or no hypermethylated alleles (e.g., A02 or A05 for *BRCA1*; Fig. 3A). When VEF values were used for identification of aberrantly methylated regions (AMRs) or DMRs by ramr [21] or DMRcate [38], respectively, the significant regions found correlated well with the notable presence of hypermethylated alleles. Of note, slightly inferior performance of DMRcate is probably due to the fact that for some of the genomic regions, too many samples in this subset simultaneously contained hypermethylated epialleles. When DMRcate was used for the same purpose on an extended set of sequenced samples (*n* = 18, containing *n* = 10 samples characterized by the absence of hypermethylated alleles that were used to create the admixed sample set), its performance in identification of hypermethylated epiallele-containing samples was higher (Supplementary Fig. S5).

In contrast, only a few significant differences remained when NGS beta values were used for sample comparison (Fig. 3C), while pairwise comparisons based on BeadChip array beta values did not reveal any significant differences between samples (Fig. 3D). The search for aberrantly or differentially methylated regions using either NGS or array beta values did not result in identification of such regions in relevant (according to methylation patterns or beta value densities) samples. Generally higher beta values of BeadChip array as compared to NGS beta values likely mask subtle changes in methylation caused by the presence of infrequent hypermethylated alleles and hinder the detection of differences between samples.

Several scores to describe and quantify variability in DNA methylation in sequencing reads (within-sample heterogeneity [WSH]) have been proposed [39]. In order to assess WSH, we evaluated the difference in combinatorial entropy between each pair of samples using methclone [40] (Supplementary Fig. S6A). The largest (by absolute value) reported difference in combinatorial entropy of −2.59 between any pair of samples confirms a high similarity between sample methylation profiles, of note, being much smaller than cutoffs for epiallele shifts between samples analyzed in [39] (−60 and lower). Further, we also calculated 4 additional heterogeneity scores: combinatorial entropy, epipolymorphism, fraction of discordant read pairs (FDRP), and proportion of discordant reads (PDR). The scores themselves (Supplementary Fig. S6B) and the levels of score-based pairwise significance between samples (Supplementary Fig. S6C) are not generally consistent with fractions of hypermethylated (average *β* ≥ 0.5) alleles (Fig. 3A and Supplementary Fig. S3) or VEF values (Fig. 3B): for example, samples A26 and A45 have a notable fraction of hypermethylated reads in the *BRCA1* promoter region compared to other samples, although it is not reflected at the level of WSH scores. Importantly, WSH scores produced cannot be directly used as an input for DMR analysis tools, which are commonly employed to characterize exact differences in methylation between samples.

It is known that DNA methylation profiles of blood samples depend on the varying contribution of individual blood cell types [41, 42]. While we cannot exclude that hypermethylated alleles present in the samples analyzed here originate from a particular blood cell type, low-level, mosaic epimutations of at least *BRCA1* were previously shown to be independent of blood subfraction composition [10]. Of note, only 1 CpG (cg05785947 in *CDH1*) out of 37 used in NGS versus BeadChip array comparison here was found to be significantly differentially methylated between blood cell types of healthy males, and none of the CpGs were significantly differentially methylated between blood cell types of newborns.

### Processing speed analyses

**Figure 4:**
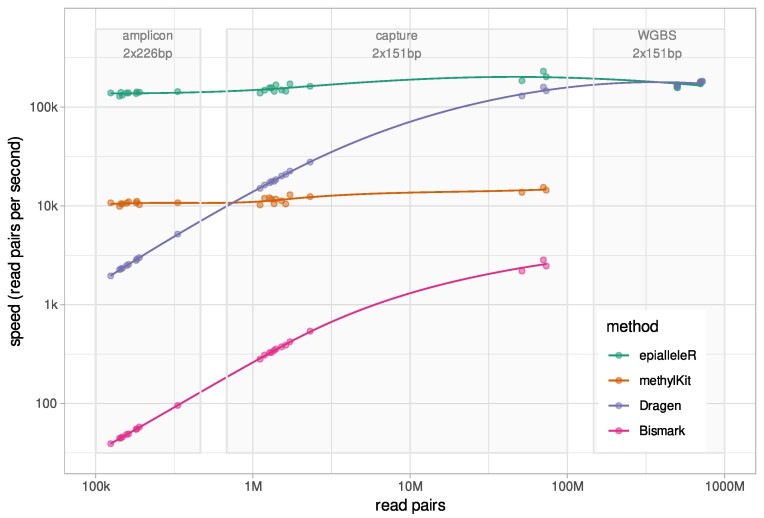
Data-processing speed (in read pairs per second) of epialleleR as compared to 3 other methods for methylation reporting (methylKit, Bismark, and DRAGEN Bio-IT Platform). Read count (in number of pairs) is given at x-axis; light gray boxes outline data obtained by targeted amplicon-based, genome-wide capture-based, or whole-genome bisulfite sequencing.

Methylation sequencing data produced by contemporary techniques vary in scale and depth and may contain several thousands to billions of single or paired-end reads. To analyze them efficiently, computational methods must be scalable and fast enough for as large as possible range of sample counts or data file sizes. Unfortunately, many academic tools use computationally complex algorithms that do not scale to contemporary tasks. We compared data-processing speed for epialleleR versus methylKit, Bismark, and DRAGEN Bio-IT Platform, performing exactly the same task (BAM file to cytosine report) of methylation reporting across input data coming from various assays: amplicon based (*n* = 10 samples with a depth of coverage of ∼20,000×), genome-wide capture based (*n* = 10 with a depth of coverage of ∼60× and *n* = 3 with a depth of coverage of ∼1,000×), or whole-genome bisulfite sequencing (WGBS, *n* = 6 with a depth of coverage of ∼60×). The obtained results confirm very efficient implementation of epialleleR and its suitability for analysis of datasets of any depth and coverage (Fig. 4, Table 1).

## Discussion

While conflicting data have linked low-level mosaic primary constitutional epimutations to cancer risk for more than a decade [43], we have recently obtained firm evidence implicating primary epimutations within the *BRCA1* gene in an elevated risk of incident breast and ovarian cancer [12]. The assumption that such epimutations may affect other tumor suppressor genes and, therefore, lead to other cancer forms [43] institutes a new research area with respect to cancer risk. Further, the findings of such epimutations in umbilical cord blood [10, 23] indicate prenatal events of a yet unknown genesis. This creates the need for multidisciplinary studies on the mechanisms of these events and on their effects in respect to cancer risk, as well as the need for ultrasensitive methods allowing sample assessment at a high scale.

Here, we present the details on a fast, accurate, and sensitive method to detect, quantify, and visualize epialleles in NGS data. The method shows its superiority versus conventional methods of methylation reporting, especially when applied for detection of low-frequency methylation events, as it is by design less susceptible to variations in conversion efficiency or sequencing quality. Although epialleleR is not a differential methylation analysis tool, its output can be directly used to group samples based on their methylation profiles (by applying a simple threshold as in [12, 23] or using unsupervised clustering), as well as an input for other differential/aberrant methylation analysis software (the latter is not possible for WSH analysis tools).

The default epialleleR parameters that were used for read thresholding in the present and linked studies [12, 23] are sought to be optimal for the detection of aberrant hypermethylation events within normally unmethylated genomic regions such as CpG-rich regulatory regions of tumor suppressor genes. If the nature of regions of interest deviates from the one described above, methylation characteristics can be explored using other epialleleR methods (e.g., extractPatterns), and thresholding parameters can be adjusted to detect desired methylation events.

We thoroughly tested epialleleR using bisulfite sequencing data; the method, however, can also be applied to analyze and compare data obtained using any methylation sequencing technique (reduced representation bisulfite sequencing [RRBS], oxidative bisulfite sequencing [oxBS-seq], and Tet-assistant bisulfite sequencing [TAB-seq]), as long as methylation in these data can be called at individual cytosine residues instead of being analyzed by comparing relative abundance of the fragments (such as for methylation sensitive restriction enzyme sequencing [MRE-seq] or methylated DNA immunoprecipitation sequencing [MeDIP-seq]).

The possibility to call cytosine methylation for alignment files created by different short sequence aligners and subtle though noticeable changes in cytosine reporting accuracy, together with immense speed gain, make epialleleR a method of choice not only for discovery of infrequent hypermethylated epialleles (as in [12, 23]) but also as a tool to produce conventional (no read thresholding) cytosine reports from any methylation sequencing alignment files.

The implemented method is fully documented and can be easily used from within the R environment for statistical computing. With the epialleleR already revealing its suitability for detection of low-level mosaic methylation events in a large dataset [12, 23], we believe it constitutes an optimal tool for assessment of low-level mosaic epimutations with respect to risk of cancer as well as other diseases of relevance.

## Conclusions

Here, we present epialleleR, a very fast, accurate, and sensitive method to detect, quantify, and visualize epialleles in NGS data. Efficient implementation and improvements in cytosine reporting accuracy allow us to recommend epialleleR not only for analysis of methylation patterns and to enhance low-level differentially methylated region discovery but also as a conventional cytosine reporting tool for various kinds of methylation sequencing data. The epialleleR R/Bioconductor package is freely available at [44, 45].

## Materials and Methods

### Next-generation sequencing

WBC DNA samples from anonymized males (*n* = 88) [46, 47] and human HCT116 DKO methylated DNA control sample (Zymo Research, cat. D5014-2) were bisulfite converted, and 5 DNA fragments, representing promoter regions of 5 established tumor suppressor genes, were amplified using a custom set of primers (GRCh38 assembly coordinates of assayed regions: *MLH1*, chr3:36993123–36993500; *CDKN2A*, chr9:21974554–21974921; *MGMT*, chr10:129467118–129467477; *CDH1*, chr16:68737102–68737469; *BRCA1*, chr17:43125171–43125550), indexed, and sequenced similarly to as previously described [12] (GSE201688). The resulting average coverage was 5,000× to 50,000× per amplicon.

### Bioinformatic and statistical analyses

Massive parallel sequencing (NGS) reads were mapped/aligned to the GRCh38 human reference genome, and the methylation was called using Illumina DRAGEN Bio-IT Platform (v3.9.5) with the following parameters: --methylation-mapping-implementation single-pass, --enable-methylation-calling true, --methylation-generate-cytosine-report false, --methylation-protocol nondirectional, and --enable-sort false, unless stated otherwise. R software environment for statistical computing (v4.1.2) was used for all downstream statistical analyses.

The frequency of hypermethylated alleles across assayed regions in *n* = 88 male WBC DNA samples was estimated using epialleleR::generateAmpliconReport with the following parameters: min.mapq=30, min.baseq=20, nthreads=4, threshold.reads=TRUE, report.context=“CG,” and bed.file pointing to a location of a BED (browser extensible data) file with genomic regions amplified (see amplicon coordinates above). Two sample subgroups (*n* = 8 and *n* = 10) were selected for sensitivity analyses based on the frequencies of hypermethylated alleles as explained below.

### Cytosine reporting accuracy comparison

Four sets of paired-end sequencing reads (151 bp, 50 million read pairs each set) were simulated using Sherman Bisulfite FastQ Read Simulator (RRID:SCR_001294) [48] with the following options: --length 151, --number_of_seqs 50000000, --paired_end, --minfrag 70, --maxfrag 400, --CG_conversion 0, --CH_conversion 99.5, and varying sequencing error rate (--error_rate parameter) of 0%, 0.1%, 0.3%, or 0.6%. The quality scores of these simulated sequences followed an exponential decay curve, which resulted in a higher number of base errors toward the 3′-end of the read (as seen in real data). Human chromosome 19 sequence (GRCh38.p13 NC_000019.10, 58,617,616 bp, 1,105,620 forward strand CpGs) was used as a reference genome for read simulation and mapping/alignment due to its highest CpG content across all human chromosomes [49] and in order to maintain optimal balance of analysis speed and base coverage. Each set of reads was then duplicated, and all read1 cytosines (C) and read2 guanines (G) in any context in the duplicate sets were replaced with thymines (T) and adenines (A), respectively. Then, duplicate sets (i.e., unmethylated reads) were merged with original sets (i.e., methylated reads), resulting in 4 sets of reads 100 million pairs each, with the cytosine conversion rate of exactly 50% and about 99.75% in CG and non-CG contexts, respectively.

The mapping and alignment of simulated reads were performed using Illumina DRAGEN Bio-IT Platform v3.9.5 with the following modification in parameters: --methylation-protocol directional. Methylation reporting by all tools was done as described below (reporting parameters in Speed comparison section).

### Sensitivity comparison on admixed samples

In order to simulate variable methylation levels while maintaining biological heterogeneity of the samples, we selected 10 male DNA NGS samples with the lowest frequency of hypermethylated alleles across all assayed regions, then admixed varying fractions of reads from 2 random samples and additionally “spiked” a certain number of fully methylated reads from the methylated DNA control sample. This resulted in 150 samples containing 0%, 0.01%, 0.03%, 0.1%, 0.3%, or 1% of methylated reads per sample (25 samples per every category).

Read mapping, alignment, methylation calling, and generation of genome-wide cytosine reports were performed using the Illumina DRAGEN Bio-IT Platform as described above. VEF calling was performed using epialleleR::generateCytosineReport with the following parameters: min.mapq=0, min.baseq=0, nthreads=4, threshold.reads=TRUE, and report.context=“CG.”

Methylation patterns and per-read beta values for all samples were extracted using epialleleR::extractPatterns with the following parameters: min.mapq=30, min.baseq=20, nthreads=4, clip.patterns=FALSE, and bed.file pointing to a location of the BED file with genomic regions amplified (see amplicon coordinates above).

Barnes–Hut t-SNE analysis was performed using R package Rtsne v0.15 [50] and matrices of beta or VEF values for all genomic positions of CpGs with the coverage of at least 1,000× and available values for all analyzed samples (total number of CpGs, *n* = 138; *MLH1, n* = 20; *CDKN2A, n* = 35; *MGMT, n* = 33; *CDH1, n* = 32; *BRCA1, n* = 18).

### Sensitivity comparison to methylation array data

Eight additional WBC DNA NGS samples from anonymized males carrying hypermethylated alleles in at least one of the assayed regions were selected, and VEF calling was performed using epialleleR::generateCytosineReport with the following parameters: min.mapq=30, min.baseq=20, nthreads=4, threshold.reads=TRUE, and report.context=“CG.” The same DNA samples were also bisulfite converted using the Zymo EZ DNA Methylation Kit (Zymo Research, cat. D5001), and genome-wide methylation levels were assessed using Illumina HumanMethylationEPIC BeadChip arrays according to the manufacturer’s instructions. Resulting IDAT files were processed (normalized and annotated) with the minfi Bioconductor package [37] using the preprocessQuantile method with outlier thresholding enabled (GSE201689). For direct comparison, only the CpGs that are covered in all samples by both BeadChip arrays (*P* value of 0) and targeted sequencing (minimum sequencing coverage of 5,000×) were retained (*MLH1, n* = 10; *CDKN2A, n* = 2; *MGMT, n* = 4; *CDH1, n* = 7; *BRCA1, n* = 14). Pairwise sample comparison was performed using a *t*-test with Holm adjustment for multiple comparisons.

The sets of CpGs that are differentially methylated between cell blood types were reported previously: DNA methylation profiles for 6 blood cell types from 6 males [41, 51] and DNA methylation profiles for 7 blood cell types from cord blood of 104 newborns [42, 52]. CpG-level differential methylation analysis *P* values were Holm-adjusted, and the ones that remained significant (adjusted *P* ≤ 0.05; *n* = 73,629 of total 456,655 for male blood data set; *n* = 221,246 of total 429,794 for newborn cord blood data set) were checked for overlap with the set of CpGs analyzed in this study (*n* = 35 CpGs of total *n* = 37 were present in each of male/newborn datasets).

### Differential methylation analysis

DMRs were called using R package DMRcate (v2.12.0) with the following parameters: lambda=1000, min.cpgs=2, and pcutoff=“fdr” [38]. AMRs were called using R package ramr (v1.6.0) with the following parameters: ramr.method=“beta,” min.cpgs=2,and merge.window=500 [21]. To enable maximum likelihood estimation of beta distribution parameters, all zeros were replaced with minimum double values (2.26e-308).

For intergroup DMR discovery in admixed samples, pairwise comparison of sample groups defined by the number of admixed reads was performed (*n* = 25 samples in each group) using the default level of the FDR cutoff (equals 0.05). For DMR discovery in real samples, as DMRcate methods require 2 classes/categories for comparison, every real sample from the test dataset was tested against all the other samples using the default FDR cutoff value.

To assess DMR (or AMR) recall metrics in admixed samples, every sample with admixed reads was compared using DMRcate (or ramr) to the group of 25 samples without admixed reads at a varying level of FDR (or *P* value) cutoff of 0.05, 0.01, or 0.001. As the admixed reads covered all 5 assayed regions, only the total number of real positive (P) regions (equals 5 for each comparison), the number of true-positive (TP) regions, and the number of false-negative (FN) regions (FN = P – TP) were known, while the numbers of true-negative (TN) or false-positive (FP) regions were undefined. Therefore, recall, or true positive rate (TPR = TP/P), was chosen as a sensitivity metric.

### Within-sample heterogeneity

Estimation of WSH was performed on 8 samples used in the sensitivity comparison between array- and NGS-based methylation profiling. Difference in entropy was evaluated using methclone (v1) [40] with a distance cutoff of 500 and minimum read coverage of 1,000 for every pair of samples. As methclone outputs values for multiple genomic regions, the minimum value (representing absolute largest difference) was selected and used further. Entropy, epipolymorphism, FDRP, and PDR were evaluated using R package WSH (v0.1.6) [39] with the following options: mapq.filter=30, window.size=500, and bam.file pointing to a location of the BAM file. Due to exponential complexity of the FDRP calculation, option max.reads was set to 100 for FDRP calculation and to 1e+06 otherwise. Pairwise sample score comparison was performed using a *t*-test with Holm adjustment for multiple comparisons.

### Processing speed comparison

Comparison of processing speed was performed on 29 BAM files containing paired-end alignments and methylation calls derived from bisulfite sequencing of human WBC DNA samples prepared using the following assays: (A) amplicon-based sequencing of promoter regions of the *BRCA1* gene (*n* = 10 files, 0.12–0.33 million read pairs per file, average coverage of ∼20,000×) [12], (B) genome-wide capture-based bisulfite sequencing of promoter regions of 283 tumor suppressor genes (*n* = 10 files, 1.11–2.31 million read pairs per file, average coverage of ∼60×, and *n* = 3 files, 51.4–73.4 million read pairs per file, average coverage of ∼1,000×) [53, 54]. and (C) whole-genome bisulfite sequencing (*n* = 6 files, 497–723 million read pairs per file, average coverage of ∼60×; epialleleR and Illumina DRAGEN Bio-IT Platform only). The 2 former data sets (A and B) were generated in house and described previously, while the latter data (C) were obtained from NCBI Sequence Read Archive (GEO/SRA samples GSM3683953/SRX6640720, GSM3683958/SRX6640725, GSM3683965/SRX6640732, GSM3683951/SRX6640718, GSM3683955/SRX6640722, and GSM3683962/SRX6640729) and reported elsewhere [55].

Processing times to produce conventional cytosine reports were recorded as following:

Bismark CX methylation reports were created using Bismark v0.22.3 (RRID:SCR_005604) [14] with the following parameters: command bismark_methylation_extractor, --paired-end, --no_overlap, --comprehensive, --gzip, --mbias_off --parallel 8, --cytosine_report, --CX, and --buffer_size 64G. Genome-wide cytosine methylation report but not bedGraph report was chosen in order to obtain results of highest quality (not affected by sequencing errors). As parallel processing was requested, Bismark used up to 24 cores for some of its subtasks.

methylKit CX methylation reports were created using R/Bioconductor package methylKit v1.20.0 (RRID:SCR_005177) [35] with the following parameters: function methylKit::processBismarkAln, minqual=0, mincov=0, save.context=c(“CpG,”“CHG,”“CHH”), nolap=TRUE, and location pointing to the location of a BAM file. Parallel processing is currently not available for methylKit::processBismarkAln.

epialleleR CX methylation reports were created using R/Bioconductor package epialleleR v1.3.5 with the following parameters: function epialleleR::generateCytosineReport, min.mapq=0, min.baseq=0, nthreads=4 (number of HTSlib decompression threads), threshold.reads=FALSE, report.context=“CX,” and bam pointing to the location of a BAM file. epialleleR methods currently run in a single-threaded mode only but can benefit from additional BAM decompression threads provided by HTSlib.

Illumina DRAGEN is a hardware solution that relies on the presence of the FPGA accelerator card, which precludes DRAGEN software execution on other platforms. At the same time, outdated software development tools available at DRAGEN (GCC v4.8.5, R v3.6.0) impede installation of third-party software and R/Bioconductor packages and may potentially affect their performance. Therefore, testing of methylation reporting tools was carried out in 2 different settings.

Bismark, methylKit, and epialleleR were tested on the workstation equipped with an AMD EPYC 7742 64-core processor, 512 GB of memory, and the Red Hat Enterprise Linux Server release 7.9 (Developer Toolset 6, GCC v6.3.1), with BAM files retrieved from high-speed (10 Gbps) network-accessible storage.

DRAGEN CX methylation reports were created using Illumina DRAGEN Bio-IT Platform v3.9.5 (Intel Xeon Gold 6126 48-core processor, 256 GB of memory, and CentOS Linux release 7.5.1804) with the following parameters: --methylation-generate-cytosine-reports=true, --enable-sort=false, --enable-duplicate-marking=false, --methylation-report-only=true, and --bam-input pointing to the location of a BAM file. Default number of threads (up to 24) was used for data processing using DRAGEN; BAM files were accessed from a local, high-speed NVMe solid state disk.

For Bismark and DRAGEN, elapsed time measurements were stably reproducible, and thus processing time was recorded only once for each file. For methylKit and epialleleR, the tests were run 5 times in sequential random order by means of R package microbenchmark v1.4.9, and the average time was used in comparison to mitigate variability in processing time measurements.

## Availability of Source Code and Requirements

The epialleleR R/Bioconductor package (biotools:epialleleR, RRID:SCR_023913) is freely available at https://bioconductor.org/packages/epialleleR/ and https://github.com/BBCG/epialleleR. The R scripts used in this manuscript are freely available at DataverseNO (https://doi.org/10.18710/2BQTJP).

Project name: epialleleR

Project homepage: https://github.com/BBCG/epialleleR

Bioconductor: https://bioconductor.org/packages/epialleleR/

Operating system: Linux, macOS, Windows

Programming language: R, C, C++

Other requirements: C++17, GNU make

License: Artistic-2.0

biotools: epialleleR

RRID: SCR_023913

Version 1.3.5 of the epialleleR R/Bioconductor package was used [32]. A previous version of this article was deposited in bioRxiv (doi: 10.1101/2022.06.30.498213) and the epialleleR has been applied in [12, 23] with data available at NCBI Gene Expression Omnibus under accession number GSE243966.

## Supplementary Material

giad087_GIGA-D-23-00149_Original_Submission

giad087_GIGA-D-23-00149_Revision_1

giad087_GIGA-D-23-00149_Revision_2

giad087_GIGA-D-23-00149_Revision_3

giad087_GIGA-D-23-00149_Revision_4

giad087_Response_to_Reviewer_Comments_Original_Submission

giad087_Response_to_Reviewer_Comments_Revision_1

giad087_Response_to_Reviewer_Comments_Revision_2

giad087_Response_to_Reviewer_Comments_Revision_3

giad087_Reviewer_1_Report_Original_Submission Stefan Roeder -- 6/28/2023 Reviewed

giad087_Reviewer_2_Report_Original_Submission Keegan Korthauer -- 7/21/2023 Reviewed

giad087_Reviewer_2_Report_Revision_1 Keegan Korthauer -- 8/14/2023 Reviewed

giad087_Reviewer_2_Report_Revision_2 Keegan Korthauer -- 9/8/2023 Reviewed

giad087_Supplemental_Files

## Data Availability

The data underlying accuracy and sensitivity analyses are freely available at DataverseNO [56]. Sensitive data used for the processing speed assessment are available from the authors in accordance with study protocols. Public data for sensitivity analysis have been deposited at NCBI Gene Expression Omnibus under accession number GSE201690. Public whole-genome bisulfite sequencing data used for the processing speed assessment are available at NCBI Sequencing Read Archive under accession number SRP217135.

## Additional Files

**Supplementary Fig. S1**. Scaled density of per-read beta values from all admixed samples combined, split by the level of admixed reads and genomic region of interest. The y-axis is scaled to 1 and limited to 0.015. The hypermethylated (β ≥ 0.5) reads are increasingly apparent on the right sides of plots in accordance with an increase in admixed methylated reads.

**Supplementary Fig. S2**. Methylation patterns from all admixed samples combined, split by the level of admixed reads and genomic region of interest. Lines depict patterns, and open and closed circles depict unmethylated and methylated cytosines, respectively. Numbers on the right of every pattern indicate how many times each pattern occurs for every given gene/sample set. Due to very high number of methylation pattern types, only the most abundant pattern (if any) is shown for each range of average beta value: [0,0.2), [0.2,0.4), [0.4,0.6), [0.6,0.8), [0.8,1]. The hypermethylated (β ≥ 0.5) patterns are increasingly apparent at the top of plots in accordance with an increase in admixed methylated reads.

**Supplementary Fig. S3**. Scaled density of per-read beta values from *n* = 8 real samples used to compare sensitivity of methylation profiling by NGS and array, split by sample and genomic region of interest. The y-axis is scaled to 1 and limited to 0.015. The population of hypermethylated (β ≥ 0.5) reads that are apparent in the Fig. 3A in the main text are pointed to by black arrows.

**Supplementary Fig. S4**. Methylation patterns from *n* = 8 real samples used to compare sensitivity of methylation profiling by NGS and array, split by sample and genomic region of interest. Lines depict patterns, and open and closed circles depict unmethylated and methylated cytosines, respectively. Numbers on the right of every pattern indicate how many times each pattern occurs for every given gene/sample set. Due to very high number of methylation pattern types, only the most abundant pattern (if any) is shown for each range of average beta value: [0,0.2), [0.2,0.4), [0.4,0.6), [0.6,0.8), [0.8,1]. The hypermethylated (β ≥ 0.5) patterns represent hypermethylated epialleles that are present in certain samples/regions, as shown in Fig. 3A in the main text and Supplementary Fig. S3.

**Supplementary Fig. S5**. (A) Distribution of per-read beta values for NGS reads covering CpGs that are common for NGS and BeadChip array. For clarity, only the reads with average beta of at least 0.5 (i.e., representing hypermethylated epialleles) are included. Single observations are shown as dots; number of observations is given above. (B) Lower panel: boxplots of NGS-derived VEF values for individual CpGs; upper panel: significant aberrantly or differentially methylated regions identified by ramr or DMRcate, respectively, based on VEF values. (C) Lower panel: boxplots of NGS-derived beta values for individual CpGs; upper panel: significant aberrantly or differentially methylated regions identified by ramr or DMRcate, respectively, based on NGS-derived beta values. (B, C) The lower and upper hinges of boxes correspond to the first (Q1) and third (Q3) quartiles; the bar in the middle corresponds to the median value; the upper and lower whisker extend to Q3 + 1.5 * IQR and Q1 − 1.5 * IQR, respectively, while the values outside this range (outliers) are plotted as dots. Zero values are not plotted. The coloring is preserved for *n* = 8 samples used in Fig. 3 of the main text. The *n* = 10 samples used to create admixed samples and not included in Fig. 3 are plotted in light gray.

**Supplementary Fig. S6**. (A) Heatmap of minimum (equals absolute largest) difference in combinatorial entropy for all pairs of samples, split by genomic region of interest. (B) Heatmap of combinatorial entropy, epipolymorphism, fraction of discordant read pairs (FDRP), and proportion of discordant reads (PDR), split by genomic region of interest. (C) Heatmap of *P* values for pairwise comparison of samples using WSH scores, split by score and genomic region of interest. ****P* < 0.001, ***P* < 0.01, **P* < 0.05, blank *P* ≥ 0.05.

**Supplementary Table S1**. Complete metrics of cytosine reporting across all 3 possible cytosine genomic contexts (CHH, CHG, and CpG), obtained using simulated chr19 reads with varying sequencing error rate by selected tools. “reported,” number of cytosines present in the cytosine reports; “valid context,” number of cytosines for which genomic context was correctly identified; “invalid context,” number of cytosines for which genomic context was incorrectly identified; “not covered,” number of cytosines not present in a cytosine report; “mean coverage,” average coverage of all cytosines in this context; “mean” and “variance,” average value and variance for beta values of all cytosines in this context; “is 0.5,” number of cytosines with beta value of exactly 0.5 (ground truth for this dataset); “is not 0.5,” number cytosines with beta value not equal to 0.5.

## Abbreviations

AMR: aberrantly methylated region; BAM: binary sequence alignment/map; BED: browser extensible data; CpG: cytosine followed by a guanine; DMR: differentially methylated region; FDR: false discovery rate; FDRP: fraction of discordant read pairs; IQR: interquartile range; NGS: next-generation sequencing; PDR: proportion of discordant reads; t-SNE: t-distributed stochastic neighbor embedding; VCF: variant call format; VEF: variant epiallele frequency; WBC: white blood cell; WGBS: whole-genome bisulfite sequencing; WSH: within-sample heterogeneity.
