# Supplementary material for: epialleleR: an R/Bioconductor package for sensitive allele-specific methylation analysis in NGS data
Source: Gigascience. 2023 Oct 31;12:giad087. doi: 10.1093/gigascience/giad087 (PMC10622323; doi:10.1093/gigascience/giad087)
Supplement: giad087_GIGA-D-23-00149_Revision_1 [file giad087_giga-d-23-00149_revision_1.pdf]

GigaScience

epialleleR: an R/Bioconductor package for sensitive allele-specific methylation analysis in NGS data

--Manuscript Draft--

Manuscript Number:	GIGA-D-23-00149R1
Full Title:	epialleleR: an R/Bioconductor package for sensitive allele-specific methylation analysis in NGS data
Article Type:	Research
Funding Information:	Stiftelsen Kristian Gerhard Jebsen (SKGJ-MED-020) — Dr. Per Eystein Lønning<br>Kreftforeningen (190281-2017) — Dr. Stian Knappskog<br>Norges Forskningsråd (617344-1) — Dr. Per Eystein Lønning
Abstract:	Low-level mosaic epimutations within the BRCA1 gene promoter occurs in 5–8% of healthy individuals and is associated with a significantly elevated risk of breast and ovarian cancer. Similar events may also affect other tumour suppressor genes, potentially being a significant contributor to cancer burden. While this opens a new area for translational research, detection of low-level mosaic epigenetic events requires highly sensitive and robust methodology for methylation analysis. We here present epialleleR, a computational framework for sensitive detection, quantification and visualisation of mosaic epimutations in methylation sequencing data. Analysing simulated and real data sets, we provide in-depth assessments of epialleleR performance, and show that linkage to epihaplotype data is necessary to detect low-level methylation events. The epialleleR is freely available at https://github.com/BBCG/epialleleR and https://bioconductor.org/packages/epialleleR/ as an open source R/Bioconductor package.
Corresponding Author:	Oleksii Nikolaienko<br>University of Bergen<br>Bergen, Hordaland NORWAY
Corresponding Author Secondary Information:	
Corresponding Author's Institution:	University of Bergen
Corresponding Author's Secondary Institution:	
First Author:	Oleksii Nikolaienko
First Author Secondary Information:	
Order of Authors:	Oleksii Nikolaienko<br>Per Eystein Lønning<br>Stian Knappskog
Order of Authors Secondary Information:	
Response to Reviewers:	Dear Dr. Nogoy,<br><br>Thank you for the thorough consideration of our manuscript GIGA-D-23-00149 and for giving us the opportunity to submit a revised version of the manuscript.<br><br>We find the comments from Reviewers constructive and useful. In particular, we found that the majority of the comments from Reviewer 2 were due to our phrases, that we realize were imprecise, at different places in the original manuscript. By addressing these comments, we feel we have made the manuscript much clearer for the readers and that there should be no room for any misunderstandings.

Powered by Editorial Manager® and ProduXion Manager® from Aries Systems Corporation

All changes are marked in the revised document and a point-by-point explanation for our revisions according to each of the Reviewers’ comments is additionally attached in the document “Response-to-reviewers.pdf”.

In addition to the revisions, we have also registered epialleleR at bio.tools and Research Resource Identification Portal and the relevant IDs are now included in the availability section of the manuscript.

We hope the revised version of our manuscript will be suitable for publication in GigaScience and look forward to your response in due time.

On behalf of the authors,
Oleksii Nikolaienko, PhD

We thank the Reviewers for their constructive feedback. Below, we have provided comments to each of the Reviewers’ questions and explained how we have revised the manuscript accordingly.

Reviewer #1:
The authors provide information and software (epialleleR) on a method to detect, quantify and visualize low-frequency hypermethylation events in NGS-based methylation data together with data showing the ability to link BRCA1 epimutations and cancer risk.

epialleleR is fully adjustable (parameter thresholds) to end-user needs. In the results there is shown, that the level of methylation per genomic position (Variant Epiallele Frequency (VEF)) is better suited for DMR (differentially methylated region) detection than FDR (false discovery rate) based methods.

For me, it seems uncommon to have the section "Material and Methods"at the end of the article, after the sections "Results", "Discussion" and "Conclusion"; but this is only a minor remark and is to authors discretion.

A: We do not have any preference for the order of sections. We see that many articles in the Journal have their “Materials and Methods” section at their end, but we would appreciate advice on this from the Editor and will of course change the order of sections if the Editor wishes.

I wonder, why runtime comparisons were executed in different environments (DRAGEN CX was different than Bismark, methylKit and epialleleR)? This makes it difficult to really compare efficiacy of packages. Maybe, the authors can add a statement on the reason for this.

A: We understand that this was unclear in the original manuscript. A practical problem for this comparison is that Illumina DRAGEN is a hardware solution that relies on the presence of FPGA accelerator card, which precludes DRAGEN software execution on other platforms. On the other hand, Illumina DRAGEN operating system (Red Hat Enterprise Linux 7 with GCC4.8, R3.6.0) is locked by vendor and quite outdated, which makes it very difficult to install recent development tools, as well as R environment and Bioconductor packages. Therefore, we had two use two different environments, although Bismark, methylKit and epialleleR were compared on the very same workstation. This explanation is now added in the “Processing speed comparison” paragraph in the Materials and Methods section of the revised manuscript.

Overall, I recommend to accept this article.

Reviewer #2: Review of GIGA-D-23-00149

The manuscript presents a computational approach for detecting what the authors term "mosaic epimutations". However, I found it very difficult to review the merits of the contribution owing to some fundamental issues with presentation, missing key details that motivate/justify the work, and problematic choices for evaluation/comparison. Below I list detailed concerns, separated by major and minor.

A: We have now provided additional clarifications in the text of the manuscript as well as in the comments below and we would be happy if Reviewer 2 will consider the manuscript again.

Major:

The manuscript lacks clarity in the presentation of the scientific question. The authors need to state their definition of mosaic methylation, and in addition how it relates to allele-specific methylation.

A: We agree that we could be more clear in these statements and in the revised version we consistently use the term "mosaic epimutations" instead of "mosaic methylation" in order to avoid confusion. In addition, we have provided simple statements defining the terminology in the text (e.g definition of "mosaic" in the introduction). In the Results section, we have also added a statement explaining the need for single-nucleotide variants to enable allele-specific methylation calls, clarifying how such allele-specific calls can be performed using epialleleR.

The motivation for development of new methodology as presented is somewhat contradictory. If the previously identified DNA methylation changes are so small (0.03-1%), and, as the authors state, "indistinguishable from the background methylation level", then how were they detected in the first place? Further, the analysis task is described by the authors as "trivial".

A: We understand that the order of events during the development of the methodology may be confusing. The fact is that we first discovered the qualitative presence of low-level mosaic epimutations in BRCA1 by methylation specific qPCR and used this method for our initial case-control assessment (doi: 10.7326/M17-0101). However, given the low accuracy of qPCR in very high Cp-numbers (in the range of 38-42), we saw it as imperative to develop a high-depth NGS-based assay capable of delivering high sensitivity, methylation calls on individual CpGs as well as allele-specific information. Given the breakthrough findings regarding the biological relevance of low-level mosaic epimutations, we saw it as critical to the field to provide a method by which such epimutations could be detected and quantified reliably.

The specific contributions of this manuscript are unclear. The manuscript claims to present a computational framework, but also cites published work from the same authors that appears to have already used the framework (Lønning et al. 2022). If this is the case, this disclosure should be made more transparent.

A: The present manuscript was deposited and appeared online prior to publication of the Lønning et al., 2022 paper which cites this manuscript. While they are linked, this manuscript describes the computational method and its benefits as compared to other tools, and is aimed at bioinformaticians, while the subsequent Lønning et al., 2022 paper and another preprint (doi:10.1101/2023.05.14.23289949) describe experimental findings obtained by using this method and are aimed at clinical oncologists. In the revised manuscript, we have added this disclosure in the Declarations section. If this is a breach of journal style and the Editor would prefer us to move this statement somewhere else in the manuscript, we will of course do so.

Justification is needed for why "thresholding sequencing reads by their average methylation level will reduce the effect of biological and technical variance."

A: We realise that this was not properly explained in the original manuscript. To make

Powered by Editorial Manager® and ProduXion Manager® from Aries Systems Corporation

the point clear in the revised manuscript, we have provided an extended explanation, replacing the previous sentence. It now reads as follows: “As number of events that lead to variance in methylation (base deamination, random single-base methylation events and sequencing errors) is limited at the level of individual reads (only a fraction of CpGs might be affected within the same read), the average methylation level of the read will be moderately affected by such events and can help distinguish hyper- from hypomethylated epialleles. We therefore hypothesized that thresholding sequence reads by their average methylation level will reduce the effect of biological and technical variance and facilitate the detection of infrequent hypermethylation events”. Notably, as we showed by sensitivity analyses, this hypothesis was correct.

Justification and motivation for the VEF metric is not provided, nor are all details provided on how it's calculated. I tried to follow the description for individual cytosines, but am immediately stuck on how to define Calpha for cytosine i. Calpha is described as the number of methylated cytosines in read pairs passing the threshold - it's not clear what is being counted over. Presumably this should only be considering reads that overlap that particular cytosine i, and perhaps it's counting over all cytosines in all such reads, but this is counting multiple times per cytosine (since more than 1 read can overlap a cytosine), so this seems to contradict the description. Moreover, if I just take the description at face value as "epialleles with similar methylation properties that is defined by thresholding", it's not clear what this represents biologically. It seems to intend to measure variability of methylation.

A: We understand that VEF is a newly introduced metric and that it should be explained in detail. Instead of providing more text, we have now included a panel B in Figure 1, where we graphically explain how VEF values are calculated at the level of individual bases. We believe that this figure panel, with its accompanying legend, will make the underlying calculations of VEF more easily accessible for the reader.
In addition, the extensive online documentation to which we point our users (at https://bbcg.github.io/epialleleR/articles/epialleleR.html or https://bioconductor.org/packages/devel/bioc/vignettes/epialleleR/inst/doc/epialleleR.html) provides further clarifications and scenarios for specific analyses.

It's not clear how VEF metrics compare to established measures of methylation variability and/or allelic variation of methylation (e.g. Variably Methylated Region identification, methHaplo, methtuple, DAMEfinder). There are alternatives discussed in terms of entropy and discordant reads, but they are not compared in the sensitivity analysis study of WBC.

A: Variably methylated regions (VMR), by definition, are the regions showing variable methylation levels between samples. Such regions are not what epialleleR is designed to quantify. Unlike VMR tools, epialleleR outputs per-base value (VEF) that can significantly boost sensitivity of aberrant or differentially methylated region detection tools for the detection of mosaic epimutations (epimutations present in 1 out of >100 cells). This is presented in the sensitivity analyses that employ the best to our knowledge DMR analysis tool (DMRcate).
We agree with the Reviewer that it is always a good practice to compare to established tools. However, the tools suggested by Reviewer (methHaplo, methtuple, DAMEfinder) actually have different purposes than epialleleR. They are used to assemble methylation haplotypes (methHaplo), detect changes in allele-specific methylation between two groups of samples (DAMEfinder), or count m-tuples in reads (methtuple). While they use BAM files as an input, none of them is able to output a metric which is directly comparable to VEF (per-CpG methylation level) or its purpose (to count concordant hypermethylation events, i.e., epimutations). For example, methHaplo specifically excludes CpGs with low methylation level (beta<0.1) as they are not informative for its purpose. In contrast, epialleleR is designed to detect and quantify rare epimutations, can extract all epialleles and can use optional VCF data to correlate SNVs with methylation – this was used by us to discover prenatal nature of BRCA1 epimutations that lead to cancer in adulthood. Besides, epialleleR can itself call methylation and use BAM files produced by virtually any aligners (which is not the case for tools mentioned above).
The other metrics (entropy, PDR, etc) are not per-base metrics and cannot be used for differential/aberrant methylation analysis.
As such, we feel it would leave the readers with a misleading impression about the

	tools and their purpose if we tried to present any comparison, and we have therefore not done this in the sensitivity analysis section.<br><br>A major limitation of the interpretation of so-called epialleles is the inability to distinguish allelic variation from cell type heterogeneity. This is acknowledged very briefly and very late in the manuscript. This needs to be stated up front. In addition, a discussion of potential remedies (if any) would be helpful.<br><br>A: We agree that cell type heterogeneity is a factor that must be taken into consideration in methylation analyses in general. But, while cell type heterogeneity can explain some of the methylation differences in other studies, the phenomenon we have described here does not depend on it. Only 1 CpG in this study and none of CpGs analysed in Lønning et al., 2022 have significantly different methylation levels between blood cell types. Regarding other regions, it is important to note that our method, epialleleR, is not itself biased by cell type heterogeneity because it is executed upstream of the data interpretation. epialleleR reports frequencies of individual epialleles or their groups (by methylation), while it is the downstream analysis (comparison of samples or their groups) which should account for any confounding factor, including, but not limited to, cell type heterogeneity. Given the possibility to perform allele-specific methylation calls with epialleleR, we actually believe it will be a useful addition to tools that may be used to address cell type heterogeneity, since methylation on different alleles in different tissues will indicate different methylation events to have occurred in different tissues of the same individual.<br><br>The simulated data are very unrealistic, which precludes interpretation of results. It seems that all CpGs were simulated with the same methylation level of 50%, and all other cyosines at 0.25% methylation levels. More importantly, introducing random sequencing errors is unrealistic, as per base sequence quality is known to be generally lower in read 2 compared to read 1. Further, there's a known increase in read 2 of M bias in bisulfite sequencing. With a more realistic error generating process, I expect choosing base with highest quality to be effectively the same as trimming read 2, meaning the results presented here are an artifact of simulation choices.<br><br>A: The purpose of the simulated data is to have a known level of methylation of all cytosines, so that the reported values can be compared to the expected ones. While the ground truth was chosen to be exactly 50% methylation in CG context and approximately 0.25% in CHG and CHH ones, this does not undermine the results of the comparison. We believe, the values applied are not relevant per se; they could have been 5% or 95% or anything else. The tools are still compared based on the lowest difference of observed (reported) values to the expected ones.<br>The Sherman bisulfite read simulator used by us here is a state-of-the-art tool, which uses the most realistic simulation strategy available. The errors are not entirely random but introduced according to exponential decay model (derived from real data). Simulated per-base quality scores follow the same curve as well. As pointed out by the Reviewer, it is indeed known that read 2 contains more base substitutions than read 1. However, the number of sequencing errors depends strongly on a sequence context and read length, therefore errors are present in both read 1 and read 2, and are more abundant at their ends. It is a quite common situation that a lower-quality 3' end of read 1 overlaps with higher-quality region of read 2 (either middle or its 5'). A simple trimming of a higher-quality part of read 2 will then result in lower-quality bases retained. This is why the base quality-driven merging will naturally result in fewer errors and we therefore consider it to be favourable.<br>The subtle increase in reporting accuracy is not very valuable on its own, but together with the vast speed improvement it allows us to recommend our method for virtually every analysis of methylation sequencing BAM files when methylation needs to be called at individual cytosine residues.<br><br>Methods are not described in enough detail to reproduce results. For example, the details on usage of Bismark in the accuracy simulation is not included (only for speed comparison).<br><br>A: The methylation reporting for accuracy comparison was done exactly as described for the speed comparison for all tools. For clarity, this has now been specified in the "Cytosine reporting accuracy comparison" paragraph in Materials and Methods of the

revised manuscript.

Owing to the major concerns listed thus far, I am not able to fully review the "Sensitivity analyses" section at this time.

A: We hope that the revisions in the manuscript text and the comments made here in our response letter, are clarifying and that all parts of the manuscript are now easily accessible for the reader.

Minor:

Organization of manuscript could use improvement (e.g. breaking up full page+ paragraphs, making sure headers accurately reflect section content).

A: We are uncertain of which specific paragraphs and/or subheadings the Reviewer would recommend to edit. Going through the manuscript, we have found that some of our previous subheadings were not fully precise covering the contents of the following paragraphs. Therefore, we have made some minor changes for better concordance. Thus, new subheadings in the revised manuscripts are “Reporting accuracy analyses” in the Results section and “Processing speed comparison” in the Material and Methods section. In addition, some very long paragraphs were broken into smaller paragraphs.

epialleleR is marked in Table 1 as not requiring a reference genome. But isn't it needed to obtain the bam file? If not, then how was the bam file generated for use with epiallelR? It's not a fair comparison to mark Bismark as needing a reference genome (to carry out the bam file generation step), as far as I'm aware this isn't used in the methylation extractor step in Bismark (the input is a BAM/SAM file).

A: Bismark methylation extractor indeed requires reference genome in addition to BAM input. For the methylation reporting, epialleleR does not require reference genome (similar to methylKit, but unlike methylation reporting by DRAGEN or Bismark).

In table 2, 'mean methylation level' is not generally considered an accuracy metric. Some metric of difference from ground truth should be shown.

A: We agree with the Reviewer that “mean methylation level” is indeed not an accuracy metric. Therefore, we provided “variance” as a measure of the differences between observed and expected values. Both these values allow the reader to see what tool reports closest to expected methylation values (0.5 and 0.0025 for cytosines in CG and CHG/CHH contexts, respectively). In order to clarify this issue, we have changed the title of the table, so that it now additionally includes these expected values.

When the authors state "Concordantly methylated alleles (genomic regions with spatially extended / haplotype-specific methylation)", I think they just mean allele-specific methylation. If not, please clarify. The other terms used (concordant methylation or spatially extended methylation) are nonstandard, and, puzzlingly, not mentioned in the citations provided for this sentence, where I searched for additional context.

A: By concordantly methylated alleles we mean reads with concordant methylation of cytosines (e.g., most of CpGs either methylated or unmethylated). We agree that this was unclear in our original manuscript and have now replaced the sentence with a simple explanation for “Concordantly methylated alleles” in the first sentence of the “Sensitivity analyses” paragraph of the Results section.

In Figure 2A what region is shown and how was this chosen? It appears the beta values are shown on a log scale, which is a nonstandard choice. M-values would be advisable to check as well.

A: Figure 2A shows all 138 CpGs included in the sensitivity analyses, with their genomic position as a categorical variable along x-axis. We understand that this was unclear and have now included an additional explanation in the figure legend.
Data are presented using log scale to illustrate exponential (3-fold) differences between cytosine methylation levels (1e-4 to 1e-2) between all samples. This would

	not be possible using a linear scale and we have therefore chosen to keep the log scale in the figure.<br>As epialleleR cytosine reports are similar to Bismark cytosine reports in that epialleleR outputs both numbers of methylated (“Ca” with thresholding or “C” without thresholding) and unmethylated (“T”) cytosines per genomic position, the package users can utilize those counts in any downstream analyses in the form of a beta value or M-value. Here, we chose to present beta/VEF values only, as sequencing data is compared with methylation array data, for which M-values are not available.<br><br>In Figure 2D/E, a measure of specificity should be reported as well.<br><br>A: The experimental setup allowed us to extract only the total number of real positive regions (P), the number of true positive regions (TP), and the number of false negative regions (FN=P–TP) for every analysis, while the numbers of true negative (TN) or false positive (FP) regions were undefined. Therefore, recall, or true positive rate (TPR=TP/P) was chosen as a sensitivity metric. This is described in Materials and Methods and the TPRs are now presented in the revised manuscript.<br><br><br>Should state much earlier in the manuscript which types of methylation sequencing data are suitable for this approach.<br><br>A: We agree that it would be beneficial to make an early statement about this and have now included this earlier in the text (Introduction and epialleleR implementation sections).<br>--
**Additional Information:**	
**Question**	**Response**
Are you submitting this manuscript to a special series or article collection?	No
**Experimental design and statistics**<br><br><br>Full details of the experimental design and statistical methods used should be given in the Methods section, as detailed in our Minimum Standards Reporting Checklist. Information essential to interpreting the data presented should be made available in the figure legends.<br><br><br>Have you included all the information requested in your manuscript?	Yes
**Resources**<br><br><br>A description of all resources used, including antibodies, cell lines, animals and software tools, with enough information to allow them to be uniquely identified, should be included in the Methods section. Authors are strongly encouraged to cite Research Resource	Yes

Identifiers (RRIDs) for antibodies, model organisms and tools, where possible.<br><br>Have you included the information requested as detailed in our Minimum Standards Reporting Checklist?	
**Availability of data and materials**<br><br>All datasets and code on which the conclusions of the paper rely must be either included in your submission or deposited in publicly available repositories (where available and ethically appropriate), referencing such data using a unique identifier in the references and in the "Availability of Data and Materials" section of your manuscript.<br><br>Have you have met the above requirement as detailed in our Minimum Standards Reporting Checklist?	Yes

Powered by Editorial Manager® and ProduXion Manager® from Aries Systems Corporation

Manuscript - with edits Click here to access/download;Manuscript;epialleleR-edits.pdf

epialleleR: an R/Bioconductor package for sensitive allele-specific methylation analysis in NGS data

Oleksii Nikolaienko[1,*], Per Eystein Lønning[1,2] and Stian Knappskog[1,2]

[1] K. G. Jebsen Center for Genome-Directed Cancer Therapy, Department of Clinical Science, University of Bergen, Bergen, 5021, Norway

[2] Department of Oncology, Haukeland University Hospital, Bergen, 5021, Norway

* To whom correspondence should be addressed. Tel: +47 559 76 444; Email: oleksii.nikolaienko@uib.no

Running title: Sensitive methylation analysis in NGS data

ABSTRACT

Low-level mosaic ~~methylation~~epimutations ~~of~~within the *BRCA1* gene promoter occurs in 5–8% of healthy individuals and is associated with a significantly elevated risk of breast and ovarian cancer. Similar events may also affect other tumour suppressor genes, potentially being a significant contributor to cancer burden. While this opens a new area for translational research, detection of low-level mosaic epigenetic events requires highly sensitive and robust methodology for methylation analysis. We here present epialleleR, a computational framework for sensitive detection, quantification and visualisation of mosaic epimutations in methylation sequencing data. Analysing simulated and real data sets, we provide in-depth assessments of epialleleR performance, and show that linkage to epihaplotype data is necessary to detect low-level methylation events. The epialleleR is freely available at https://github.com/BBCG/epialleleR and https://bioconductor.org/packages/epialleleR/ as an open source R/Bioconductor package.

KEYWORDS

Epigenetics, DNA methylation, somatic mosaicism, epigenetic mosaicism, methylation sequencing

INTRODUCTION

Cancer is a major health threat and cause of death worldwide. While the minority of cases are due to highly penetrant germline pathogenic variants (inherited cancers), the majority are considered sporadic cancers with no known germline genetic component.

In addition to genetic aberrations like single-nucleotide variants, indels, copy number alterations and rearrangements, cancers are known to harbour epigenetic disturbances, leading to aberrant transcriptional up- and downregulation. Epigenetic aberrations may arise during different stages of carcinogenesis as somatic epimutations (mirroring somatic mutations), or *in utero* (affecting several germline layers) as constitutional normal tissue epimutations. Several studies in large cohorts [1,2] have linked low-level mosaic (affecting a small subset of cells only) epimutations to breast and/or ovarian cancer risk. Research and interest in this field, however, have been limited by the fact that all these studies were conducted on patients already diagnosed with their cancers, questioning whether normal tissue methylation in these patients may be a cancer-initiating event or a secondary effect of the disease itself. Recently we found frequent (occurring in >5% of healthy women) though low-level (down to 0.03% of affected alleles) mosaic ~~methylation~~epimutations within the *BRCA1* gene promoter to be associated with a significantly elevated risk for subsequent high-grade ovarian as well as triple-negative breast cancer, in a large, population-based prospective cohort [3]. This finding raises a provoking question of whether similar low-level mosaic methylation events may affect other tumour suppressor genes, and be associated with an elevated risk of other cancer forms as well. While this opens a new research area related to cancer risk, there are technical issues to account for, as the low frequency of such mosaic epimutations limits the amplitude of observed changes in methylation. Thus, to explore such hypotheses, there is a need for robust and sensitive ~~methylation~~epimutation detection techniques.

Currently, the most widely used methods for DNA methylation profiling are BeadChip arrays (such as Illumina HumanMethylation450 or HumanMethylationEPIC) and a variety of methylation sequencing techniques (for details see [4]). These methods have different pros and cons: arrays allow

genome-wide assessment at a reduced cost, while the sequencing provides additional information on haplotype specificity of DNA methylation. The typical bioinformatic workflows designed to analyse both types of data usually result in sets of beta values (ratio of a count of methylated cytosines to the total sum of methylated and unmethylated bases) for each genomic position covered [5–7]. While this approach is suitable for addressing large differences in DNA methylation profiles between two sets of samples (e.g., cases and controls), it lacks sensitivity for low-level mosaic ~~methylation~~epimutation detection, as the detection is hindered by sometimes much more common biological variation [8,9] or technical artefacts [10,11]. Moreover, the lack of haplotype linkage makes such analysis difficult in BeadChip array-based data sets, and therefore requires nontrivial approaches [12]. In contrast, analysis of NGS-based data can provide much higher sensitivity when the base-resolution methylation data is combined with information on allelic belongingness (epihaplotype linkage).

Here, we present a computational framework for sensitive detection and quantification of low-frequency, mosaic epimutations in methylation sequencing data. The provided methods can be used for the discovery of low-frequency epialleles connected to disease risk [3], as well as for purposes allowing less sensitivity, such as assessments related to treatment response [13,14], or to the development of treatment resistance [15]. Importantly, the framework also allows to connect DNA methylation status with potential underlying cis-factors, such as single-nucleotide variations or mutations within the immediate proximity.

The versatility of the framework makes it applicable for analysis of data from any methylation sequencing experiment, given that methylation in these data can be called at individual cytosine residues. Both single-end and paired-end sequencing alignment files can be used as an input, and in cases where~~n~~ methylation calls are not available, this framework allows to call cytosine methylation and permanently store calls in a binary sequence alignment/map (BAM) file.

RESULTS

epialleleR implementation

The presence of hypermethylated *BRCA1* alleles (epimutations) in normal tissue (WBC) has been shown *qualitatively* for 5–8% of adult women [1]. However, the associated *quantitative* changes in DNA methylation at the level of individual CpGs are typically small (in most cases the intraindividual frequency of epimutations is between 0.03–1% [3]), and therefore indistinguishable from the background methylation level due to inherent biological (potentially spurious single-base methylation events) and technical (sequencing errors) variance [8]. Methylation statuses of neighbouring CpGs are often concordant [16], and such spatially extended epigenetic changes are often associated with a gene expression silencing [17]. Given the potential biological (gene inactivation) and clinical (cancer risk) importance of ~~hypermethylated epialleles~~epimutations, we focused on quantification of hypermethylation events that span over several CpGs~~, i.e. detection of epihaplotypes~~, accounting for both methylation status of individual CpGs within the sequence read as well as the average methylation level of the sequence read itself. This is possible in NGS-based data sets, while it is not in array-based data where methylation information of different CpGs cannot be connected to each other as in haplotype data.

As number of events that lead to variance in methylation (base deamination, random single-base methylation events and sequencing errors) is limited at the level of individual reads (only a fraction of CpGs might be affected within the same read), the average methylation level of the read will be moderately affected by such events and can help distinguish hyper- from hypomethylated epialleles. We ~~also~~therefore hypothesized that thresholding sequence reads by their average methylation level will reduce the effect of biological and technical variance and facilitate the detection of ~~low-frequency~~infrequent hypermethylation events. Despite the trivial nature of the task and an increasing demand for such analyses, no suitable generic solution was publicly available. We therefore implemented a solution using R software environment for statistical computing [18], a de facto standard for scientific data analysis. The implemented solution, epialleleR, loads methylation call strings and short sequence reads from supplied binary sequence alignment/map (BAM) file, optionally thresholds read pairs according to their methylation properties, and produces methylation

reports for individual cytosines as well as genomic regions of interest (Fig. 1A). During BAM loading, pairs of sequence reads and corresponding methylation call strings are merged according to Phred quality score values (i.e., base with the highest score is chosen) to preserve information of the highest quality. The optional thresholding defines a subpopulation of epialleles of interest and is based on the minimum number and the average methylation level of cytosines in various sequence contexts (e.g., CpG, CHG, or CHH). The thresholding parameters are fully adjustable to target desired population of epialleles; their default values (minimum 2 CpG sites, minimum average methylation beta value of 0.5 for CpG sites, maximum average methylation beta value of 0.1 for non-CpG sites) performed well in the study linking mosaic BRCA1 epimutations and cancer risk [3] and were used here in all downstream analyses.

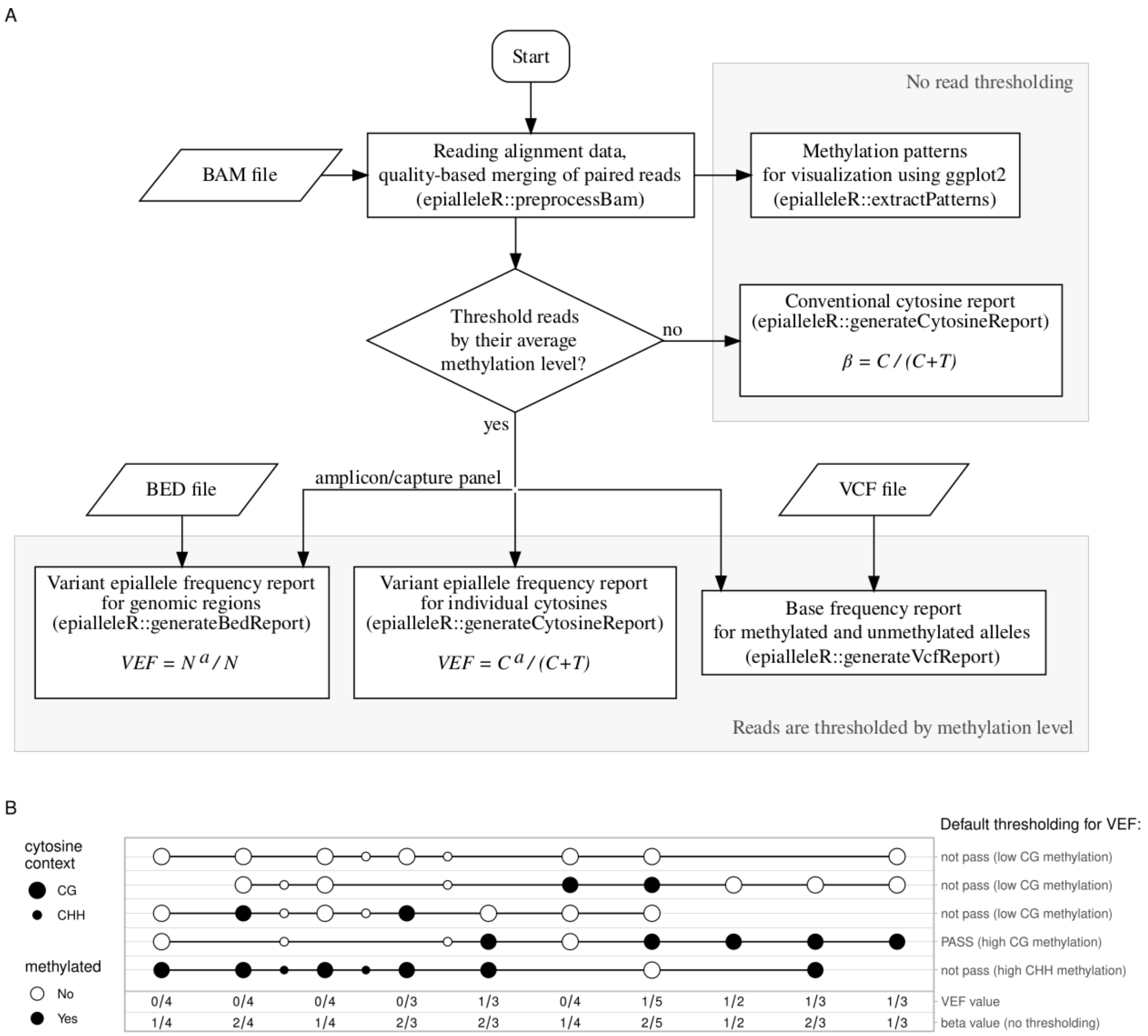

Figure 1. (A) Flowchart of epialleleR package data processing steps. The formulas using to calculate conventional beta as well as VEF values are given in boxes. C and T, total number of cytosines and thymines at particular genomic position, respectively; C^a, number of cytosines at particular genomic position ~~in~~within read pairs passing a particular methylation threshold ($C^a \leq C$); N, total number of read pairs, mapped to a particular genomic region; N^a, number of mapped read pairs, passing a particular methylation threshold ($N^a \leq N$). (B) Schematic illustration of cytosine methylation (circles) within epialleles (horizontal lines), and results of thresholding by average read methylation level (labels on the right) using default parameters (i.e., at least 2 CpGs in CG context, at least 50% methylation within CG context, at most 10% methylation outside of CG context). Resulting per-cytosine beta and VEF values are given under each CpG (large circles). In the context of a typical CpG-rich regulatory region, the three hypomethylated epialleles on the top represent typically abundant scattered methylation or sequencing artifacts, the epiallele at the bottom represents the product of incomplete bisulfite conversion, while second epiallele from the bottom represents a true biologically relevant epimutation that leads to gene silencing.

The optional thresholding of sequence reads defines two modes of epialleleR function. Without thresholding, epialleleR produces conventional cytosine reports similar to the ones produced

by other tools (e.g., Bismark [5]). In this case, methylation beta value for every genomic location is computed as a ratio of a number of methylated cytosines to total number of methylated and unmethylated cytosines: $\beta = C / (C+T)$.

When read thresholding is performed (default mode of action), the level of methylation per every genomic position, denoted as a Variant Epiallele Frequency (VEF), is calculated as a ratio of a number of methylated cytosines in read pairs passing the threshold (C^a) to total number of methylated and unmethylated cytosines in all read pairs: $VEF = C^a / (C+T)$. (see Fig. 1B for an example). When the report is prepared at a level of extended genomic regions rather than individual bases, VEF equals to the ratio of a number of read pairs passing threshold (N^a) to the total number of read pairs (N) overlapping the region of interest: $VEF = N^a / N$. The term "Variant Epiallele" here represents a group of epialleles (i.e., individual methylation patterns) with similar methylation properties that is defined by thresholding, therefore VEF effectively represents the frequency of this group of epialleles passing the threshold at the level of individual cytosines or extended genomic regions.

Methylation beta values (from conventional reporting) as well as VEF values (from default reporting mode with read thresholding) can be produced from any number of BAM files with no prior hypothesis, as long as experimental setup allows to call methylation on per-base level. Both of these values effectively represent methylation levels per genomic position and, as such, can be directly used further as an input for other bioinformatic tools including, but not limited to, differential methylation analysis tools.

If methylation statuses of cytosines were not determined, epialleleR allows to create and store methylation calls, allowing analysis of BAM files created by various methylation sequencing alignment tools.

When optional data on single-nucleotide variantions is provided, epialleleR quantifies the balance or skewness of methylation between alleles, thereby enablingaiding in assessment discovery of potential allele specificity of epimutations.

To provide a comprehensive range of means for epiallele analysis, the package also offers methods allowing visualisation and characterisation of *all* individual epialleles (methylation patterns) in a sample (see Fig. 1 and Supplementary Figs for details). If required, extracted patterns can include other, non-cytosine bases of interest (e.g., single-nucleotide variations), which allows to connect methylation properties of epialleles with sequence features in proximity. During methylation pattern extraction, every epiallele is characterised by number of context sites and methylation level (average beta value) and is assigned with a unique identifier (Fowler-Noll-Vo FNV-1a non-cryptographic hash [19]) that solely depends on positions of included cytosine (and other optional) bases and their methylation states (or nucleotide symbols for optional bases), enabling not only to group epialleles by their methylation properties but also reliably and consistently track individual epialleles of high importance across different samples or even studies. The average beta values for all extracted patterns as well as patterns themselves can be explored to optimise thresholding parameters for a genomic region of interest.

Increasing scale and depth of methylation sequencing experiments impose a requirement on the speed of data processing. Therefore, all time-consuming subtasks were implemented using optimised C/C++ subroutines and, whenever possible, linked to HTSlib, unified C library for high-throughput sequencing data processing [20]. The R package epialleleR is freely available at the Bioconductor package repository (http://bioconductor.org/packages/epialleleR/).

~~A~~Reporting accuracy analyses

First, we sought to validate the accuracy of methylation reporting by epialleleR in its conventional mode (no read thresholding) as compared with three other commonly used tools for which read thresholding is not available: Bismark [5], methylKit [21] and Illumina DRAGEN Bio-IT Platform. For this purpose, we simulated large sets of paired-end bisulfite sequencing reads (2x151bp, 100 million read pairs covering human chromosome 19). In contrast to real datasets, simulated data allows to calculate “ground truth” methylation levels for unbiased comparison. Simulation parameters were selected to obtain exact methylation levels of 50% for cytosines in CpG context (n=2211240) and

methylation level of approximately 0.25% (bisulfite conversion rate of ~99.75%) for cytosines in CHG and CHH contexts (n=6593900 and 19210572, respectively). In addition to endogenous deamination events [8], bisulfite treatment-induced changes [10] and variation in conversion rates [22], sequencing itself can introduce errors that vary in range depending on assay type and sequencing technology [11]. Therefore, we introduced variable level of artificial sequencing errors (0%, 0.1%, 0.3% or 0.6%) and evaluated their effect on the accuracy of reported methylation metrics, applying a selected set of methods (for comparison see Table 1). Analysis on exactly the same task (BAM file to cytosine report) revealed that reported values were close to their theoretical expectations for all methods, with epialleleR being the least affected by sequencing errors, i.e., maintaining the smallest deviance of reported versus expected methylation beta values for all samples with sequencing errors introduced, possibly owing to read quality-assisted merging of paired reads (Table 2, further details in Supplementary table 1).

Table 1. Selected characteristics of software/hardware solutions for methylation reporting.

method	requires reference (genomic) sequence	removes overlaps within read pairs	outputs epiallele frequencies	processing speed, read pairs per second
Bismark	yes	yes (trims read2)	no	40–2,800
methylKit	no	yes (trims read2)	no	9,900–15,400
DRAGEN	yes	yes (trims read2)	no	2,000–183,000
epialleleR	no	yes (base with the highest quality is chosen)	yes	129,000–231,000

Table 2. Selected accuracy metrics (average beta values and their variance) of methylation reporting. Average reported beta values that are closest to the expected beta values (0.0025 for cytosines in CHG/CHH contexts and 0.5 for cytosines in CG context) and lowest variance values are shown in bold.

sequencing error rate	method	CHH		CHG		CpG	
		mean	variance	mean	variance	mean	variance
0.00%	DRAGEN	**0.002501**	1.28E-05	**0.002500**	1.27E-05	0.499997	5.96E-07
	Bismark	0.002501	1.34E-05	0.002500	1.33E-05	**0.499997**	**5.92E-07**
	methylKit	0.002503	**1.28E-05**	0.002501	**1.27E-05**	0.499997	5.97E-07

	epialleleR	**0.002501**	1.28E-05	**0.002500**	1.27E-05	0.499997	5.96E-07
0.10%	DRAGEN	0.002661	1.37E-05	0.002662	1.36E-05	0.499835	1.73E-06
	Bismark	0.002646	1.42E-05	0.002648	1.41E-05	0.499850	1.69E-06
	methylKit	0.002662	1.37E-05	0.002664	1.36E-05	0.499835	1.75E-06
	epialleleR	**0.002624**	**1.35E-05**	**0.002626**	**1.34E-05**	**0.499872**	**1.54E-06**
0.30%	DRAGEN	0.002977	1.53E-05	0.002980	1.53E-05	0.499504	3.22E-06
	Bismark	0.002928	1.57E-05	0.002931	1.57E-05	0.499554	3.03E-06
	methylKit	0.002978	1.52E-05	0.002982	1.52E-05	0.499506	3.21E-06
	epialleleR	**0.002857**	**1.47E-05**	**0.002860**	**1.47E-05**	**0.499628**	**2.59E-06**
0.60%	DRAGEN	0.003498	1.79E-05	0.003506	1.78E-05	0.498942	1.29E-05
	Bismark	0.003393	1.81E-05	0.003402	1.81E-05	0.499051	1.25E-05
	methylKit	0.003497	1.78E-05	0.003501	1.78E-05	0.498952	1.28E-05
	epialleleR	**0.003237**	**1.66E-05**	**0.003241**	**1.65E-05**	**0.499222**	**1.14E-05**

Sensitivity analyses

Concordantly methylated alleles (~~genomic regions with spatially extended / haplotype-specific methylation~~alleles with most of their CpGs methylated) are thought to possess high biological importance [3,15]. Spontaneous 5-methyl cytosine (5mC) deamination, sequencing errors, as well as genuine single-nucleotide methylation/demethylation events affect observed background methylation level and can therefore hinder the detection of low-frequency hyper- or hypomethylated alleles. Differences in experimental conditions provide an additional level of variability which can sometimes be tackled by normalisation during postprocessing [23]. In contrast to the DNA methylation analysis using BeadChip arrays (such as Illumina HumanMethylation450 and HumanMethylationEPIC) which report average methylation values at the level of individual cytosines only, next-generation sequencing provides an additional data dimension by linking methylation levels of individual nucleotides within a genomic region covered by a sequencing read (epihaplotypes). However, this information is lost when methylation is assessed and reported without accounting for its allelic distribution. To evaluate the sensitivity of detection for low-frequency monoallelic hypermethylation

events in next-generation sequencing data, we simulated an extended set of samples using real, amplicon-based bisulfite sequencing data for human WBC (n=10 with almost no hypermethylated alleles, as described in Materials and Methods) and fully methylated control DNA samples. Combining real WBC DNA bisulfite sequencing data allowed to introduce sample-to-sample variability although maintaining biologically relevant background methylation levels across sequenced regions, while admixing fully methylated reads simulated low-frequency, concordant methylation events. The amplicons used, covered promoter regions of the tumour suppressors *MLH1*, *CDKN2A*, *MGMT*, *CDH1*, and *BRCA1*. The distributions of per-read beta values (Supplementary Fig. 1) and methylation patterns (Supplementary Fig. 2) of admixed samples show the expected abundance of hypermethylated (average $\beta \geq 0.5$) alleles and confirm their high similarity to the real samples (Supplementary Figs 3 and 4). Conventional cytosine reports (no read thresholding) as well as VEF reports (with read thresholding) were prepared and used for unsupervised clustering of samples and differentially methylated region (DMR) discovery. Despite quite low overall methylation level of amplified regions (average beta value of 0.014, median of 0.005; Fig. 2A), t-SNE analysis based on beta values was not able to discriminate between samples with 0.01%, 0.03%, 0.10%, 0.30% of methylated reads, or no methylated reads added (Fig. 2B, left panel). On the other hand, VEF value-based t-SNE analysis resulted in spatially well-separated clusters that corresponded to each level of admixed methylated reads (Fig. 2B, right panel). Intergroup DMR discovery based on beta values (Fig. 2C, left panel) showed fewer number of regions found as well as higher associated false discovery rate (FDR), while discovery based on VEF values resulted in all five possible regions identified for all possible intergroup comparisons as well as generally lower associated FDR. When each sample with admixed methylated reads was compared against the group of samples without admixed methylated reads, recall metrics for differential (by DMRcate [24]; Fig. 2D) or aberrant (by ramr [12]; Fig. 2E) methylation analysis were notably higher for analyses based on VEF values (Fig. 2D–E, right panels) in comparison with analyses based on beta values (Fig. 2D–E, left panels). This

shows that VEF values are more valuable for detection and analysis of low-frequency (≤1%) hypermethylation events than methylation beta values.

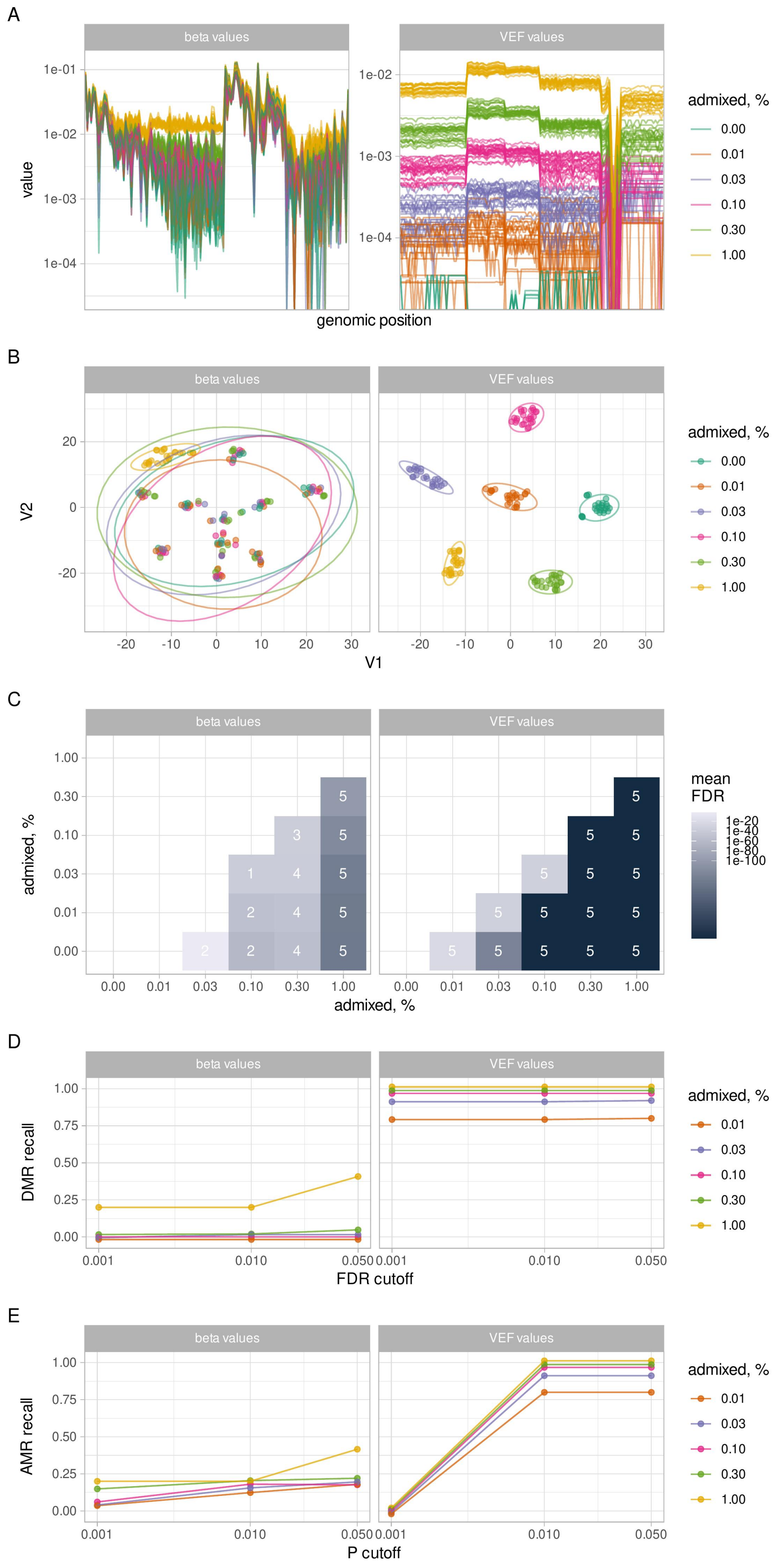
A
beta values
VEF values
value
1e-01
1e-02
1e-03
1e-04
genomic position
admixed, %
0.00
0.01
0.03
0.10
0.30
1.00
B
beta values
VEF values
V2
V1
20
0
-20
-20
-10
0
10
20
30
admixed, %
0.00
0.01
0.03
0.10
0.30
1.00
C
beta values
VEF values
admixed, %
mean
FDR
1e-20
1e-40
1e-60
1e-80
1e-100
D
beta values
VEF values
DMR recall
FDR cutoff
1.00
0.75
0.50
0.25
0.00
0.001
0.010
0.050
admixed, %
0.01
0.03
0.10
0.30
1.00
E
beta values
VEF values
AMR recall
P cutoff
1.00
0.75
0.50
0.25
0.00
0.001
0.010
0.050
admixed, %
0.01
0.03
0.10
0.30
1.00

Figure 2. (A) Line plots of beta (left panel) and VEF (right panel) values for individual samples, colour-coded and grouped according to the amount of admixed methylated reads. Each line represents a sample; y-axis, methylation value of all CpGs (n=138) sorted by their genomic position (categorical x-axis). (B) Embedding plots for t-SNE analysis using beta (left panel) and VEF (right panel) values. Ellipses represent 95% confidence levels. (C) Heatmap of mean false discovery rate for differentially methylated regions (DMRs) identified by DMRcate. Labels indicate the number of DMRs found (of total of five regions possible). (D) Recall rate for DMR identification using DMRcate for varying FDR cutoffs. (E) Recall rate for aberrantly methylated regions identification using ramr for varying p value cutoffs.

BeadChip arrays, such as Illumina HumanMethylationEPIC, is another widely used, amplification-free method to assess genome-wide DNA methylation for a reduced cost. In order to directly compare the sensitivities of targeted NGS and of the BeadChip arrays for the detection of low-frequency DNA methylation events, we employed both of the methods to analyse small set of samples (n=8) carrying low-frequency methylation in at least one of the assayed regions (promoter regions of *MLH1*, *CDKN2A*, *MGMT*, *CDH1*, and *BRCA1*). Sample distributions of per-read beta values (Supplementary Fig. 3) and methylation patterns (Supplementary Fig. 4) show that these samples indeed contain varying frequencies of hypermethylated (average $\beta \geq 0.5$) alleles. For unbiased comparison, we limited the corresponding data sets to the CpGs assayed and sufficiently covered by both techniques. Analysis revealed that VEF values of samples with many hypermethylated alleles (e.g., A26 and A45 for *BRCA1*; as apparent from Fig. 3A) differ significantly (Fig. 3B) from VEF values of samples with only a few or no hypermethylated alleles (e.g., A02 or A05 for *BRCA1*; Fig. 3A). When VEF values were used for identification of aberrantly or differentially methylated regions by ramr [12] or DMRcate [24], respectively, the significant regions found correlated well with the notable presence of hypermethylated alleles. Of note, slightly inferior performance of DMRcate is probably due to the fact that for some of the genomic regions too many samples in this subset simultaneously contained hypermethylated epialleles. When DMRcate was used for the same purpose on an extended set of sequenced samples (n=18, containing n=10 samples characterised by the absence of hypermethylated alleles that were used to create admixed sample set), its performance in identification of hypermethylated epiallele-containing samples was higher (Supplementary Fig. 5).

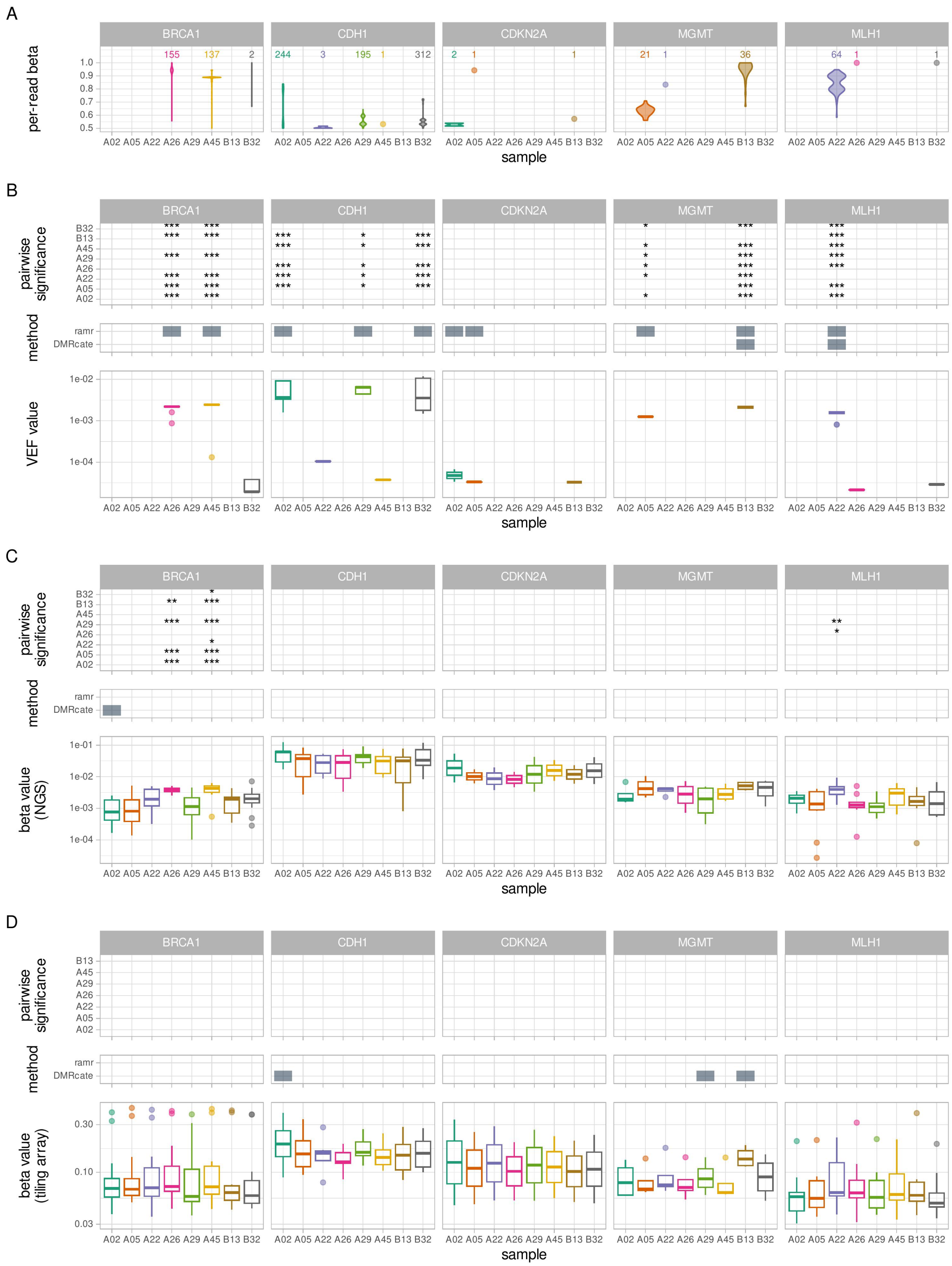

Figure 3. (A) Distribution of per-read beta values for NGS read pairs covering CpGs that are common for NGS and BeadChip array. For clarity, only the reads with average beta of at least 0.5 (i.e., representing hypermethylated epialleles) are included. Single observations are shown as dots, number of observations is given above. Complete density plots are provided

in the Supplementary Fig. 3. Corresponding methylation patterns are provided in the Supplementary Fig. 4. (B) Lower panel: box plots of NGS-derived VEF values for individual CpGs; middle panel: significant aberrantly or differentially methylated regions identified by ramr or DMRcate, respectively, based on VEF values; upper panel: significance levels from pairwise comparison of VEF values. (C) Lower panel: box plots of NGS-derived beta values for individual CpGs; middle panel: significant aberrantly or differentially methylated regions identified by ramr or DMRcate, respectively, based on NGS-derived beta values; upper panel: significance levels from pairwise comparison of NGS-derived beta values. (D) Lower panel: box plots of BeadChip array-derived beta values for individual CpGs; middle panel: significant aberrantly or differentially methylated regions identified by ramr or DMRcate, respectively, based on BeadChip array-derived beta values; upper panel: significance levels from pairwise comparison of BeadChip array-derived beta values. (B–D) The lower and upper hinges of boxes correspond to the first (Q_1) and third (Q_3) quartiles; the bar in the middle correspond to the median value; the upper and lower whisker extend to Q_3+1.5*IQR and Q_1-1.5*IQR, respectively, while the values outside this range (outliers) are plotted as dots. Zero values are not plotted. *** $p<0.001$, ** $p<0.01$, * $p<0.05$, blank $p\geq0.05$.

In contrast, only a few significant differences remained when NGS beta values were used for sample comparison (Fig. 3C), while pairwise comparisons based on BeadChip array beta values did not reveal any significant differences between samples (Fig. 3D). The search for aberrantly or differentially methylated regions using either NGS or array beta values did not result in identification of such regions in relevant (according to methylation patterns or beta value densities) samples. Generally higher beta values of BeadChip array as compared to NGS beta values likely mask subtle changes in methylation caused by the presence of infrequent hypermethylated alleles and hinder the detection of differences between samples.

Several scores to describe and quantify variability in DNA methylation in sequencing reads (within-sample heterogeneity, WSH) have been proposed [25]. In order to assess WSH, we evaluated the difference in combinatorial entropy between each pair of samples using methclone [26] (Supplementary Fig. 6A). The largest (by absolute value) reported difference in combinatorial entropy of -2.59 between any pair of samples confirms a high similarity between sample methylation profiles, of note, being much smaller than cutoffs for epiallele shifts between samples analysed in [25] (-60 and lower). Further, we also calculated four additional heterogeneity scores: combinatorial entropy, epipolymorphism, fraction of discordant read pairs (FDRP) and proportion of discordant reads (PDR). The scores themselves (Supplementary Fig. 6B) and the levels of score-based pairwise significance

between samples (Supplementary Fig. 6C), are not generally consistent with fractions of hypermethylated (average β≥0.5) alleles (Fig. 3A and Supplementary Fig. 3) or VEF values (Fig. 3B): e.g., samples A26 and A45 have a notable fraction of hypermethylated reads in *BRCA1* promoter region compared to other samples, although it is not reflected at the level of WSH scores. Importantly, WSH scores produced cannot be directly used as an input for DMR analysis tools, which are commonly employed to characterise exact differences in methylation between samples.

It is known that DNA methylation profiles of blood samples depend on the varying contribution of individual blood cell types [27,28]. While we cannot exclude that hypermethylated alleles present in the samples analysed here originate from a particular blood cell type, low-level, mosaic ~~methylation~~epimutations of at least *BRCA1* ~~was~~were previously shown to be independent of blood subfraction composition [1]. Of note, only one CpG (cg05785947 in *CDH1*) out of 37 used in NGS vs BeadChip array comparison here, was found to be significantly differentially methylated between blood cell types of healthy males; and none of CpGs were significantly differentially methylated between blood cell types of newborns.

Processing speed analyses

Methylation sequencing data produced by contemporary techniques varies in scale and depth and may contain several thousands to billions of single or paired-end reads. To analyse them efficiently, computational methods must be scalable and fast enough for as large as possible range of sample counts or data file sizes. Unfortunately, many academic tools use computationally complex algorithms that do not scale to contemporary tasks. We compared data processing speed for epialleleR versus methylKit, Bismark, and DRAGEN Bio-IT Platform, performing exactly the same task (BAM file to cytosine report) of methylation reporting across input data coming from various assays: amplicon-based (n=10 samples with a depth of coverage of ~20,000x), genome-wide capture-based (n=10 with a depth of coverage of ~60x and n=3 with a depth of coverage of ~1000x) or whole-genome bisulfite sequencing (WGBS, n=6 with a depth of coverage of ~60x). The obtained results confirm very

efficient implementation of epialleleR and its suitability for analysis of data sets of any depth and coverage (Fig. 4, Table 1).

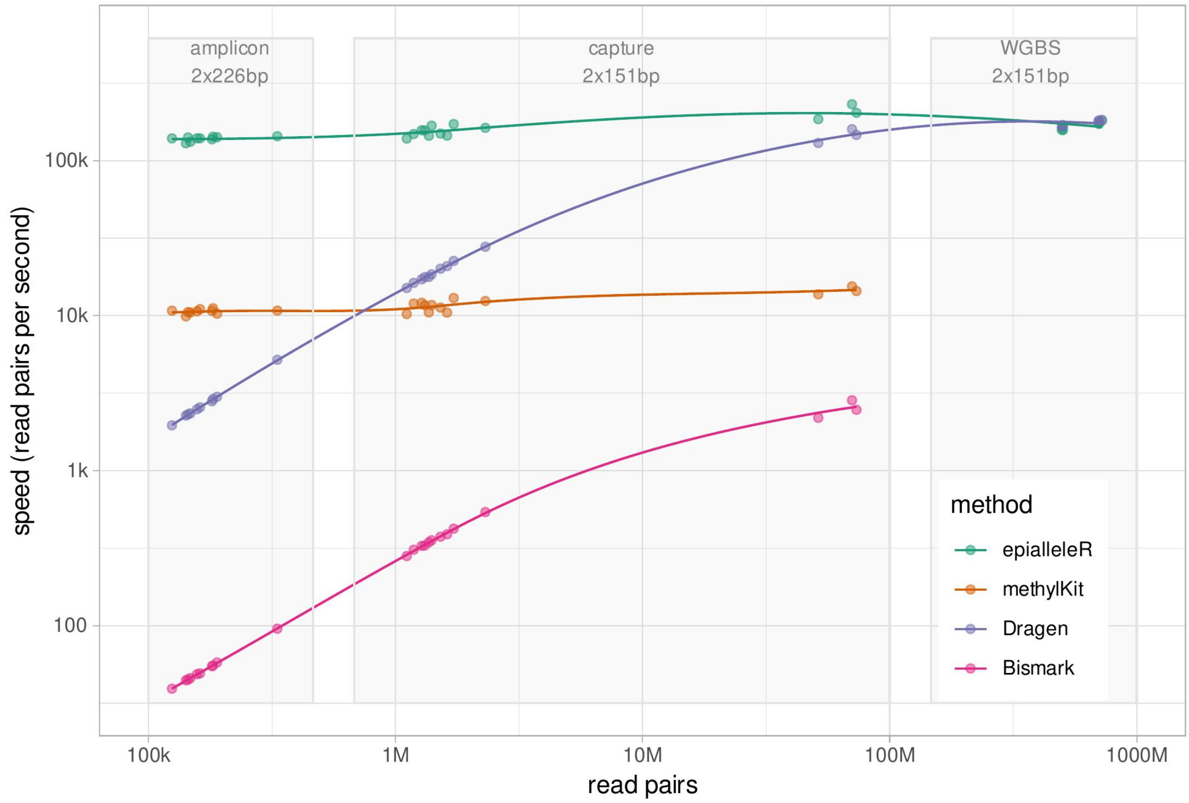

Figure 4. Data processing speed (in read pairs per second) of epialleleR as compared to three other methods for methylation reporting (methylKit, Bismark, and DRAGEN Bio-IT Platform). Read count (in number of pairs) is given at x-axis; light grey boxes outline data obtained by targeted amplicon-based, genome-wide capture-based, or whole-genome bisulfite sequencing.

DISCUSSION

While conflicting data has linked low-level mosaic primary constitutional epimutations to cancer risk for more than a decade [29], we have recently obtained firm evidence implicating primary epimutations within the *BRCA1* gene in an elevated risk of incident breast and ovarian cancer [3]. The assumption that such epimutations may affect other tumour suppressor genes and, therefore, lead to

other cancer forms [29], institutes a new research area with respect to cancer risk. Further, the findings of such epimutations in umbilical cord blood [1] indicate prenatal events of a yet unknown genesis. This creates the need for multidisciplinary studies on the mechanisms of these events and on their effects in respect to cancer risk, as well as the need for ultrasensitive methods allowing sample assessment at a high scale.

Here, we present the details on a fast, accurate, and sensitive method to detect, quantify and visualise epialleles in NGS data. The method shows its superiority versus conventional methods of methylation reporting, especially when applied for detection of low-frequency methylation events, as it is by design less susceptible to variations in conversion efficiency or sequencing quality. Although epialleleR is not a differential methylation analysis tool, its output can be directly used to group samples based on their methylation profiles (by applying a simple threshold or using unsupervised clustering), as well as an input for other differential/aberrant methylation analysis software (the latter is not possible for WSH analysis tools).

We thoroughly tested epialleleR using bisulfite sequencing data; the method, however, can also be applied to analyse and compare data obtained using any methylation sequencing technique (reduced representation bisulfite sequencing, RRBS; oxidative bisulfite sequencing, oxBS-Seq; Tet-assistant bisulfite sequencing, TAB-Seq), as long as methylation in these data can be called at individual cytosine residues instead of being analysed by comparing relative abundance of the fragments (such as for methylation sensitive restriction enzyme sequencing, MRE-Seq, or methylated DNA immunoprecipitation sequencing, MeDIP-Seq).

~~S~~The possibility to call cytosine methylation for alignment files created by different short sequence aligners, subtle though noticeable changes in cytosine reporting accuracy together with immense speed gain make epialleleR a method of choice not only for discovery of infrequent hypermethylated epialleles (as in [3]), but also as a tool to produce conventional (no read thresholding) cytosine reports from any methylation sequencing alignment files.

–The implemented method is fully documented and can be easily used from within the R environment for statistical computing. With the epialleleR already revealing its suitability for detection of low-level mosaic methylation events in a large data set [3], we believe it constitutes an optimal tool for assessment of low-level mosaic ~~methylation~~epimutations with respect to risk of cancer as well as other diseases of relevance.

CONCLUSIONS

Here, we present epialleleR, very fast, accurate, and sensitive method to detect, quantify and visualise epialleles in NGS data. Efficient implementation and improvements in cytosine reporting accuracy allow us to recommend epialleleR not only for analysis of methylation patterns and to enhance low-level differentially methylated region discovery, but also as a conventional cytosine reporting tool for various kinds of methylation sequencing data. The epialleleR R/Bioconductor package is freely available at https://bioconductor.org/packages/epialleleR/ and https://github.com/BBCG/epialleleR.

MATERIALS AND METHODS

Next-generation sequencing

White blood cells (WBC) DNA samples from anonymized males (n=88) [30,31] and human HCT116 DKO methylated DNA control sample (Zymo Research, cat.no. D5014-2) were bisulfite converted, and five DNA fragments, representing promoter regions of five established tumour suppressor genes, were amplified using custom set of primers (GRCh38 assembly coordinates of assayed regions: *MLH1*, chr3:36993123–36993500; *CDKN2A*, chr9:21974554–21974921; *MGMT*, chr10:129467118–129467477; *CDH1*, chr16:68737102–68737469; *BRCA1*, chr17:43125171–43125550), indexed, and sequenced similarly to as previously described [3] (GSE201688). The resulting average coverage was 5000x–50000x per amplicon.

Bioinformatic and statistical analyses

Massive parallel sequencing (NGS) reads were mapped/aligned to the GRCh38 human reference genome and the methylation was called using Illumina DRAGEN Bio-IT Platform (v3.9.5) with the following parameters: --methylation-mapping-implementation single-pass, --enable-methylation-calling true, --methylation-generate-cytosine-report false, --methylation-protocol non-directional, --enable-sort false, unless stated otherwise. R software environment for statistical computing (v4.1.2) was used for all downstream statistical analyses.

The frequency of hypermethylated alleles across assayed regions in n=88 male WBC DNA samples were estimated using epialleleR::generateAmpliconReport with the following parameters: min.mapq=30, min.baseq=20, nthreads=4, threshold.reads=TRUE, report.context="CG", and bed.file pointing to a location of BED (browser extensible data) file with genomic regions amplified (see amplicon coordinates above). Two sample subgroups (n=8 and n=10) were selected for sensitivity analyses based on the frequencies of hypermethylated alleles as explained below.

Cytosine reporting accuracy comparison

Four sets of paired-end sequencing reads (151bp, 50 million read pairs each set) were simulated using Sherman Bisulfite FastQ Read Simulator (RRID:SCR_001294) [32] with the following options: --length 151, --number_of_seqs 50000000, --paired_end, --minfrag 70, --maxfrag 400, --CG_conversion 0, --CH_conversion 99.5 and varying sequencing error rate (--error_rate parameter) of 0%, 0.1%, 0.3% or 0.6%. Human chromosome 19 sequence (GRCh38.p13 NC_000019.10, 58617616 bp, 1105620 forward strand CpGs) was used as a reference genome for read simulation and mapping/alignment due to its highest CpG content across all human chromosomes [33] and in order to maintain optimal balance of analysis speed and base coverage. Each set of reads was then duplicated, all read1 cytosines (C) and read2 guanines (G) in any context in the duplicate sets were replaced with thymines (T) and adenines (A), respectively. Then, duplicate sets (i.e., unmethylated reads) were merged with original sets (i.e., methylated reads) resulting in four sets of reads 100 million pairs each, with the cytosine conversion rate of exactly 50% and about 99.75% in CpG and non-CpG contexts, respectively.

The mapping and alignment of simulated reads was performed using Illumina DRAGEN Bio-IT Platform v3.9.5 with the following modification in parameters: --methylation-protocol directional. Methylation reporting by all tools was done as described below (reporting parameters in Speed comparison section).

Sensitivity comparison on admixed samples

In order to simulate variable methylation levels while maintaining biological heterogeneity of the samples, we selected ten male DNA NGS samples with the lowest frequency of hypermethylated alleles across all assayed regions, then admixed varying fractions of reads from two random samples and additionally "spiked" certain number of fully methylated reads from methylated DNA control sample. This resulted in 150 samples containing 0%, 0.01%, 0.03%, 0.1%, 0.3% or 1% of methylated reads per sample (25 samples per every category).

Read mapping, alignment, methylation calling, and generation of genome-wide cytosine reports was performed using Illumina DRAGEN Bio-IT Platform as described above. VEF calling was performed using epialleleR::generateCytosineReport with the following parameters: min.mapq=0, min.baseq=0, nthreads=4, threshold.reads=TRUE, report.context="CG".

Methylation patterns and per-read beta values for all samples were extracted using epialleleR::extractPatterns with the following parameters: min.mapq=30, min.baseq=20, nthreads=4, clip.patterns=FALSE, and bed.file pointing to a location of BED file with genomic regions amplified (see amplicon coordinates above).

Barnes-Hut t-Distributed Stochastic Neighbor Embedding (t-SNE) analysis was performed using R package Rtsne v0.15 [34] and matrices of beta or VEF values for all genomic positions of CpGs with the coverage of at least 1000x and available values for all analysed samples (total number of CpGs, n=138; *MLH1*, n=20; *CDKN2A*, n=35; *MGMT*, n=33; *CDH1*, n=32; *BRCA1*, n=18).

Sensitivity comparison to methylation array data

Eight additional WBC DNA NGS samples from anonymized males carrying hypermethylated alleles in at least one of the assayed regions were selected, and VEF calling was performed using epialleleR::generateCytosineReport with the following parameters: min.mapq=30, min.baseq=20, nthreads=4, threshold.reads=TRUE, report.context="CG". The same DNA samples were also bisulfite converted using the Zymo EZ DNA Methylation Kit (Zymo Research, cat.no. D5001), and genome-wide methylation levels were assessed using Illumina HumanMethylationEPIC BeadChip arrays according to manufacturer's instructions. Resulting IDAT files were processed (normalized and annotated) with the minfi Bioconductor package [23] using the preprocessQuantile method with outlier thresholding enabled (GSE201689). For direct comparison, only the CpGs that are covered in all samples by both BeadChip arrays (p-value of 0) and targeted sequencing (minimum sequencing coverage of 5000x) were retained (*MLH1*, n=10; *CDKN2A*, n=2; *MGMT*, n=4; *CDH1*, n=7; *BRCA1*, n=14). Pairwise sample comparison was performed using t-test with Holm adjustment for multiple comparisons.

The sets of CpGs that are differentially methylated between cell blood types were reported previously: DNA methylation profiles for six blood cell types from six males [27,35], and DNA methylation profiles for seven blood cell types from cord blood of 104 newborns [28,36]. CpG-level differential methylation analysis p values were Holm-adjusted and the ones remained significant (adjusted $p \leq 0.05$; n=73629 of total 456655 for male blood data set; n=221246 of total 429794 for newborn cord blood data set) were checked for overlap with the set of CpGs analysed in this study (n=35 CpGs of total n=37 were present in each of male/newborn data sets).

Differential methylation analysis

Differentially methylated regions (DMRs) were called using R package DMRcate (v2.12.0) with the following parameters: lambda=1000, min.cpgs=2, pcutoff="fdr" [24]. Aberrantly methylated regions were called using R package ramr (v1.6.0) with the following parameters: ramr.method="beta", min.cpgs=2, merge.window=500 [12]. To enable maximum likelihood estimation of beta distribution parameters, all zeros were replaced with minimum double values (2.26e-308).

For intergroup DMR discovery in admixed samples, pairwise comparison of sample groups defined by the amount of admixed reads was performed (n=25 samples in each group) using the default level of false discovery rate (FDR) cutoff (equals 0.05). For DMR discovery in real samples, as DMRcate methods require two classes/categories for comparison, every real sample from the test dataset was tested against all the other samples using the default FDR cutoff value.

To assess DMR (or AMR) recall metrics in admixed samples, every sample with admixed reads was compared using DMRcate (or ramr) to the group of 25 samples without admixed reads at a varying level of FDR (or p value) cutoff of 0.05, 0.01, or 0.001. As the admixed reads covered all five assayed regions, only the total number of real positive regions (P, equals 5 for each comparison), the number of true positive regions (TP), and the number of false negative regions (FN=P–TP) were known, while the numbers of true negative (TN) or false positive (FP) regions were undefined. Therefore, recall, or true positive rate (TPR=TP/P) was chosen as a sensitivity metric.

Within-sample heterogeneity

Estimation of within-sample heterogeneity (WSH) was performed on eight samples used in the sensitivity comparison between array- and NGS-based methylation profiling. Difference in entropy was evaluated using methclone (v1) [26] with a distance cutoff of 500 and minimum read coverage of 1000 for every pair of samples. As methclone outputs values for multiple genomic regions, the minimum value (representing absolute largest difference) was selected and used further. Entropy, epipolymorphism, fraction of discordant read pairs (FDRP) and proportion of discordant reads (PDR) were evaluated using R package WSH (v0.1.6) [25] with the following options: mapq.filter=30, window.size=500, and bam.file pointing to a location of BAM file. Due to exponential complexity of FDRP calculation, option max.reads was set to 100 for FDRP calculation and to 1e+06 otherwise. Pairwise sample score comparison was performed using t-test with Holm adjustment for multiple comparisons.

~~S~~Processing speed comparison

Comparison of processing speed was performed on 29 BAM files containing paired-end alignments and methylation calls derived from bisulfite sequencing of human WBC DNA samples prepared using the following assays: A) amplicon-based sequencing of promoter regions of *BRCA1* gene (n=10 files, 0.12–0.33 million read pairs per file, average coverage of ~20,000x) [3]; B) genome-wide capture-based bisulfite sequencing of promoter regions of 283 tumour suppressor genes (n=10 files, 1.11–2.31 million read pairs per file, average coverage of ~60x; and n=3 files, 51.4–73.4 million read pairs per file, average coverage of ~1000x) [37,38]; C) whole-genome bisulfite sequencing (n=6 files, 497–723 million read pairs per file, average coverage of ~60x; epialleleR and Illumina DRAGEN Bio-IT Platform only). The two former data sets (A and B) were generated in-house and described previously, while the latter data (C) were obtained from NCBI Sequence Read Archive (GEO/SRA samples GSM3683953/SRX6640720, GSM3683958/SRX6640725, GSM3683965/SRX6640732, GSM3683951/SRX6640718, GSM3683955/SRX6640722, and GSM3683962/SRX6640729) and reported elsewhere [39].

Processing times to produce conventional cytosine reports were recorded as following:
Bismark CX methylation reports were created using Bismark v0.22.3 (RRID:SCR_005604) [5] with the following parameters: command bismark_methylation_extractor, --paired-end, --no_overlap, --comprehensive, --gzip, --mbias_off --parallel 8, --cytosine_report, --CX, --buffer_size 64G. As parallel processing was requested, Bismark used up to 24 cores for some of its subtasks.

methylKit CX methylation reports were created using R/Bioconductor package methylKit v1.20.0 (RRID:SCR_005177) [21] with the following parameters: function methylKit::processBismarkAln, minqual=0, mincov=0, save.context=c("CpG","CHG","CHH"), nolap=TRUE and location pointing to the location of a BAM file. Parallel processing is currently not available for methylKit::processBismarkAln.

epialleleR CX methylation reports were created using R/Bioconductor package epialleleR v1.3.5 with the following parameters: function epialleleR::generateCytosineReport, min.mapq=0, min.baseq=0, nthreads=4 (number of HTSlib decompression threads), threshold.reads=FALSE,

report.context="CX" and bam pointing to the location of a BAM file. epialleleR methods currently run in a single-threaded mode only but can benefit from additional BAM decompression threads provided by HTSlib.

Illumina DRAGEN is a hardware solution that relies on the presence of FPGA accelerator card, which precludes DRAGEN software execution on other platforms. At the same time, outdated software development tools available at DRAGEN (GCC v4.8.5, R v3.6.0) impede installation of third-party software and R/Bioconductor packages and may potentially affect their performance. Therefore, testing of methylation reporting tools was carried out in two different settings.

Bismark, methylKit and epialleleR were tested on the workstation equipped with AMD EPYC 7742 64-core processor, 512GB of memory and the Red Hat Enterprise Linux Server release 7.9 (Developer Toolset 6, GCC v6.3.1), with BAM files retrieved from high-speed (10Gbps) network accessible storage.

DRAGEN CX methylation reports were created using Illumina DRAGEN Bio-IT Platform v3.9.5 (Intel Xeon Gold 6126 48-core processor, 256GB of memory and CentOS Linux release 7.5.1804) with the following parameters: --methylation-generate-cytosine-reports=true, --enable-sort=false, --enable-duplicate-marking=false, --methylation-report-only=true and --bam-input pointing to the location of a BAM file. Default number of threads (up to 24) were used for data processing using DRAGEN; BAM files were accessed from local, high-speed NVMe solid state disk.

For Bismark and DRAGEN, elapsed time measurements were stably reproducible, thus processing time was recorded only once for each file. For methylKit and epialleleR, the tests were run five times in sequential random order by means of R package microbenchmark v1.4.9, and the average time was used in comparison to mitigate variability in processing time measurements.

DECLARATIONS

Ethics approval and consent to participate

Ethics approvals and other relevant information for patient-generated data used in speed assessment were included and described in previous studies [3,30,37,38]. All analyses of biomaterial were approved by Regional Ethics Committees for medical research and all samples were collected after written informed consent from the sample donors (REK-vest Norway reference numbers: 3.2008.1932, 2015/1493 and 2018/1566.

Availability of data and materials

The epialleleR R/Bioconductor package (biotools:epialleleR, RRID:SCR_023913) is freely available at https://bioconductor.org/packages/epialleleR/ and https://github.com/BBCG/epialleleR. The R scripts used in this manuscript and the data underlying accuracy and sensitivity analyses are freely available at DataverseNO (https://doi.org/10.18710/2BQTJP). Sensitive data used for the processing speed assessment are available from the authors in accordance with study protocols.

Public data for sensitivity analysis have been deposited at NCBI Gene Expression Omnibus under accession number GSE201690. Public whole-genome bisulfite sequencing data used for the processing speed assessment are available at NCBI Sequencing Read Archive under accession number SRP217135.

Supplementary Data are available online.

Previous use of epialleleR

A previous version of this manuscript was deposited in bioRxiv (doi: 10.1101/2022.06.30.498213) and the epialleleR method has been applied in [3].

Competing interests

P.E.L. has for other projects received research funding from AstraZeneca, Novartis, Pfizer, and Illumina, and honoraria through speaker's bureaux from AstraZeneca, Pierre-Fabre, Roche, AbbVie

and Akademikonferens. He has participated in advisory boards for AstraZeneca, Laboratorios and Farmaceuticos Rovi. S.K. has received research funding for other projects from AstraZeneca, Pfizer, and Illumina, and speaker's bureaux honoraria from AstraZeneca, Pfizer, Novartis, and Pierre Fabre.

Funding

This work was supported by the K.G.Jebsen foundation [grant number SKGJ-MED-020 to P.E.L.]; The Norwegian Cancer Society [grant number 190281-2017 to S.K.]; and The Norwegian Research Council [grant number 617344-1 to P.E.L.]. Funding for open access charge: The Norwegian Research Council.

Authors contribution

Conceived the project: O.N., P.E.L., S.K. Supervised the project: P.E.L., S.K. Conceived, designed, and implemented the software and the analysis pipeline: O.N. Wrote the paper: O.N., P.E.L., S.K. All authors read and approved the final manuscript.

REFERENCES

1. Lønning PE, Berge EO, Bjørnslett M, Minsaas L, Chrisanthar R, Høberg-Vetti H, et al.. White Blood Cell BRCA1 Promoter Methylation Status and Ovarian Cancer Risk. *Ann Intern Med*. American College of Physicians; 2018; doi: 10.7326/M17-0101.

2. Prajzendanc K, Domagała P, Hybiak J, Ryś J, Huzarski T, Szwiec M, et al.. BRCA1 promoter methylation in peripheral blood is associated with the risk of triple-negative breast cancer. *International Journal of Cancer*. 2020; doi: https://doi.org/10.1002/ijc.32655.

3. Lønning PE, Nikolaienko O, Pan K, Kurian AW, Eikesdal HP, Pettinger M, et al.. Constitutional BRCA1 Methylation and Risk of Incident Triple-Negative Breast Cancer and High-grade Serous Ovarian Cancer. *JAMA Oncol*. 2022; doi: 10.1001/jamaoncol.2022.3846.

4. Sun R, Zhu P. Advances in measuring DNA methylation. *Blood Sci*. 2022; doi: 10.1097/BS9.0000000000000098.

5. Krueger F, Andrews SR. Bismark: a flexible aligner and methylation caller for Bisulfite-Seq applications. *Bioinformatics*. 2011; doi: 10.1093/bioinformatics/btr167.

6. Maksimovic J, Phipson B, Oshlack A. A cross-package Bioconductor workflow for analysing methylation array data. *F1000Res*. 2016; doi: 10.12688/f1000research.8839.3.

7. Fortin J-P, Triche TJ Jr, Hansen KD. Preprocessing, normalization and integration of the Illumina HumanMethylationEPIC array with minfi. *Bioinformatics*. 2017; doi: 10.1093/bioinformatics/btw691.

8. Youk J, An Y, Park S, Lee J-K, Ju YS. The genome-wide landscape of C:G > T:A polymorphism at the CpG contexts in the human population. *BMC Genomics*. 2020; doi: 10.1186/s12864-020-6674-1.

9. Gu J, Stevens M, Xing X, Li D, Zhang B, Payton JE, et al.. Mapping of Variable DNA Methylation Across Multiple Cell Types Defines a Dynamic Regulatory Landscape of the Human Genome. *G3 (Bethesda)*. 2016; doi: 10.1534/g3.115.025437.

10. Kint S, Spiegelaere WD, Kesel JD, Vandekerckhove L, Criekinge WV. Evaluation of bisulfite kits for DNA methylation profiling in terms of DNA fragmentation and DNA recovery using digital PCR. *PLOS ONE*. Public Library of Science; 2018; doi: 10.1371/journal.pone.0199091.

11. Stoler N, Nekrutenko A. Sequencing error profiles of Illumina sequencing instruments. *NAR Genomics and Bioinformatics*. 2021; doi: 10.1093/nargab/lqab019.

12. Nikolaienko O, Lønning PE, Knappskog S. ramr: an R/Bioconductor package for detection of rare aberrantly methylated regions. *Bioinformatics*. 2021; doi: 10.1093/bioinformatics/btab586.

13. Kondrashova O, Topp M, Nesic K, Lieschke E, Ho G-Y, Harrell MI, et al.. Methylation of all BRCA1 copies predicts response to the PARP inhibitor rucaparib in ovarian carcinoma. *Nat Commun*. 2018; doi: 10.1038/s41467-018-05564-z.

14. Nesic K, Kondrashova O, Hurley RM, McGehee CD, Vandenberg CJ, Ho G-Y, et al.. Acquired RAD51C Promoter Methylation Loss Causes PARP Inhibitor Resistance in High-Grade Serous Ovarian Carcinoma. *Cancer Res*. 2021; doi: 10.1158/0008-5472.CAN-21-0774.

15. Hurley RM, McGehee CD, Nesic K, Correia C, Weiskittel TM, Kelly RL, et al.. Characterization of a RAD51C-silenced high-grade serous ovarian cancer model during development of PARP inhibitor resistance. *NAR Cancer*. 2021; doi: 10.1093/narcan/zcab028.

16. Huh I, Wu X, Park T, Yi SV. Detecting differential DNA methylation from sequencing of bisulfite converted DNA of diverse species. *Briefings in Bioinformatics*. 2019; doi: 10.1093/bib/bbx077.

17. Anastasiadi D, Esteve-Codina A, Piferrer F. Consistent inverse correlation between DNA methylation of the first intron and gene expression across tissues and species. *Epigenetics & Chromatin*. 2018; doi: 10.1186/s13072-018-0205-1.

18. R Core Team. R: A Language and Environment for Statistical Computing. Vienna, Austria: R Foundation for Statistical Computing;

19. Fowler G, Noll LC, Vo K-P, Eastlake 3rd DE, Hansen T. The FNV Non-Cryptographic Hash Algorithm. Internet Engineering Task Force; Report No.: draft-eastlake-fnv-17.

20. Bonfield JK, Marshall J, Danecek P, Li H, Ohan V, Whitwham A, et al.. HTSlib: C library for reading/writing high-throughput sequencing data. *GigaScience*. 2021; doi: 10.1093/gigascience/giab007.

21. Akalin A, Kormaksson M, Li S, Garrett-Bakelman FE, Figueroa ME, Melnick A, et al.. methylKit: a comprehensive R package for the analysis of genome-wide DNA methylation profiles. *Genome Biol*. 2012; doi: 10.1186/gb-2012-13-10-r87.

22. Sun Z, Vaisvila R, Hussong L-M, Yan B, Baum C, Saleh L, et al.. Nondestructive enzymatic deamination enables single-molecule long-read amplicon sequencing for the determination of 5-methylcytosine and 5-hydroxymethylcytosine at single-base resolution. *Genome Res*. 2021; doi: 10.1101/gr.265306.120.

23. Aryee MJ, Jaffe AE, Corrada-Bravo H, Ladd-Acosta C, Feinberg AP, Hansen KD, et al.. Minfi: a flexible and comprehensive Bioconductor package for the analysis of Infinium DNA methylation microarrays. *Bioinformatics*. 2014; doi: 10.1093/bioinformatics/btu049.

24. Peters TJ, Buckley MJ, Chen Y, Smyth GK, Goodnow CC, Clark SJ. Calling differentially methylated regions from whole genome bisulphite sequencing with DMRcate. *Nucleic Acids Res*. 2021; doi: 10.1093/nar/gkab637.

25. Scherer M, Nebel A, Franke A, Walter J, Lengauer T, Bock C, et al.. Quantitative comparison of within-sample heterogeneity scores for DNA methylation data. *Nucleic Acids Res*. 2020; doi: 10.1093/nar/gkaa120.

26. Li S, Garrett-Bakelman F, Perl AE, Luger SM, Zhang C, To BL, et al.. Dynamic evolution of clonal epialleles revealed by methclone. *Genome Biol*. 2014; doi: 10.1186/s13059-014-0472-5.

27. Reinius LE, Acevedo N, Joerink M, Pershagen G, Dahlén S-E, Greco D, et al.. Differential DNA methylation in purified human blood cells: implications for cell lineage and studies on disease susceptibility. *PLoS One*. 2012; doi: 10.1371/journal.pone.0041361.

28. Bakulski KM, Feinberg JI, Andrews SV, Yang J, Brown S, L McKenney S, et al.. DNA methylation of cord blood cell types: Applications for mixed cell birth studies. *Epigenetics*. 2016; doi: 10.1080/15592294.2016.1161875.

29. Lønning PE, Eikesdal HP, Løes IM, Knappskog S. Constitutional Mosaic Epimutations – a hidden cause of cancer? *Cell Stress*. Shared Science Publishers; 2019; doi: 10.15698/cst2019.04.183.

30. Knappskog S, Bjørnslett M, Myklebust LM, Huijts PEA, Vreeswijk MP, Edvardsen H, et al.. The MDM2 promoter SNP285C/309G haplotype diminishes Sp1 transcription factor binding and reduces risk for breast and ovarian cancer in Caucasians. *Cancer Cell*. 2011; doi: 10.1016/j.ccr.2010.12.019.

31. Knappskog S, Gansmo LB, Romundstad P, Bjørnslett M, Trovik J, Sommerfelt-Pettersen J, et al.. MDM2 promoter SNP344T>A (rs1196333) status does not affect cancer risk. *PLoS One*. 2012; doi: 10.1371/journal.pone.0036263.

32. Krueger F. Sherman - bisulfite-treated Read FastQ Simulator.

33. Harris RA, Raveendran M, Worley KC, Rogers J. Unusual sequence characteristics of human chromosome 19 are conserved across 11 nonhuman primates. *BMC Evol Biol*. 2020; doi: 10.1186/s12862-020-1595-9.

34. Krijthe JH. Rtsne: T-Distributed Stochastic Neighbor Embedding using Barnes-Hut Implementation.

35. Jaffe AE. FlowSorted.Blood.450k: Illumina HumanMethylation data on sorted blood cell populations.

36. Andrews SV, Bakulski KM. FlowSorted.CordBlood.450k: Illumina 450k data on sorted cord blood cells.

37. Poduval DB, Ognedal E, Sichmanova Z, Valen E, Iversen GT, Minsaas L, et al.. Assessment of tumor suppressor promoter methylation in healthy individuals. *Clin Epigenetics*. 2020; doi: 10.1186/s13148-020-00920-7.

38. Eikesdal HP, Yndestad S, Elzawahry A, Llop-Guevara A, Gilje B, Blix ES, et al.. Olaparib monotherapy as primary treatment in unselected triple negative breast cancer☆. *Annals of Oncology*. 2021; doi: 10.1016/j.annonc.2020.11.009.

39. Zhou L, Ng HK, Drautz-Moses DI, Schuster SC, Beck S, Kim C, et al.. Systematic evaluation of library preparation methods and sequencing platforms for high-throughput whole genome bisulfite sequencing. *Sci Rep*. 2019; doi: 10.1038/s41598-019-46875-5.

Manuscript - clean version Click here to access/download;Manuscript;epialleleR-clean.pdf

epialleleR: an R/Bioconductor package for sensitive allele-specific methylation analysis in NGS data

Oleksii Nikolaienko[1,*], Per Eystein Lønning[1,2] and Stian Knappskog[1,2]

[1] K. G. Jebsen Center for Genome-Directed Cancer Therapy, Department of Clinical Science, University of Bergen, Bergen, 5021, Norway

[2] Department of Oncology, Haukeland University Hospital, Bergen, 5021, Norway

* To whom correspondence should be addressed. Tel: +47 559 76 444; Email: oleksii.nikolaienko@uib.no

Running title: Sensitive methylation analysis in NGS data

ABSTRACT

Low-level mosaic epimutations within the *BRCA1* gene promoter occurs in 5–8% of healthy individuals and is associated with a significantly elevated risk of breast and ovarian cancer. Similar events may also affect other tumour suppressor genes, potentially being a significant contributor to cancer burden. While this opens a new area for translational research, detection of low-level mosaic epigenetic events requires highly sensitive and robust methodology for methylation analysis. We here present epialleleR, a computational framework for sensitive detection, quantification and visualisation of mosaic epimutations in methylation sequencing data. Analysing simulated and real data sets, we provide in-depth assessments of epialleleR performance, and show that linkage to epihaplotype data is necessary to detect low-level methylation events. The epialleleR is freely available at https://github.com/BBCG/epialleleR and https://bioconductor.org/packages/epialleleR/ as an open source R/Bioconductor package.

KEYWORDS

Epigenetics, DNA methylation, somatic mosaicism, epigenetic mosaicism, methylation sequencing

INTRODUCTION

Cancer is a major health threat and cause of death worldwide. While the minority of cases are due to highly penetrant germline pathogenic variants (inherited cancers), the majority are considered sporadic cancers with no known germline genetic component.

In addition to genetic aberrations like single-nucleotide variants, indels, copy number alterations and rearrangements, cancers are known to harbour epigenetic disturbances, leading to aberrant transcriptional up- and downregulation. Epigenetic aberrations may arise during different stages of carcinogenesis as somatic epimutations (mirroring somatic mutations), or *in utero* (affecting several germline layers) as constitutional normal tissue epimutations. Several studies in large cohorts [1,2] have linked low-level mosaic (affecting a small subset of cells only) epimutations to breast and/or ovarian cancer risk. Research and interest in this field, however, have been limited by the fact that all these studies were conducted on patients already diagnosed with their cancers, questioning whether normal tissue methylation in these patients may be a cancer-initiating event or a secondary effect of the disease itself. Recently we found frequent (occurring in >5% of healthy women) though low-level (down to 0.03% of affected alleles) mosaic epimutations within the *BRCA1* gene promoter to be associated with a significantly elevated risk for subsequent high-grade ovarian as well as triple-negative breast cancer, in a large, population-based prospective cohort [3]. This finding raises a provoking question of whether similar low-level mosaic methylation events may affect other tumour suppressor genes, and be associated with an elevated risk of other cancer forms as well. While this opens a new research area related to cancer risk, there are technical issues to account for, as the low frequency of such mosaic epimutations limits the amplitude of observed changes in methylation. Thus, to explore such hypotheses, there is a need for robust and sensitive epimutation detection techniques.

Currently, the most widely used methods for DNA methylation profiling are BeadChip arrays (such as Illumina HumanMethylation450 or HumanMethylationEPIC) and a variety of methylation sequencing techniques (for details see [4]). These methods have different pros and cons: arrays allow

genome-wide assessment at a reduced cost, while the sequencing provides additional information on haplotype specificity of DNA methylation. The typical bioinformatic workflows designed to analyse both types of data usually result in sets of beta values (ratio of a count of methylated cytosines to the total sum of methylated and unmethylated bases) for each genomic position covered [5–7]. While this approach is suitable for addressing large differences in DNA methylation profiles between two sets of samples (e.g., cases and controls), it lacks sensitivity for low-level mosaic epimutation detection, as the detection is hindered by sometimes much more common biological variation [8,9] or technical artefacts [10,11]. Moreover, the lack of haplotype linkage makes such analysis difficult in BeadChip array-based data sets, and therefore requires nontrivial approaches [12]. In contrast, analysis of NGS-based data can provide much higher sensitivity when the base-resolution methylation data is combined with information on allelic belongingness (epihaplotype linkage).

Here, we present a computational framework for sensitive detection and quantification of low-frequency, mosaic epimutations in methylation sequencing data. The provided methods can be used for the discovery of low-frequency epialleles connected to disease risk [3], as well as for purposes allowing less sensitivity, such as assessments related to treatment response [13,14], or to the development of treatment resistance [15]. Importantly, the framework also allows to connect DNA methylation status with potential underlying cis-factors, such as single-nucleotide variations or mutations within the immediate proximity.

The versatility of the framework makes it applicable for analysis of data from any methylation sequencing experiment, given that methylation in these data can be called at individual cytosine residues. Both single-end and paired-end sequencing alignment files can be used as an input, and in cases where methylation calls are not available, this framework allows to call cytosine methylation and permanently store calls in a binary sequence alignment/map (BAM) file.

RESULTS

epialleleR implementation

The presence of hypermethylated *BRCA1* alleles (epimutations) in normal tissue (WBC) has been shown *qualitatively* for 5–8% of adult women [1]. However, the associated *quantitative* changes in DNA methylation at the level of individual CpGs are typically small (in most cases the intraindividual frequency of epimutations is between 0.03–1% [3]), and therefore indistinguishable from the background methylation level due to inherent biological (potentially spurious single-base methylation events) and technical (sequencing errors) variance [8]. Methylation statuses of neighbouring CpGs are often concordant [16], and such spatially extended epigenetic changes are often associated with a gene expression silencing [17]. Given the potential biological (gene inactivation) and clinical (cancer risk) importance of epimutations, we focused on quantification of hypermethylation events that span over several CpGs, accounting for both methylation status of individual CpGs within the sequence read as well as the average methylation level of the sequence read itself. This is possible in NGS-based data sets, while it is not in array-based data where methylation information of different CpGs cannot be connected to each other as in haplotype data.

As number of events that lead to variance in methylation (base deamination, random single-base methylation events and sequencing errors) is limited at the level of individual reads (only a fraction of CpGs might be affected within the same read), the average methylation level of the read will be moderately affected by such events and can help distinguish hyper- from hypomethylated epialleles. We therefore hypothesized that thresholding sequence reads by their average methylation level will reduce the effect of biological and technical variance and facilitate the detection of infrequent hypermethylation events. Despite the trivial nature of the task and an increasing demand for such analyses, no suitable generic solution was publicly available. We therefore implemented a solution using R software environment for statistical computing [18], a de facto standard for scientific data analysis. The implemented solution, epialleleR, loads methylation call strings and short sequence reads from supplied binary sequence alignment/map (BAM) file, optionally thresholds read pairs according to their methylation properties, and produces methylation reports for individual cytosines as well as genomic regions of interest (Fig. 1A). During BAM loading, pairs of sequence reads and

corresponding methylation call strings are merged according to Phred quality score values (i.e., base with the highest score is chosen) to preserve information of the highest quality. The optional thresholding defines a subpopulation of epialleles of interest and is based on the minimum number and the average methylation level of cytosines in various sequence contexts (e.g., CpG, CHG, or CHH). The thresholding parameters are fully adjustable to target desired population of epialleles; their default values (minimum 2 CpG sites, minimum average methylation beta value of 0.5 for CpG sites, maximum average methylation beta value of 0.1 for non-CpG sites) performed well in the study linking mosaic BRCA1 epimutations and cancer risk [3] and were used here in all downstream analyses.

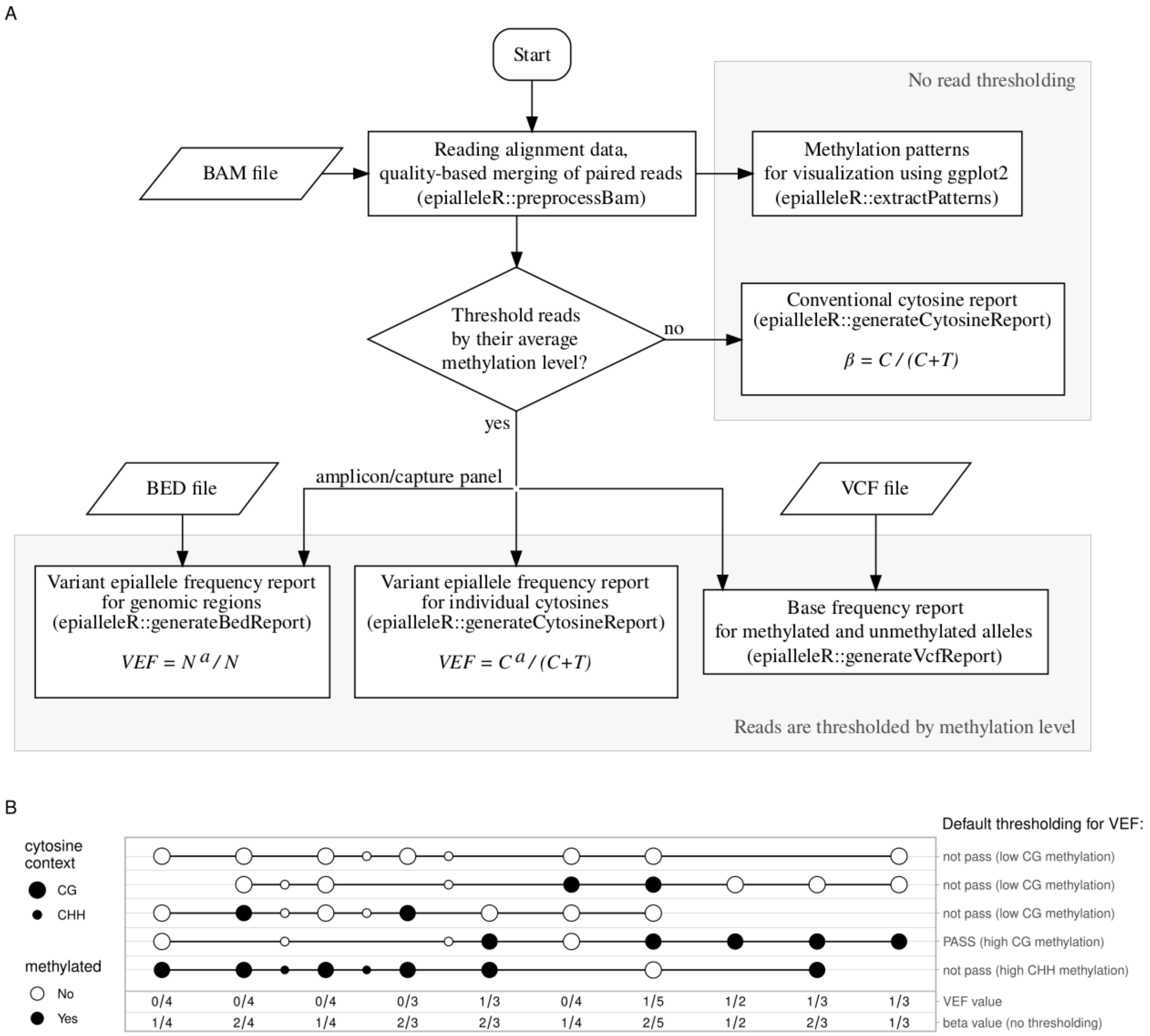

Figure 1. (A) Flowchart of epialleleR package data processing steps. The formulas using to calculate conventional beta as well as VEF values are given in boxes. C and T, total number of cytosines and thymines at particular genomic position, respectively; C^a, number of cytosines at particular genomic position within read pairs passing a particular methylation threshold ($C^a \leq C$); N, total number of read pairs, mapped to a particular genomic region; N^a, number of mapped read pairs, passing a particular methylation threshold ($N^a \leq N$). (B) Schematic illustration of cytosine methylation (circles) within epialleles (horizontal lines), and results of thresholding by average read methylation level (labels on the right) using default parameters (i.e., at least 2 CpGs in CG context, at least 50% methylation within CG context, at most 10% methylation outside of CG context). Resulting per-cytosine beta and VEF values are given under each CpG (large circles). In the context of a typical CpG-rich regulatory region, the three hypomethylated epialleles on the top represent typically abundant scattered methylation or sequencing artifacts, the epiallele at the bottom represents the product of incomplete bisulfite conversion, while second epiallele from the bottom represents a true biologically relevant epimutation that leads to gene silencing.

The optional thresholding of sequence reads defines two modes of epialleleR function. Without thresholding, epialleleR produces conventional cytosine reports similar to the ones produced

by other tools (e.g., Bismark [5]). In this case, methylation beta value for every genomic location is computed as a ratio of a number of methylated cytosines to total number of methylated and unmethylated cytosines: $\beta = C / (C+T)$.

When read thresholding is performed (default mode of action), the level of methylation per every genomic position, denoted as a Variant Epiallele Frequency (VEF), is calculated as a ratio of a number of methylated cytosines in read pairs passing the threshold (C^a) to total number of methylated and unmethylated cytosines in all read pairs: $VEF = C^a / (C+T)$ (see Fig. 1B for an example). When the report is prepared at a level of extended genomic regions rather than individual bases, VEF equals to the ratio of a number of read pairs passing threshold (N^a) to the total number of read pairs (N) overlapping the region of interest: $VEF = N^a / N$. The term “Variant Epiallele” here represents a group of epialleles (i.e., individual methylation patterns) with similar methylation properties that is defined by thresholding, therefore VEF effectively represents the frequency of this group of epialleles passing the threshold at the level of individual cytosines or extended genomic regions.

Methylation beta values (from conventional reporting) as well as VEF values (from default reporting mode with read thresholding) can be produced from any number of BAM files with no prior hypothesis, as long as experimental setup allows to call methylation on per-base level. Both of these values effectively represent methylation levels per genomic position and, as such, can be directly used further as an input for other bioinformatic tools including, but not limited to, differential methylation analysis tools.

If methylation statuses of cytosines were not determined, epialleleR allows to create and store methylation calls, allowing analysis of BAM files created by various methylation sequencing alignment tools.

When optional data on single-nucleotide variants is provided, epialleleR quantifies the balance or skewness of methylation between alleles, thereby enabling assessment of potential allele specificity of epimutations.

To provide a comprehensive range of means for epiallele analysis, the package also offers methods allowing visualisation and characterisation of *all* individual epialleles (methylation patterns) in a sample (see Fig. 1 and Supplementary Figs for details). If required, extracted patterns can include other, non-cytosine bases of interest (e.g., single-nucleotide variations), which allows to connect methylation properties of epialleles with sequence features in proximity. During methylation pattern extraction, every epiallele is characterised by number of context sites and methylation level (average beta value) and is assigned with a unique identifier (Fowler-Noll-Vo FNV-1a non-cryptographic hash [19]) that solely depends on positions of included cytosine (and other optional) bases and their methylation states (or nucleotide symbols for optional bases), enabling not only to group epialleles by their methylation properties but also reliably and consistently track individual epialleles of high importance across different samples or even studies. The average beta values for all extracted patterns as well as patterns themselves can be explored to optimise thresholding parameters for a genomic region of interest.

Increasing scale and depth of methylation sequencing experiments impose a requirement on the speed of data processing. Therefore, all time-consuming subtasks were implemented using optimised C/C++ subroutines and, whenever possible, linked to HTSlib, unified C library for high-throughput sequencing data processing [20]. The R package epialleleR is freely available at the Bioconductor package repository (http://bioconductor.org/packages/epialleleR/).

Reporting accuracy analyses

First, we sought to validate the accuracy of methylation reporting by epialleleR in its conventional mode (no read thresholding) as compared with three other commonly used tools for which read thresholding is not available: Bismark [5], methylKit [21] and Illumina DRAGEN Bio-IT Platform. For this purpose, we simulated large sets of paired-end bisulfite sequencing reads (2x151bp, 100 million read pairs covering human chromosome 19). In contrast to real datasets, simulated data allows to calculate "ground truth" methylation levels for unbiased comparison. Simulation parameters were selected to obtain exact methylation levels of 50% for cytosines in CpG context (n=2211240) and

methylation level of approximately 0.25% (bisulfite conversion rate of ~99.75%) for cytosines in CHG and CHH contexts (n=6593900 and 19210572, respectively). In addition to endogenous deamination events [8], bisulfite treatment-induced changes [10] and variation in conversion rates [22], sequencing itself can introduce errors that vary in range depending on assay type and sequencing technology [11]. Therefore, we introduced variable level of artificial sequencing errors (0%, 0.1%, 0.3% or 0.6%) and evaluated their effect on the accuracy of reported methylation metrics, applying a selected set of methods (for comparison see Table 1). Analysis on exactly the same task (BAM file to cytosine report) revealed that reported values were close to their theoretical expectations for all methods, with epialleleR being the least affected by sequencing errors, i.e., maintaining the smallest deviance of reported versus expected methylation beta values for all samples with sequencing errors introduced, possibly owing to read quality-assisted merging of paired reads (Table 2, further details in Supplementary table 1).

Table 1. Selected characteristics of software/hardware solutions for methylation reporting.

method	requires reference (genomic) sequence	removes overlaps within read pairs	outputs epiallele frequencies	processing speed, read pairs per second
Bismark	yes	yes (trims read2)	no	40–2,800
methylKit	no	yes (trims read2)	no	9,900–15,400
DRAGEN	yes	yes (trims read2)	no	2,000–183,000
epialleleR	no	yes (base with the highest quality is chosen)	yes	129,000–231,000

Table 2. Selected accuracy metrics (average beta values and their variance) of methylation reporting. Average reported beta values that are closest to the expected beta values (0.0025 for cytosines in CHG/CHH contexts and 0.5 for cytosines in CG context) and lowest variance values are shown in bold.

sequencing error rate	method	CHH		CHG		CpG	
		mean	variance	mean	variance	mean	variance
0.00%	DRAGEN	**0.002501**	1.28E-05	**0.002500**	1.27E-05	0.499997	5.96E-07
	Bismark	0.002501	1.34E-05	0.002500	1.33E-05	**0.499997**	**5.92E-07**
	methylKit	0.002503	**1.28E-05**	0.002501	**1.27E-05**	0.499997	5.97E-07

	epialleleR	**0.002501**	1.28E-05	**0.002500**	1.27E-05	0.499997	5.96E-07
0.10%	DRAGEN	0.002661	1.37E-05	0.002662	1.36E-05	0.499835	1.73E-06
	Bismark	0.002646	1.42E-05	0.002648	1.41E-05	0.499850	1.69E-06
	methylKit	0.002662	1.37E-05	0.002664	1.36E-05	0.499835	1.75E-06
	epialleleR	**0.002624**	**1.35E-05**	**0.002626**	**1.34E-05**	**0.499872**	**1.54E-06**
0.30%	DRAGEN	0.002977	1.53E-05	0.002980	1.53E-05	0.499504	3.22E-06
	Bismark	0.002928	1.57E-05	0.002931	1.57E-05	0.499554	3.03E-06
	methylKit	0.002978	1.52E-05	0.002982	1.52E-05	0.499506	3.21E-06
	epialleleR	**0.002857**	**1.47E-05**	**0.002860**	**1.47E-05**	**0.499628**	**2.59E-06**
0.60%	DRAGEN	0.003498	1.79E-05	0.003506	1.78E-05	0.498942	1.29E-05
	Bismark	0.003393	1.81E-05	0.003402	1.81E-05	0.499051	1.25E-05
	methylKit	0.003497	1.78E-05	0.003501	1.78E-05	0.498952	1.28E-05
	epialleleR	**0.003237**	**1.66E-05**	**0.003241**	**1.65E-05**	**0.499222**	**1.14E-05**

Sensitivity analyses

Concordantly methylated alleles (alleles with most of their CpGs methylated) are thought to possess high biological importance [3,15]. Spontaneous 5-methyl cytosine (5mC) deamination, sequencing errors, as well as genuine single-nucleotide methylation/demethylation events affect observed background methylation level and can therefore hinder the detection of low-frequency hyper- or hypomethylated alleles. Differences in experimental conditions provide an additional level of variability which can sometimes be tackled by normalisation during postprocessing [23]. In contrast to the DNA methylation analysis using BeadChip arrays (such as Illumina HumanMethylation450 and HumanMethylationEPIC) which report average methylation values at the level of individual cytosines only, next-generation sequencing provides an additional data dimension by linking methylation levels of individual nucleotides within a genomic region covered by a sequencing read (epihaplotypes). However, this information is lost when methylation is assessed and reported without accounting for its allelic distribution. To evaluate the sensitivity of detection for low-frequency monoallelic hypermethylation events in next-generation sequencing data, we simulated an extended set of samples

using real, amplicon-based bisulfite sequencing data for human WBC (n=10 with almost no hypermethylated alleles, as described in Materials and Methods) and fully methylated control DNA samples. Combining real WBC DNA bisulfite sequencing data allowed to introduce sample-to-sample variability although maintaining biologically relevant background methylation levels across sequenced regions, while admixing fully methylated reads simulated low-frequency, concordant methylation events. The amplicons used, covered promoter regions of the tumour suppressors *MLH1*, *CDKN2A*, *MGMT*, *CDH1*, and *BRCA1*. The distributions of per-read beta values (Supplementary Fig. 1) and methylation patterns (Supplementary Fig. 2) of admixed samples show the expected abundance of hypermethylated (average $\beta \geq 0.5$) alleles and confirm their high similarity to the real samples (Supplementary Figs 3 and 4). Conventional cytosine reports (no read thresholding) as well as VEF reports (with read thresholding) were prepared and used for unsupervised clustering of samples and differentially methylated region (DMR) discovery. Despite quite low overall methylation level of amplified regions (average beta value of 0.014, median of 0.005; Fig. 2A), t-SNE analysis based on beta values was not able to discriminate between samples with 0.01%, 0.03%, 0.10%, 0.30% of methylated reads, or no methylated reads added (Fig. 2B, left panel). On the other hand, VEF value-based t-SNE analysis resulted in spatially well-separated clusters that corresponded to each level of admixed methylated reads (Fig. 2B, right panel). Intergroup DMR discovery based on beta values (Fig. 2C, left panel) showed fewer number of regions found as well as higher associated false discovery rate (FDR), while discovery based on VEF values resulted in all five possible regions identified for all possible intergroup comparisons as well as generally lower associated FDR. When each sample with admixed methylated reads was compared against the group of samples without admixed methylated reads, recall metrics for differential (by DMRcate [24]; Fig. 2D) or aberrant (by ramr [12]; Fig. 2E) methylation analysis were notably higher for analyses based on VEF values (Fig. 2D–E, right panels) in comparison with analyses based on beta values (Fig. 2D–E, left panels). This shows that VEF values are more valuable for detection and analysis of low-frequency (≤1%) hypermethylation events than methylation beta values.

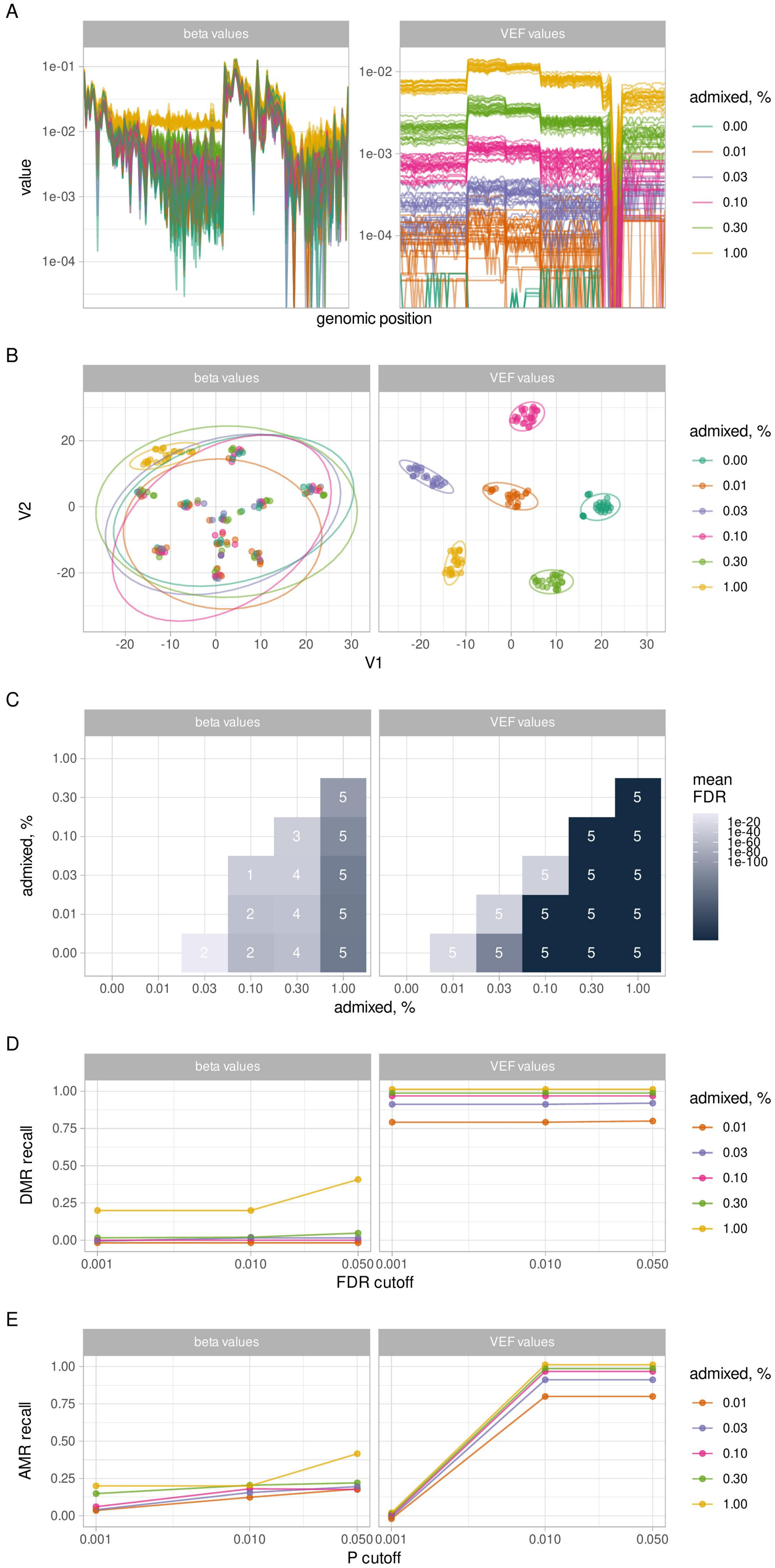
A
beta values
VEF values
1e-01
1e-02
1e-03
1e-04
value
genomic position
admixed, %
0.00
0.01
0.03
0.10
0.30
1.00
B
V1
V2
20
0
-20
-10
10
30
C
admixed, %
mean FDR
1e-20
1e-40
1e-60
1e-80
1e-100
D
DMR recall
FDR cutoff
0.001
0.010
0.050
0.25
0.50
0.75
E
AMR recall
P cutoff

Figure 2. (A) Line plots of beta (left panel) and VEF (right panel) values for individual samples, colour-coded according to the amount of admixed methylated reads. Each line represents a sample; y-axis, methylation value of all CpGs (n=138) sorted by their genomic position (categorical x-axis). (B) Embedding plots for t-SNE analysis using beta (left panel) and VEF (right panel) values. Ellipses represent 95% confidence levels. (C) Heatmap of mean false discovery rate for differentially methylated regions (DMRs) identified by DMRcate. Labels indicate the number of DMRs found (of total of five regions possible). (D) Recall rate for DMR identification using DMRcate for varying FDR cutoffs. (E) Recall rate for aberrantly methylated regions identification using ramr for varying p value cutoffs.

BeadChip arrays, such as Illumina HumanMethylationEPIC, is another widely used, amplification-free method to assess genome-wide DNA methylation for a reduced cost. In order to directly compare the sensitivities of targeted NGS and of the BeadChip arrays for the detection of low-frequency DNA methylation events, we employed both of the methods to analyse small set of samples (n=8) carrying low-frequency methylation in at least one of the assayed regions (promoter regions of *MLH1*, *CDKN2A*, *MGMT*, *CDH1*, and *BRCA1*). Sample distributions of per-read beta values (Supplementary Fig. 3) and methylation patterns (Supplementary Fig. 4) show that these samples indeed contain varying frequencies of hypermethylated (average $\beta \geq 0.5$) alleles. For unbiased comparison, we limited the corresponding data sets to the CpGs assayed and sufficiently covered by both techniques. Analysis revealed that VEF values of samples with many hypermethylated alleles (e.g., A26 and A45 for *BRCA1*; as apparent from Fig. 3A) differ significantly (Fig. 3B) from VEF values of samples with only a few or no hypermethylated alleles (e.g., A02 or A05 for *BRCA1*; Fig. 3A). When VEF values were used for identification of aberrantly or differentially methylated regions by ramr [12] or DMRcate [24], respectively, the significant regions found correlated well with the notable presence of hypermethylated alleles. Of note, slightly inferior performance of DMRcate is probably due to the fact that for some of the genomic regions too many samples in this subset simultaneously contained hypermethylated epialleles. When DMRcate was used for the same purpose on an extended set of sequenced samples (n=18, containing n=10 samples characterised by the absence of hypermethylated alleles that were used to create admixed sample set), its performance in identification of hypermethylated epiallele-containing samples was higher (Supplementary Fig. 5).

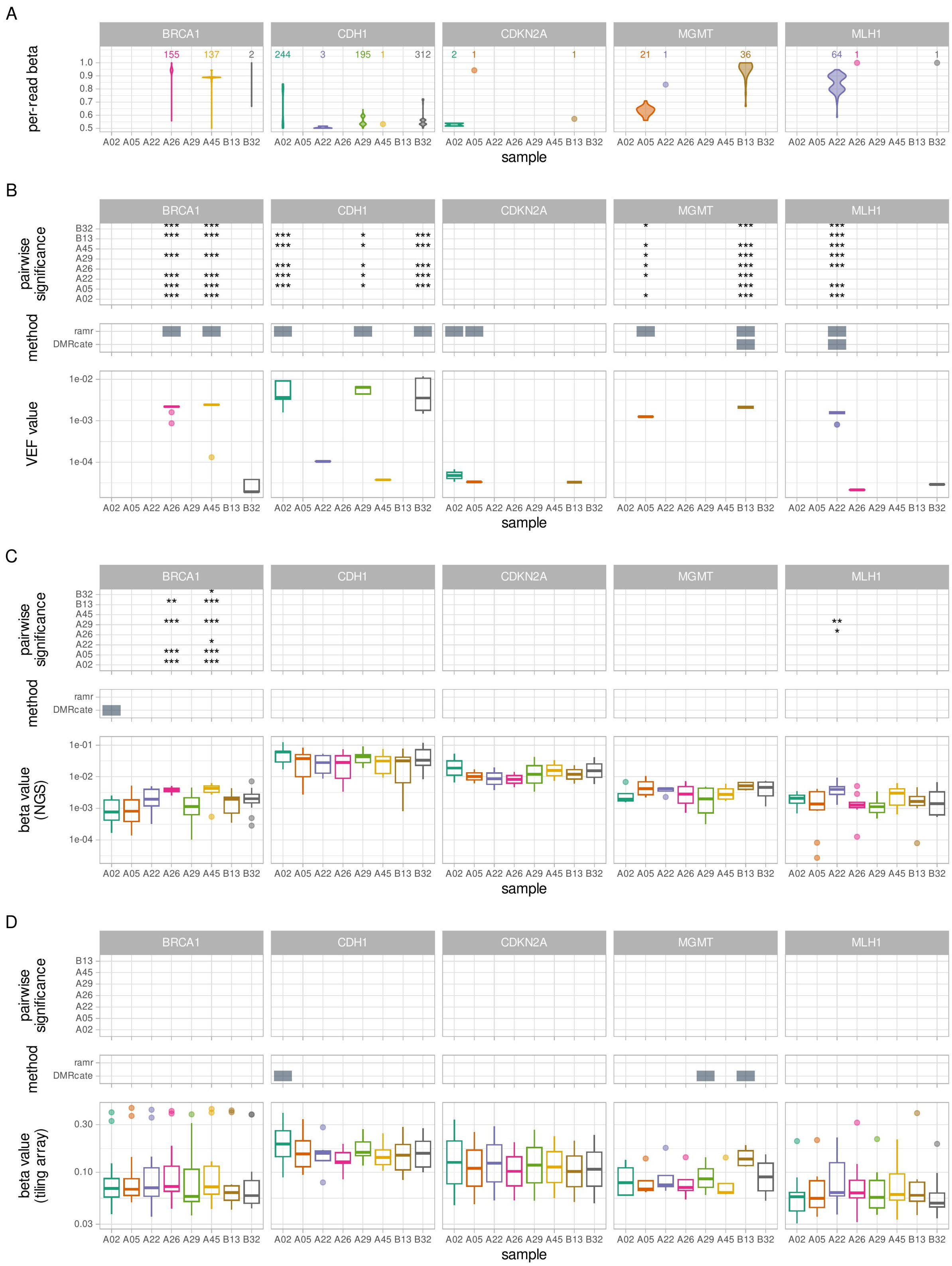

Figure 3. (A) Distribution of per-read beta values for NGS read pairs covering CpGs that are common for NGS and BeadChip array. For clarity, only the reads with average beta of at least 0.5 (i.e., representing hypermethylated epialleles) are included. Single observations are shown as dots, number of observations is given above. Complete density plots are provided

in the Supplementary Fig. 3. Corresponding methylation patterns are provided in the Supplementary Fig. 4. (B) Lower panel: box plots of NGS-derived VEF values for individual CpGs; middle panel: significant aberrantly or differentially methylated regions identified by ramr or DMRcate, respectively, based on VEF values; upper panel: significance levels from pairwise comparison of VEF values. (C) Lower panel: box plots of NGS-derived beta values for individual CpGs; middle panel: significant aberrantly or differentially methylated regions identified by ramr or DMRcate, respectively, based on NGS-derived beta values; upper panel: significance levels from pairwise comparison of NGS-derived beta values. (D) Lower panel: box plots of BeadChip array-derived beta values for individual CpGs; middle panel: significant aberrantly or differentially methylated regions identified by ramr or DMRcate, respectively, based on BeadChip array-derived beta values; upper panel: significance levels from pairwise comparison of BeadChip array-derived beta values. (B–D) The lower and upper hinges of boxes correspond to the first (Q_1) and third (Q_3) quartiles; the bar in the middle correspond to the median value; the upper and lower whisker extend to Q_3+1.5*IQR and Q_1-1.5*IQR, respectively, while the values outside this range (outliers) are plotted as dots. Zero values are not plotted. *** $p<0.001$, ** $p<0.01$, * $p<0.05$, blank $p\geq0.05$.

In contrast, only a few significant differences remained when NGS beta values were used for sample comparison (Fig. 3C), while pairwise comparisons based on BeadChip array beta values did not reveal any significant differences between samples (Fig. 3D). The search for aberrantly or differentially methylated regions using either NGS or array beta values did not result in identification of such regions in relevant (according to methylation patterns or beta value densities) samples. Generally higher beta values of BeadChip array as compared to NGS beta values likely mask subtle changes in methylation caused by the presence of infrequent hypermethylated alleles and hinder the detection of differences between samples.

Several scores to describe and quantify variability in DNA methylation in sequencing reads (within-sample heterogeneity, WSH) have been proposed [25]. In order to assess WSH, we evaluated the difference in combinatorial entropy between each pair of samples using methclone [26] (Supplementary Fig. 6A). The largest (by absolute value) reported difference in combinatorial entropy of -2.59 between any pair of samples confirms a high similarity between sample methylation profiles, of note, being much smaller than cutoffs for epiallele shifts between samples analysed in [25] (-60 and lower). Further, we also calculated four additional heterogeneity scores: combinatorial entropy, epipolymorphism, fraction of discordant read pairs (FDRP) and proportion of discordant reads (PDR). The scores themselves (Supplementary Fig. 6B) and the levels of score-based pairwise significance

between samples (Supplementary Fig. 6C), are not generally consistent with fractions of hypermethylated (average $\beta \geq 0.5$) alleles (Fig. 3A and Supplementary Fig. 3) or VEF values (Fig. 3B): e.g., samples A26 and A45 have a notable fraction of hypermethylated reads in *BRCA1* promoter region compared to other samples, although it is not reflected at the level of WSH scores. Importantly, WSH scores produced cannot be directly used as an input for DMR analysis tools, which are commonly employed to characterise exact differences in methylation between samples.

It is known that DNA methylation profiles of blood samples depend on the varying contribution of individual blood cell types [27,28]. While we cannot exclude that hypermethylated alleles present in the samples analysed here originate from a particular blood cell type, low-level, mosaic epimutations of at least *BRCA1* were previously shown to be independent of blood subfraction composition [1]. Of note, only one CpG (cg05785947 in *CDH1*) out of 37 used in NGS vs BeadChip array comparison here, was found to be significantly differentially methylated between blood cell types of healthy males; and none of CpGs were significantly differentially methylated between blood cell types of newborns.

Processing speed analyses

Methylation sequencing data produced by contemporary techniques varies in scale and depth and may contain several thousands to billions of single or paired-end reads. To analyse them efficiently, computational methods must be scalable and fast enough for as large as possible range of sample counts or data file sizes. Unfortunately, many academic tools use computationally complex algorithms that do not scale to contemporary tasks. We compared data processing speed for epialleleR versus methylKit, Bismark, and DRAGEN Bio-IT Platform, performing exactly the same task (BAM file to cytosine report) of methylation reporting across input data coming from various assays: amplicon-based (n=10 samples with a depth of coverage of ~20,000x), genome-wide capture-based (n=10 with a depth of coverage of ~60x and n=3 with a depth of coverage of ~1000x) or whole-genome bisulfite sequencing (WGBS, n=6 with a depth of coverage of ~60x). The obtained results confirm very

efficient implementation of epialleleR and its suitability for analysis of data sets of any depth and coverage (Fig. 4, Table 1).

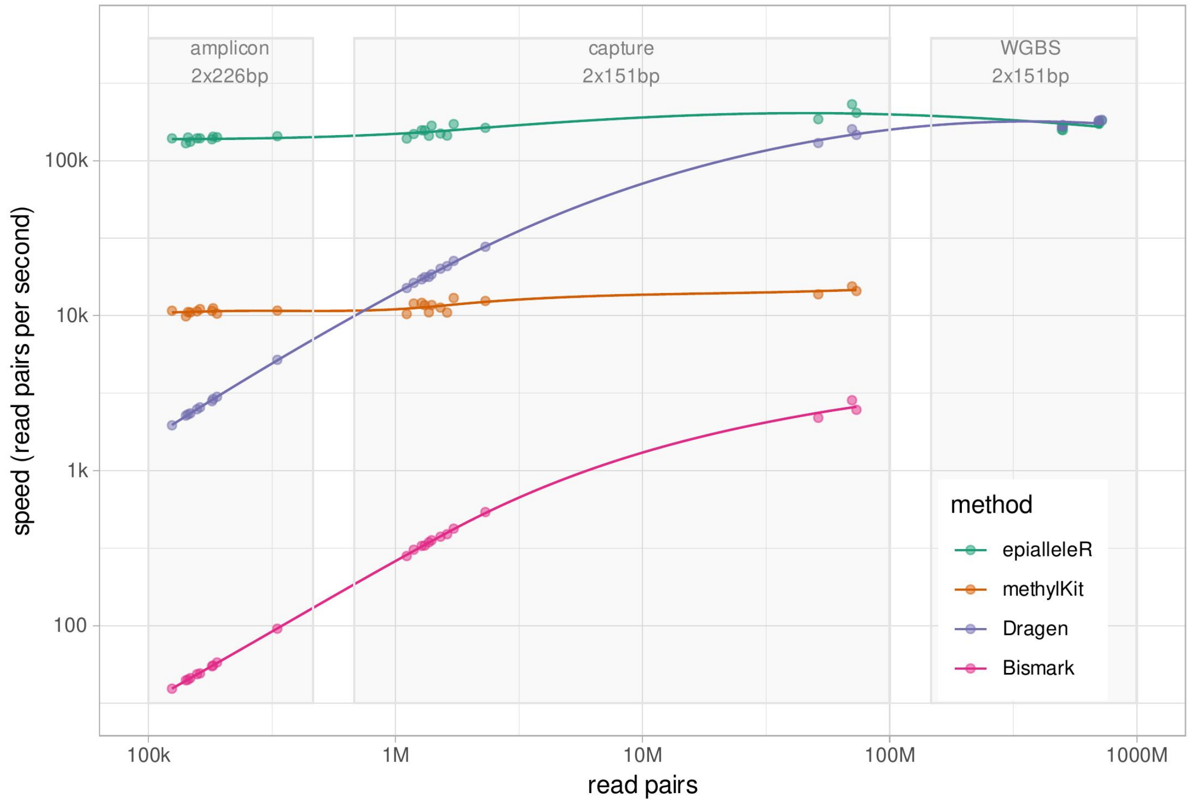

Figure 4. Data processing speed (in read pairs per second) of epialleleR as compared to three other methods for methylation reporting (methylKit, Bismark, and DRAGEN Bio-IT Platform). Read count (in number of pairs) is given at x-axis; light grey boxes outline data obtained by targeted amplicon-based, genome-wide capture-based, or whole-genome bisulfite sequencing.

DISCUSSION

While conflicting data has linked low-level mosaic primary constitutional epimutations to cancer risk for more than a decade [29], we have recently obtained firm evidence implicating primary epimutations within the *BRCA1* gene in an elevated risk of incident breast and ovarian cancer [3]. The assumption that such epimutations may affect other tumour suppressor genes and, therefore, lead to

other cancer forms [29], institutes a new research area with respect to cancer risk. Further, the findings of such epimutations in umbilical cord blood [1] indicate prenatal events of a yet unknown genesis. This creates the need for multidisciplinary studies on the mechanisms of these events and on their effects in respect to cancer risk, as well as the need for ultrasensitive methods allowing sample assessment at a high scale.

Here, we present the details on a fast, accurate, and sensitive method to detect, quantify and visualise epialleles in NGS data. The method shows its superiority versus conventional methods of methylation reporting, especially when applied for detection of low-frequency methylation events, as it is by design less susceptible to variations in conversion efficiency or sequencing quality. Although epialleleR is not a differential methylation analysis tool, its output can be directly used to group samples based on their methylation profiles (by applying a simple threshold or using unsupervised clustering), as well as an input for other differential/aberrant methylation analysis software (the latter is not possible for WSH analysis tools).

We thoroughly tested epialleleR using bisulfite sequencing data; the method, however, can also be applied to analyse and compare data obtained using any methylation sequencing technique (reduced representation bisulfite sequencing, RRBS; oxidative bisulfite sequencing, oxBS-Seq; Tet-assistant bisulfite sequencing, TAB-Seq), as long as methylation in these data can be called at individual cytosine residues instead of being analysed by comparing relative abundance of the fragments (such as for methylation sensitive restriction enzyme sequencing, MRE-Seq, or methylated DNA immunoprecipitation sequencing, MeDIP-Seq).

The possibility to call cytosine methylation for alignment files created by different short sequence aligners, subtle though noticeable changes in cytosine reporting accuracy together with immense speed gain make epialleleR a method of choice not only for discovery of infrequent hypermethylated epialleles (as in [3]), but also as a tool to produce conventional (no read thresholding) cytosine reports from any methylation sequencing alignment files.

The implemented method is fully documented and can be easily used from within the R environment for statistical computing. With the epialleleR already revealing its suitability for detection of low-level mosaic methylation events in a large data set [3], we believe it constitutes an optimal tool for assessment of low-level mosaic epimutations with respect to risk of cancer as well as other diseases of relevance.

CONCLUSIONS

Here, we present epialleleR, very fast, accurate, and sensitive method to detect, quantify and visualise epialleles in NGS data. Efficient implementation and improvements in cytosine reporting accuracy allow us to recommend epialleleR not only for analysis of methylation patterns and to enhance low-level differentially methylated region discovery, but also as a conventional cytosine reporting tool for various kinds of methylation sequencing data. The epialleleR R/Bioconductor package is freely available at https://bioconductor.org/packages/epialleleR/ and https://github.com/BBCG/epialleleR.

MATERIALS AND METHODS

Next-generation sequencing

White blood cells (WBC) DNA samples from anonymized males (n=88) [30,31] and human HCT116 DKO methylated DNA control sample (Zymo Research, cat.no. D5014-2) were bisulfite converted, and five DNA fragments, representing promoter regions of five established tumour suppressor genes, were amplified using custom set of primers (GRCh38 assembly coordinates of assayed regions: *MLH1*, chr3:36993123–36993500; *CDKN2A*, chr9:21974554–21974921; *MGMT*, chr10:129467118–129467477; *CDH1*, chr16:68737102–68737469; *BRCA1*, chr17:43125171–43125550), indexed, and sequenced similarly to as previously described [3] (GSE201688). The resulting average coverage was 5000x–50000x per amplicon.

Bioinformatic and statistical analyses

Massive parallel sequencing (NGS) reads were mapped/aligned to the GRCh38 human reference genome and the methylation was called using Illumina DRAGEN Bio-IT Platform (v3.9.5) with the following parameters: --methylation-mapping-implementation single-pass, --enable-methylation-calling true, --methylation-generate-cytosine-report false, --methylation-protocol non-directional, --enable-sort false, unless stated otherwise. R software environment for statistical computing (v4.1.2) was used for all downstream statistical analyses.

The frequency of hypermethylated alleles across assayed regions in n=88 male WBC DNA samples were estimated using epialleleR::generateAmpliconReport with the following parameters: min.mapq=30, min.baseq=20, nthreads=4, threshold.reads=TRUE, report.context="CG", and bed.file pointing to a location of BED (browser extensible data) file with genomic regions amplified (see amplicon coordinates above). Two sample subgroups (n=8 and n=10) were selected for sensitivity analyses based on the frequencies of hypermethylated alleles as explained below.

Cytosine reporting accuracy comparison

Four sets of paired-end sequencing reads (151bp, 50 million read pairs each set) were simulated using Sherman Bisulfite FastQ Read Simulator (RRID:SCR_001294) [32] with the following options: --length 151, --number_of_seqs 50000000, --paired_end, --minfrag 70, --maxfrag 400, --CG_conversion 0, --CH_conversion 99.5 and varying sequencing error rate (--error_rate parameter) of 0%, 0.1%, 0.3% or 0.6%. Human chromosome 19 sequence (GRCh38.p13 NC_000019.10, 58617616 bp, 1105620 forward strand CpGs) was used as a reference genome for read simulation and mapping/alignment due to its highest CpG content across all human chromosomes [33] and in order to maintain optimal balance of analysis speed and base coverage. Each set of reads was then duplicated, all read1 cytosines (C) and read2 guanines (G) in any context in the duplicate sets were replaced with thymines (T) and adenines (A), respectively. Then, duplicate sets (i.e., unmethylated reads) were merged with original sets (i.e., methylated reads) resulting in four sets of reads 100 million pairs each, with the cytosine conversion rate of exactly 50% and about 99.75% in CpG and non-CpG contexts, respectively.

The mapping and alignment of simulated reads was performed using Illumina DRAGEN Bio-IT Platform v3.9.5 with the following modification in parameters: --methylation-protocol directional. Methylation reporting by all tools was done as described below (reporting parameters in Speed comparison section).

Sensitivity comparison on admixed samples

In order to simulate variable methylation levels while maintaining biological heterogeneity of the samples, we selected ten male DNA NGS samples with the lowest frequency of hypermethylated alleles across all assayed regions, then admixed varying fractions of reads from two random samples and additionally "spiked" certain number of fully methylated reads from methylated DNA control sample. This resulted in 150 samples containing 0%, 0.01%, 0.03%, 0.1%, 0.3% or 1% of methylated reads per sample (25 samples per every category).

Read mapping, alignment, methylation calling, and generation of genome-wide cytosine reports was performed using Illumina DRAGEN Bio-IT Platform as described above. VEF calling was performed using epialleleR::generateCytosineReport with the following parameters: min.mapq=0, min.baseq=0, nthreads=4, threshold.reads=TRUE, report.context="CG".

Methylation patterns and per-read beta values for all samples were extracted using epialleleR::extractPatterns with the following parameters: min.mapq=30, min.baseq=20, nthreads=4, clip.patterns=FALSE, and bed.file pointing to a location of BED file with genomic regions amplified (see amplicon coordinates above).

Barnes-Hut t-Distributed Stochastic Neighbor Embedding (t-SNE) analysis was performed using R package Rtsne v0.15 [34] and matrices of beta or VEF values for all genomic positions of CpGs with the coverage of at least 1000x and available values for all analysed samples (total number of CpGs, n=138; *MLH1*, n=20; *CDKN2A*, n=35; *MGMT*, n=33; *CDH1*, n=32; *BRCA1*, n=18).

Sensitivity comparison to methylation array data

Eight additional WBC DNA NGS samples from anonymized males carrying hypermethylated alleles in at least one of the assayed regions were selected, and VEF calling was performed using epialleleR::generateCytosineReport with the following parameters: min.mapq=30, min.baseq=20, nthreads=4, threshold.reads=TRUE, report.context="CG". The same DNA samples were also bisulfite converted using the Zymo EZ DNA Methylation Kit (Zymo Research, cat.no. D5001), and genome-wide methylation levels were assessed using Illumina HumanMethylationEPIC BeadChip arrays according to manufacturer's instructions. Resulting IDAT files were processed (normalized and annotated) with the minfi Bioconductor package [23] using the preprocessQuantile method with outlier thresholding enabled (GSE201689). For direct comparison, only the CpGs that are covered in all samples by both BeadChip arrays (p-value of 0) and targeted sequencing (minimum sequencing coverage of 5000x) were retained (*MLH1*, n=10; *CDKN2A*, n=2; *MGMT*, n=4; *CDH1*, n=7; *BRCA1*, n=14). Pairwise sample comparison was performed using t-test with Holm adjustment for multiple comparisons.

The sets of CpGs that are differentially methylated between cell blood types were reported previously: DNA methylation profiles for six blood cell types from six males [27,35], and DNA methylation profiles for seven blood cell types from cord blood of 104 newborns [28,36]. CpG-level differential methylation analysis p values were Holm-adjusted and the ones remained significant (adjusted $p \leq 0.05$; n=73629 of total 456655 for male blood data set; n=221246 of total 429794 for newborn cord blood data set) were checked for overlap with the set of CpGs analysed in this study (n=35 CpGs of total n=37 were present in each of male/newborn data sets).

Differential methylation analysis

Differentially methylated regions (DMRs) were called using R package DMRcate (v2.12.0) with the following parameters: lambda=1000, min.cpgs=2, pcutoff="fdr" [24]. Aberrantly methylated regions were called using R package ramr (v1.6.0) with the following parameters: ramr.method="beta", min.cpgs=2, merge.window=500 [12]. To enable maximum likelihood estimation of beta distribution parameters, all zeros were replaced with minimum double values (2.26e-308).

For intergroup DMR discovery in admixed samples, pairwise comparison of sample groups defined by the amount of admixed reads was performed (n=25 samples in each group) using the default level of false discovery rate (FDR) cutoff (equals 0.05). For DMR discovery in real samples, as DMRcate methods require two classes/categories for comparison, every real sample from the test dataset was tested against all the other samples using the default FDR cutoff value.

To assess DMR (or AMR) recall metrics in admixed samples, every sample with admixed reads was compared using DMRcate (or ramr) to the group of 25 samples without admixed reads at a varying level of FDR (or p value) cutoff of 0.05, 0.01, or 0.001. As the admixed reads covered all five assayed regions, only the total number of real positive regions (P, equals 5 for each comparison), the number of true positive regions (TP), and the number of false negative regions (FN=P–TP) were known, while the numbers of true negative (TN) or false positive (FP) regions were undefined. Therefore, recall, or true positive rate (TPR=TP/P) was chosen as a sensitivity metric.

Within-sample heterogeneity

Estimation of within-sample heterogeneity (WSH) was performed on eight samples used in the sensitivity comparison between array- and NGS-based methylation profiling. Difference in entropy was evaluated using methclone (v1) [26] with a distance cutoff of 500 and minimum read coverage of 1000 for every pair of samples. As methclone outputs values for multiple genomic regions, the minimum value (representing absolute largest difference) was selected and used further. Entropy, epipolymorphism, fraction of discordant read pairs (FDRP) and proportion of discordant reads (PDR) were evaluated using R package WSH (v0.1.6) [25] with the following options: mapq.filter=30, window.size=500, and bam.file pointing to a location of BAM file. Due to exponential complexity of FDRP calculation, option max.reads was set to 100 for FDRP calculation and to 1e+06 otherwise. Pairwise sample score comparison was performed using t-test with Holm adjustment for multiple comparisons.

Processing speed comparison

Comparison of processing speed was performed on 29 BAM files containing paired-end alignments and methylation calls derived from bisulfite sequencing of human WBC DNA samples prepared using the following assays: A) amplicon-based sequencing of promoter regions of *BRCA1* gene (n=10 files, 0.12–0.33 million read pairs per file, average coverage of ~20,000x) [3]; B) genome-wide capture-based bisulfite sequencing of promoter regions of 283 tumour suppressor genes (n=10 files, 1.11–2.31 million read pairs per file, average coverage of ~60x; and n=3 files, 51.4–73.4 million read pairs per file, average coverage of ~1000x) [37,38]; C) whole-genome bisulfite sequencing (n=6 files, 497–723 million read pairs per file, average coverage of ~60x; epialleleR and Illumina DRAGEN Bio-IT Platform only). The two former data sets (A and B) were generated in-house and described previously, while the latter data (C) were obtained from NCBI Sequence Read Archive (GEO/SRA samples GSM3683953/SRX6640720, GSM3683958/SRX6640725, GSM3683965/SRX6640732, GSM3683951/SRX6640718, GSM3683955/SRX6640722, and GSM3683962/SRX6640729) and reported elsewhere [39].

Processing times to produce conventional cytosine reports were recorded as following:
Bismark CX methylation reports were created using Bismark v0.22.3 (RRID:SCR_005604) [5] with the following parameters: command bismark_methylation_extractor, --paired-end, --no_overlap, --comprehensive, --gzip, --mbias_off --parallel 8, --cytosine_report, --CX, --buffer_size 64G. As parallel processing was requested, Bismark used up to 24 cores for some of its subtasks.

methylKit CX methylation reports were created using R/Bioconductor package methylKit v1.20.0 (RRID:SCR_005177) [21] with the following parameters: function methylKit::processBismarkAln, minqual=0, mincov=0, save.context=c("CpG","CHG","CHH"), nolap=TRUE and location pointing to the location of a BAM file. Parallel processing is currently not available for methylKit::processBismarkAln.

epialleleR CX methylation reports were created using R/Bioconductor package epialleleR v1.3.5 with the following parameters: function epialleleR::generateCytosineReport, min.mapq=0, min.baseq=0, nthreads=4 (number of HTSlib decompression threads), threshold.reads=FALSE,

report.context="CX" and bam pointing to the location of a BAM file. epialleleR methods currently run in a single-threaded mode only but can benefit from additional BAM decompression threads provided by HTSlib.

Illumina DRAGEN is a hardware solution that relies on the presence of FPGA accelerator card, which precludes DRAGEN software execution on other platforms. At the same time, outdated software development tools available at DRAGEN (GCC v4.8.5, R v3.6.0) impede installation of third-party software and R/Bioconductor packages and may potentially affect their performance. Therefore, testing of methylation reporting tools was carried out in two different settings.

Bismark, methylKit and epialleleR were tested on the workstation equipped with AMD EPYC 7742 64-core processor, 512GB of memory and the Red Hat Enterprise Linux Server release 7.9 (Developer Toolset 6, GCC v6.3.1), with BAM files retrieved from high-speed (10Gbps) network accessible storage.

DRAGEN CX methylation reports were created using Illumina DRAGEN Bio-IT Platform v3.9.5 (Intel Xeon Gold 6126 48-core processor, 256GB of memory and CentOS Linux release 7.5.1804) with the following parameters: --methylation-generate-cytosine-reports=true, --enable-sort=false, --enable-duplicate-marking=false, --methylation-report-only=true and --bam-input pointing to the location of a BAM file. Default number of threads (up to 24) were used for data processing using DRAGEN; BAM files were accessed from local, high-speed NVMe solid state disk.

For Bismark and DRAGEN, elapsed time measurements were stably reproducible, thus processing time was recorded only once for each file. For methylKit and epialleleR, the tests were run five times in sequential random order by means of R package microbenchmark v1.4.9, and the average time was used in comparison to mitigate variability in processing time measurements.

DECLARATIONS

Ethics approval and consent to participate

Ethics approvals and other relevant information for patient-generated data used in speed assessment were included and described in previous studies [3,30,37,38]. All analyses of biomaterial were approved by Regional Ethics Committees for medical research and all samples were collected after written informed consent from the sample donors (REK-vest Norway reference numbers: 3.2008.1932, 2015/1493 and 2018/1566.

Availability of data and materials

The epialleleR R/Bioconductor package (biotools:epialleleR, RRID:SCR_023913) is freely available at https://bioconductor.org/packages/epialleleR/ and https://github.com/BBCG/epialleleR. The R scripts used in this manuscript and the data underlying accuracy and sensitivity analyses are freely available at DataverseNO (https://doi.org/10.18710/2BQTJP). Sensitive data used for the processing speed assessment are available from the authors in accordance with study protocols.

Public data for sensitivity analysis have been deposited at NCBI Gene Expression Omnibus under accession number GSE201690. Public whole-genome bisulfite sequencing data used for the processing speed assessment are available at NCBI Sequencing Read Archive under accession number SRP217135.

Supplementary Data are available online.

Previous use of epialleleR

A previous version of this manuscript was deposited in bioRxiv (doi: 10.1101/2022.06.30.498213) and the epialleleR method has been applied in [3].

Competing interests

P.E.L. has for other projects received research funding from AstraZeneca, Novartis, Pfizer, and Illumina, and honoraria through speaker's bureaux from AstraZeneca, Pierre-Fabre, Roche, AbbVie and Akademikonferens. He has participated in advisory boards for AstraZeneca, Laboratorios and Farmaceuticos Rovi. S.K. has received research funding for other projects from AstraZeneca, Pfizer, and Illumina, and speaker's bureaux honoraria from AstraZeneca, Pfizer, Novartis, and Pierre Fabre.

Funding

This work was supported by the K.G.Jebsen foundation [grant number SKGJ-MED-020 to P.E.L.]; The Norwegian Cancer Society [grant number 190281-2017 to S.K.]; and The Norwegian Research Council [grant number 617344-1 to P.E.L.]. Funding for open access charge: The Norwegian Research Council.

Authors contribution

Conceived the project: O.N., P.E.L., S.K. Supervised the project: P.E.L., S.K. Conceived, designed, and implemented the software and the analysis pipeline: O.N. Wrote the paper: O.N., P.E.L., S.K. All authors read and approved the final manuscript.

REFERENCES

1. Lønning PE, Berge EO, Bjørnslett M, Minsaas L, Chrisanthar R, Høberg-Vetti H, et al.. White Blood Cell BRCA1 Promoter Methylation Status and Ovarian Cancer Risk. *Ann Intern Med*. American College of Physicians; 2018; doi: 10.7326/M17-0101.

2. Prajzendanc K, Domagała P, Hybiak J, Ryś J, Huzarski T, Szwiec M, et al.. BRCA1 promoter methylation in peripheral blood is associated with the risk of triple-negative breast cancer. *International Journal of Cancer*. 2020; doi: https://doi.org/10.1002/ijc.32655.

3. Lønning PE, Nikolaienko O, Pan K, Kurian AW, Eikesdal HP, Pettinger M, et al.. Constitutional BRCA1 Methylation and Risk of Incident Triple-Negative Breast Cancer and High-grade Serous Ovarian Cancer. *JAMA Oncol*. 2022; doi: 10.1001/jamaoncol.2022.3846.

4. Sun R, Zhu P. Advances in measuring DNA methylation. *Blood Sci*. 2022; doi: 10.1097/BS9.0000000000000098.

5. Krueger F, Andrews SR. Bismark: a flexible aligner and methylation caller for Bisulfite-Seq applications. *Bioinformatics*. 2011; doi: 10.1093/bioinformatics/btr167.

6. Maksimovic J, Phipson B, Oshlack A. A cross-package Bioconductor workflow for analysing methylation array data. *F1000Res*. 2016; doi: 10.12688/f1000research.8839.3.

7. Fortin J-P, Triche TJ Jr, Hansen KD. Preprocessing, normalization and integration of the Illumina HumanMethylationEPIC array with minfi. *Bioinformatics*. 2017; doi: 10.1093/bioinformatics/btw691.

8. Youk J, An Y, Park S, Lee J-K, Ju YS. The genome-wide landscape of C:G > T:A polymorphism at the CpG contexts in the human population. *BMC Genomics*. 2020; doi: 10.1186/s12864-020-6674-1.

9. Gu J, Stevens M, Xing X, Li D, Zhang B, Payton JE, et al.. Mapping of Variable DNA Methylation Across Multiple Cell Types Defines a Dynamic Regulatory Landscape of the Human Genome. *G3 (Bethesda)*. 2016; doi: 10.1534/g3.115.025437.

10. Kint S, Spiegelaere WD, Kesel JD, Vandekerckhove L, Criekinge WV. Evaluation of bisulfite kits for DNA methylation profiling in terms of DNA fragmentation and DNA recovery using digital PCR. *PLOS ONE*. Public Library of Science; 2018; doi: 10.1371/journal.pone.0199091.

11. Stoler N, Nekrutenko A. Sequencing error profiles of Illumina sequencing instruments. *NAR Genomics and Bioinformatics*. 2021; doi: 10.1093/nargab/lqab019.

12. Nikolaienko O, Lønning PE, Knappskog S. ramr: an R/Bioconductor package for detection of rare aberrantly methylated regions. *Bioinformatics*. 2021; doi: 10.1093/bioinformatics/btab586.

13. Kondrashova O, Topp M, Nesic K, Lieschke E, Ho G-Y, Harrell MI, et al.. Methylation of all BRCA1 copies predicts response to the PARP inhibitor rucaparib in ovarian carcinoma. *Nat Commun*. 2018; doi: 10.1038/s41467-018-05564-z.

14. Nesic K, Kondrashova O, Hurley RM, McGehee CD, Vandenberg CJ, Ho G-Y, et al.. Acquired RAD51C Promoter Methylation Loss Causes PARP Inhibitor Resistance in High-Grade Serous Ovarian Carcinoma. *Cancer Res*. 2021; doi: 10.1158/0008-5472.CAN-21-0774.

15. Hurley RM, McGehee CD, Nesic K, Correia C, Weiskittel TM, Kelly RL, et al.. Characterization of a RAD51C-silenced high-grade serous ovarian cancer model during development of PARP inhibitor resistance. *NAR Cancer*. 2021; doi: 10.1093/narcan/zcab028.

16. Huh I, Wu X, Park T, Yi SV. Detecting differential DNA methylation from sequencing of bisulfite converted DNA of diverse species. *Briefings in Bioinformatics*. 2019; doi: 10.1093/bib/bbx077.

17. Anastasiadi D, Esteve-Codina A, Piferrer F. Consistent inverse correlation between DNA methylation of the first intron and gene expression across tissues and species. *Epigenetics & Chromatin*. 2018; doi: 10.1186/s13072-018-0205-1.

18. R Core Team. R: A Language and Environment for Statistical Computing. Vienna, Austria: R Foundation for Statistical Computing;

19. Fowler G, Noll LC, Vo K-P, Eastlake 3rd DE, Hansen T. The FNV Non-Cryptographic Hash Algorithm. Internet Engineering Task Force; Report No.: draft-eastlake-fnv-17.

20. Bonfield JK, Marshall J, Danecek P, Li H, Ohan V, Whitwham A, et al.. HTSlib: C library for reading/writing high-throughput sequencing data. *GigaScience*. 2021; doi: 10.1093/gigascience/giab007.

21. Akalin A, Kormaksson M, Li S, Garrett-Bakelman FE, Figueroa ME, Melnick A, et al.. methylKit: a comprehensive R package for the analysis of genome-wide DNA methylation profiles. *Genome Biol*. 2012; doi: 10.1186/gb-2012-13-10-r87.

22. Sun Z, Vaisvila R, Hussong L-M, Yan B, Baum C, Saleh L, et al.. Nondestructive enzymatic deamination enables single-molecule long-read amplicon sequencing for the determination of 5-

methylcytosine and 5-hydroxymethylcytosine at single-base resolution. *Genome Res*. 2021; doi: 10.1101/gr.265306.120.

23. Aryee MJ, Jaffe AE, Corrada-Bravo H, Ladd-Acosta C, Feinberg AP, Hansen KD, et al.. Minfi: a flexible and comprehensive Bioconductor package for the analysis of Infinium DNA methylation microarrays. *Bioinformatics*. 2014; doi: 10.1093/bioinformatics/btu049.

24. Peters TJ, Buckley MJ, Chen Y, Smyth GK, Goodnow CC, Clark SJ. Calling differentially methylated regions from whole genome bisulphite sequencing with DMRcate. *Nucleic Acids Res*. 2021; doi: 10.1093/nar/gkab637.

25. Scherer M, Nebel A, Franke A, Walter J, Lengauer T, Bock C, et al.. Quantitative comparison of within-sample heterogeneity scores for DNA methylation data. *Nucleic Acids Res*. 2020; doi: 10.1093/nar/gkaa120.

26. Li S, Garrett-Bakelman F, Perl AE, Luger SM, Zhang C, To BL, et al.. Dynamic evolution of clonal epialleles revealed by methclone. *Genome Biol*. 2014; doi: 10.1186/s13059-014-0472-5.

27. Reinius LE, Acevedo N, Joerink M, Pershagen G, Dahlén S-E, Greco D, et al.. Differential DNA methylation in purified human blood cells: implications for cell lineage and studies on disease susceptibility. *PLoS One*. 2012; doi: 10.1371/journal.pone.0041361.

28. Bakulski KM, Feinberg JI, Andrews SV, Yang J, Brown S, L McKenney S, et al.. DNA methylation of cord blood cell types: Applications for mixed cell birth studies. *Epigenetics*. 2016; doi: 10.1080/15592294.2016.1161875.

29. Lønning PE, Eikesdal HP, Løes IM, Knappskog S. Constitutional Mosaic Epimutations – a hidden cause of cancer? *Cell Stress*. Shared Science Publishers; 2019; doi: 10.15698/cst2019.04.183.

30. Knappskog S, Bjørnslett M, Myklebust LM, Huijts PEA, Vreeswijk MP, Edvardsen H, et al.. The MDM2 promoter SNP285C/309G haplotype diminishes Sp1 transcription factor binding and reduces risk for breast and ovarian cancer in Caucasians. *Cancer Cell*. 2011; doi: 10.1016/j.ccr.2010.12.019.

31. Knappskog S, Gansmo LB, Romundstad P, Bjørnslett M, Trovik J, Sommerfelt-Pettersen J, et al.. MDM2 promoter SNP344T>A (rs1196333) status does not affect cancer risk. *PLoS One*. 2012; doi: 10.1371/journal.pone.0036263.

32. Krueger F. Sherman - bisulfite-treated Read FastQ Simulator.

33. Harris RA, Raveendran M, Worley KC, Rogers J. Unusual sequence characteristics of human chromosome 19 are conserved across 11 nonhuman primates. *BMC Evol Biol*. 2020; doi: 10.1186/s12862-020-1595-9.

34. Krijthe JH. Rtsne: T-Distributed Stochastic Neighbor Embedding using Barnes-Hut Implementation.

35. Jaffe AE. FlowSorted.Blood.450k: Illumina HumanMethylation data on sorted blood cell populations.

36. Andrews SV, Bakulski KM. FlowSorted.CordBlood.450k: Illumina 450k data on sorted cord blood cells.

37. Poduval DB, Ognedal E, Sichmanova Z, Valen E, Iversen GT, Minsaas L, et al.. Assessment of tumor suppressor promoter methylation in healthy individuals. *Clin Epigenetics*. 2020; doi: 10.1186/s13148-020-00920-7.

38. Eikesdal HP, Yndestad S, Elzawahry A, Llop-Guevara A, Gilje B, Blix ES, et al.. Olaparib monotherapy as primary treatment in unselected triple negative breast cancer☆. *Annals of Oncology*. 2021; doi: 10.1016/j.annonc.2020.11.009.

39. Zhou L, Ng HK, Drautz-Moses DI, Schuster SC, Beck S, Kim C, et al.. Systematic evaluation of library preparation methods and sequencing platforms for high-throughput whole genome bisulfite sequencing. *Sci Rep*. 2019; doi: 10.1038/s41598-019-46875-5.

 Click here to access/download;Manuscript;response-to-reviewers.pdf

We thank the Reviewers for their constructive feedback. Below, we have provided comments to each of the Reviewers' questions and explained how we have revised the manuscript accordingly.

Reviewer #1:
The authors provide information and software (epialleleR) on a method to detect, quantify and visualize low-frequency hypermethylation events in NGS-based methylation data together with data showing the ability to link BRCA1 epimutations and cancer risk.

epialleleR is fully adjustable (parameter thresholds) to end-user needs. In the results there is shown, that the level of methylation per genomic position (Variant Epiallele Frequency (VEF)) is better suited for DMR (differentially methylated region) detection than FDR (false discovery rate) based methods.

For me, it seems uncommon to have the section "Material and Methods"at the end of the article, after the sections "Results", "Discussion" and "Conclusion"; but this is only a minor remark and is to authors discretion.

We do not have any preference for the order of sections. We see that many articles in the Journal have their "Materials and Methods" section at their end, but we would appreciate advice on this from the Editor and will of course change the order of sections if the Editor wishes.

I wonder, why runtime comparisons were executed in different environments (DRAGEN CX was different than Bismark, methylKit and epialleleR)? This makes it difficult to really compare efficiacy of packages. Maybe, the authors can add a statement on the reason for this.

We understand that this was unclear in the original manuscript. A practical problem for this comparison is that Illumina DRAGEN is a hardware solution that relies on the presence of FPGA accelerator card, which precludes DRAGEN software execution on other platforms. On the other hand, Illumina DRAGEN operating system (Red Hat Enterprise Linux 7 with GCC4.8, R3.6.0) is locked by vendor and quite outdated, which makes it very difficult to install recent development tools, as well as R environment and Bioconductor packages. Therefore, we had two use two different environments, although Bismark, methylKit and epialleleR were compared on the very same workstation. This explanation is now added in the "Processing speed comparison" paragraph in the Materials and Methods section of the revised manuscript.

Overall, I recommend to accept this article.

Reviewer #2: Review of GIGA-D-23-00149

The manuscript presents a computational approach for detecting what the authors term "mosaic epimutations". However, I found it very difficult to review the merits of the contribution owing to some fundamental issues with presentation, missing key details that motivate/justify the work, and problematic choices for evaluation/comparison. Below I list detailed concerns, separated by major and minor.

We have now provided additional clarifications in the text of the manuscript as well as in the comments below and we would be happy if Reviewer 2 will consider the manuscript again.

Major:

The manuscript lacks clarity in the presentation of the scientific question. The authors need to state their definition of mosaic methylation, and in addition how it relates to allele-specific methylation.

We agree that we could be more clear in these statements and in the revised version we consistently use the term “mosaic epimutations” instead of “mosaic methylation” in order to avoid confusion. In addition, we have provided simple statements defining the terminology in the text (e.g definition of “mosaic” in the introduction). In the Results section, we have also added a statement explaining the need for single-nucleotide variants to enable allele-specific methylation calls, clarifying how such allele-specific calls can be performed using epialleleR.

The motivation for development of new methodology as presented is somewhat contradictory. If the previously identified DNA methylation changes are so small (0.03-1%), and, as the authors state, "indistinguishable from the background methylation level", then how were they detected in the first place? Further, the analysis task is described by the authors as "trivial".

We understand that the order of events during the development of the methodology may be confusing. The fact is that we first discovered the qualitative presence of low-level mosaic epimutations in *BRCA1* by methylation specific qPCR and used this method for our initial case-control assessment (doi: 10.7326/M17-0101). However, given the low accuracy of qPCR in very high Cp-numbers (in the range of 38-42), we saw it as imperative to develop a high-depth NGS-based assay capable of delivering high sensitivity, methylation calls on individual CpGs as well as allele-specific information. Given the breakthrough findings regarding the biological relevance of low-level mosaic epimutations, we saw it as critical to the field to provide a method by which such epimutations could be detected and quantified reliably.

The specific contributions of this manuscript are unclear. The manuscript claims to present a computational framework, but also cites published work from the same authors that appears to have already used the framework (Lønning et al. 2022). If this is the case, this disclosure should be made more transparent.

The present manuscript was deposited and appeared online prior to publication of the Lønning et al., 2022 paper which cites this manuscript. While they are linked, this manuscript describes the computational method and its benefits as compared to other tools, and is aimed at bioinformaticians, while the subsequent Lønning et al., 2022 paper and another preprint (doi:10.1101/2023.05.14.23289949) describe experimental findings obtained by using this method and are aimed at clinical oncologists. In the revised manuscript, we have added this disclosure in the Declarations section. If this is a breach of journal style and the Editor would prefer us to move this statement somewhere else in the manuscript, we will of course do so.

Justification is needed for why "thresholding sequencing reads by their average methylation level will reduce the effect of biological and technical variance."

We realise that this was not properly explained in the original manuscript. To make the point clear in the revised manuscript, we have provided an extended explanation, replacing the previous sentence. It now reads as follows: “As number of events that lead to variance in methylation (base deamination, random single-base methylation events and sequencing errors) is limited at the level of individual reads (only a fraction of CpGs might be affected within the same read), the average methylation level of the read will be moderately affected by such events and can help distinguish hyper- from hypomethylated epialleles. We therefore hypothesized that thresholding sequence reads

by their average methylation level will reduce the effect of biological and technical variance and facilitate the detection of infrequent hypermethylation events". Notably, as we showed by sensitivity analyses, this hypothesis was correct.

Justification and motivation for the VEF metric is not provided, nor are all details provided on how it's calculated. I tried to follow the description for individual cytosines, but am immediately stuck on how to define Calpha for cytosine i. Calpha is described as the number of methylated cytosines in read pairs passing the threshold - it's not clear what is being counted over. Presumably this should only be considering reads that overlap that particular cytosine i, and perhaps it's counting over all cytosines in all such reads, but this is counting multiple times per cytosine (since more than 1 read can overlap a cytosine), so this seems to contradict the description. Moreover, if I just take the description at face value as "epialleles with similar methylation properties that is defined by thresholding", it's not clear what this represents biologically. It seems to intend to measure variability of methylation.

We understand that VEF is a newly introduced metric and that it should be explained in detail. Instead of providing more text, we have now included a panel B in Figure 1, where we graphically explain how VEF values are calculated at the level of individual bases. We believe that this figure panel, with its accompanying legend, will make the underlying calculations of VEF more easily accessible for the reader.

In addition, the extensive online documentation to which we point our users (at https://bbcg.github.io/epialleleR/articles/epialleleR.html or https://bioconductor.org/packages/devel/bioc/vignettes/epialleleR/inst/doc/epialleleR.html) provides further clarifications and scenarios for specific analyses.

It's not clear how VEF metrics compare to established measures of methylation variability and/or allelic variation of methylation (e.g. Variably Methylated Region identification, methHaplo, methtuple, DAMEfinder). There are alternatives discussed in terms of entropy and discordant reads, but they are not compared in the sensitivity analysis study of WBC.

Variably methylated regions (VMR), by definition, are the regions showing variable methylation levels between samples. Such regions are not what epialleleR is designed to quantify. Unlike VMR tools, epialleleR outputs per-base value (VEF) that can significantly boost sensitivity of aberrant or differentially methylated region detection tools for the detection of mosaic epimutations (epimutations present in 1 out of >100 cells). This is presented in the sensitivity analyses that employ the best to our knowledge DMR analysis tool (DMRcate).

We agree with the Reviewer that it is always a good practice to compare to established tools. However, the tools suggested by Reviewer (methHaplo, methtuple, DAMEfinder) actually have different purposes than epialleleR. They are used to assemble methylation haplotypes (methHaplo), detect changes in allele-specific methylation between two groups of samples (DAMEfinder), or count m-tuples in reads (methtuple). While they use BAM files as an input, none of them is able to output a metric which is directly comparable to VEF (per-CpG methylation level) or its purpose (to count concordant hypermethylation events, i.e., epimutations). For example, methHaplo specifically excludes CpGs with low methylation level (beta<0.1) as they are not informative for its purpose. In contrast, epialleleR is designed to detect and quantify rare epimutations, can extract all epialleles and can use optional VCF data to correlate SNVs with methylation – this was used by us to discover prenatal nature of *BRCA1* epimutations that lead to cancer in adulthood. Besides, epialleleR can itself call methylation and use BAM files produced by virtually any aligners (which is not the case for tools mentioned above).

The other metrics (entropy, PDR, etc) are not per-base metrics and cannot be used for differential/aberrant methylation analysis.

As such, we feel it would leave the readers with a misleading impression about the tools and their purpose if we tried to present any comparison, and we have therefore not done this in the sensitivity analysis section.

A major limitation of the interpretation of so-called epialleles is the inability to distinguish allelic variation from cell type heterogeneity. This is acknowledged very briefly and very late in the manuscript. This needs to be stated up front. In addition, a discussion of potential remedies (if any) would be helpful.

We agree that cell type heterogeneity is a factor that must be taken into consideration in methylation analyses in general. But, while cell type heterogeneity can explain some of the methylation differences in other studies, the phenomenon we have described here does not depend on it. Only 1 CpG in this study and none of CpGs analysed in Lønning et al., 2022 have significantly different methylation levels between blood cell types. Regarding other regions, it is important to note that our method, epialleleR, is not itself biased by cell type heterogeneity because it is executed upstream of the data interpretation. epialleleR reports frequencies of individual epialleles or their groups (by methylation), while it is the downstream analysis (comparison of samples or their groups) which should account for any confounding factor, including, but not limited to, cell type heterogeneity. Given the possibility to perform allele-specific methylation calls with epialleleR, we actually believe it will be a useful addition to tools that may be used to address cell type heterogeneity, since methylation on different alleles in different tissues will indicate different methylation events to have occurred in different tissues of the same individual.

The simulated data are very unrealistic, which precludes interpretation of results. It seems that all CpGs were simulated with the same methylation level of 50%, and all other cyosines at 0.25% methylation levels. More importantly, introducing random sequencing errors is unrealistic, as per base sequence quality is known to be generally lower in read 2 compared to read 1. Further, there's a known increase in read 2 of M bias in bisulfite sequencing. With a more realistic error generating process, I expect choosing base with highest quality to be effectively the same as trimming read 2, meaning the results presented here are an artifact of simulation choices.

The purpose of the simulated data is to have a known level of methylation of *all* cytosines, so that the reported values can be compared to the expected ones. While the ground truth was chosen to be exactly 50% methylation in CG context and approximately 0.25% in CHG and CHH ones, this does not undermine the results of the comparison. We believe, the values applied are not relevant per se; they could have been 5% or 95% or anything else. The tools are still compared based on the lowest difference of observed (reported) values to the expected ones.

The Sherman bisulfite read simulator used by us here is a state-of-the-art tool, which uses the most realistic simulation strategy available. The errors are not entirely random but introduced according to exponential decay model (derived from real data). Simulated per-base quality scores follow the same curve as well. As pointed out by the Reviewer, it is indeed known that read 2 contains more base substitutions than read 1. However, the number of sequencing errors depends strongly on a sequence context and read length, therefore errors are present in both read 1 and read 2, and are more abundant at their ends. It is a quite common situation that a lower-quality 3' end of read 1 overlaps with higher-quality region of read 2 (either middle or its 5'). A simple trimming of a higher-quality part of read 2 will then result in lower-quality bases retained. This is why the base quality-driven merging will naturally result in fewer errors and we therefore consider it to be favourable.

The subtle increase in reporting accuracy is not very valuable on its own, but together with the vast speed improvement it allows us to recommend our method for virtually every analysis of methylation sequencing BAM files when methylation needs to be called at individual cytosine

residues.

Methods are not described in enough detail to reproduce results. For example, the details on usage of Bismark in the accuracy simulation is not included (only for speed comparison).

The methylation reporting for accuracy comparison was done exactly as described for the speed comparison for all tools. For clarity, this has now been specified in the "Cytosine reporting accuracy comparison" paragraph in Materials and Methods of the revised manuscript.

Owing to the major concerns listed thus far, I am not able to fully review the "Sensitivity analyses" section at this time.

We hope that the revisions in the manuscript text and the comments made here in our response letter, are clarifying and that all parts of the manuscript are now easily accessible for the reader.

Minor:

Organization of manuscript could use improvement (e.g. breaking up full page+ paragraphs, making sure headers accurately reflect section content).

We are uncertain of which specific paragraphs and/or subheadings the Reviewer would recommend to edit. Going through the manuscript, we have found that some of our previous subheadings were not fully precise covering the contents of the following paragraphs. Therefore, we have made some minor changes for better concordance. Thus, new subheadings in the revised manuscripts are "Reporting accuracy analyses" in the Results section and "Processing speed comparison" in the Material and Methods section. In addition, some very long paragraphs were broken into smaller paragraphs.

epialleleR is marked in Table 1 as not requiring a reference genome. But isn't it needed to obtain the bam file? If not, then how was the bam file generated for use with epiallelR? It's not a fair comparison to mark Bismark as needing a reference genome (to carry out the bam file generation step), as far as I'm aware this isn't used in the methylation extractor step in Bismark (the input is a BAM/SAM file).

Bismark methylation extractor indeed requires reference genome in addition to BAM input. For the methylation reporting, epialleleR does not require reference genome (similar to methylKit, but unlike methylation reporting by DRAGEN or Bismark).

In table 2, 'mean methylation level' is not generally considered an accuracy metric. Some metric of difference from ground truth should be shown.

We agree with the Reviewer that "mean methylation level" is indeed not an accuracy metric. Therefore, we provided "variance" as a measure of the differences between observed and expected values. Both these values allow the reader to see what tool reports closest to expected methylation values (0.5 and 0.0025 for cytosines in CG and CHG/CHH contexts, respectively). In order to clarify this issue, we have changed the title of the table, so that it now additionally includes these expected values.

When the authors state "Concordantly methylated alleles (genomic regions with spatially extended / haplotype-specific methylation)", I think they just mean allele-specific methylation. If not, please clarify. The other terms used (concordant methylation or spatially extended

methylation) are nonstandard, and, puzzlingly, not mentioned in the citations provided for this sentence, where I searched for additional context.

By concordantly methylated alleles we mean reads with concordant methylation of cytosines (e.g., most of CpGs either methylated or unmethylated). We agree that this was unclear in our original manuscript and have now replaced the sentence with a simple explanation for "Concordantly methylated alleles" in the first sentence of the "Sensitivity analyses" paragraph of the Results section.

In Figure 2A what region is shown and how was this chosen? It appears the beta values are shown on a log scale, which is a nonstandard choice. M-values would be advisable to check as well.

Figure 2A shows all 138 CpGs included in the sensitivity analyses, with their genomic position as a categorical variable along x-axis. We understand that this was unclear and have now included an additional explanation in the figure legend.

Data are presented using log scale to illustrate exponential (3-fold) differences between cytosine methylation levels (1e-4 to 1e-2) between all samples. This would not be possible using a linear scale and we have therefore chosen to keep the log scale in the figure.

As epialleleR cytosine reports are similar to Bismark cytosine reports in that epialleleR outputs both numbers of methylated ("C^a" with thresholding or "C" without thresholding) and unmethylated ("T") cytosines per genomic position, the package users can utilize those counts in any downstream analyses in the form of a beta value or M-value. Here, we chose to present beta/VEF values only, as sequencing data is compared with methylation array data, for which M-values are not available.

In Figure 2D/E, a measure of specificity should be reported as well.

The experimental setup allowed us to extract only the total number of real positive regions (P), the number of true positive regions (TP), and the number of false negative regions (FN=P–TP) for every analysis, while the numbers of true negative (TN) or false positive (FP) regions were undefined. Therefore, recall, or true positive rate (TPR=TP/P) was chosen as a sensitivity metric. This is described in Materials and Methods and the TPRs are now presented in the revised manuscript.

Should state much earlier in the manuscript which types of methylation sequencing data are suitable for this approach.

We agree that it would be beneficial to make an early statement about this and have now included this earlier in the text (Introduction and epialleleR implementation sections).

--

Figure 1

Click here to access/download;Figure;Fig1.pdf

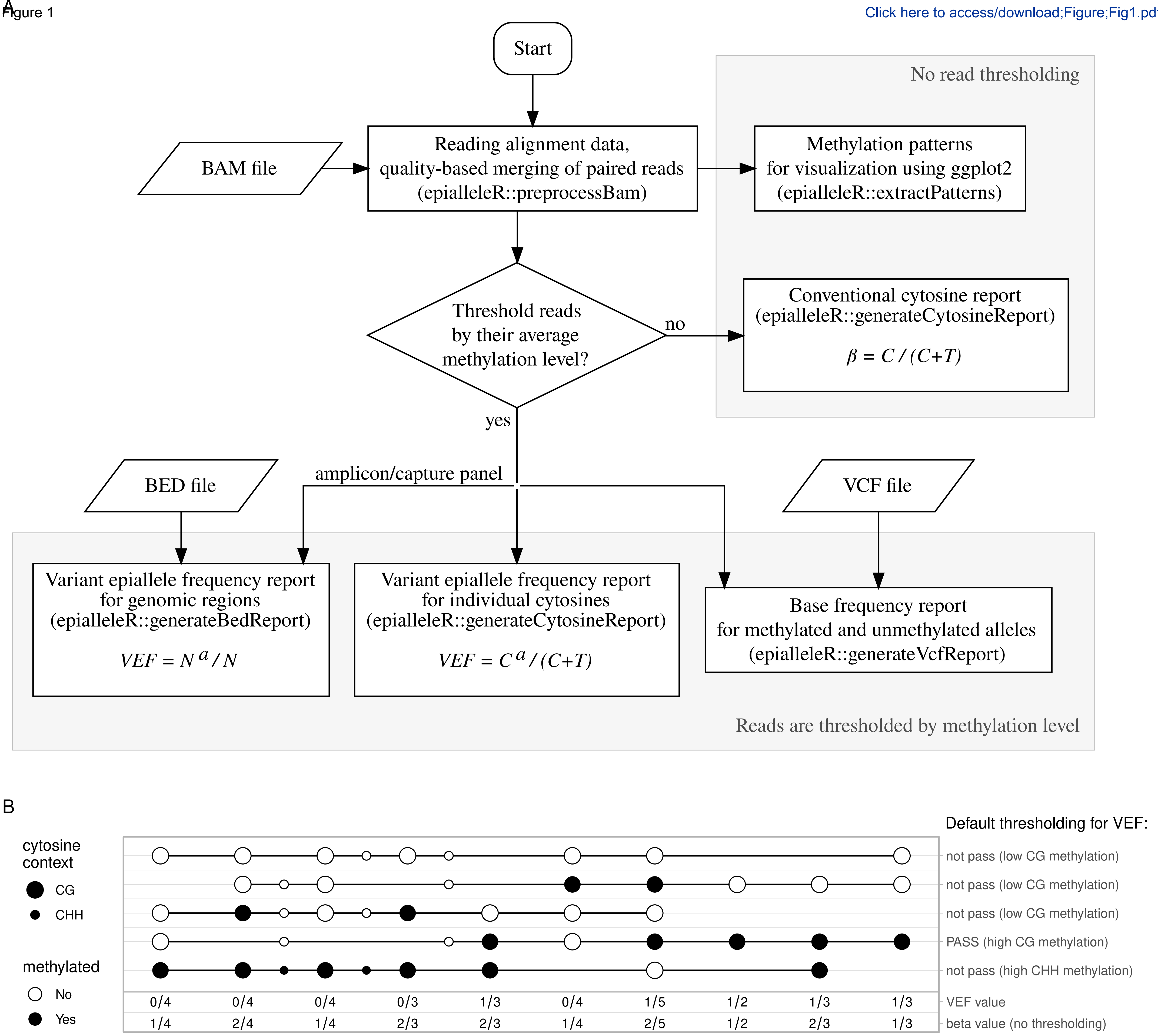

Figure 2

Click here to access/download;Figure;Fig2.pdf

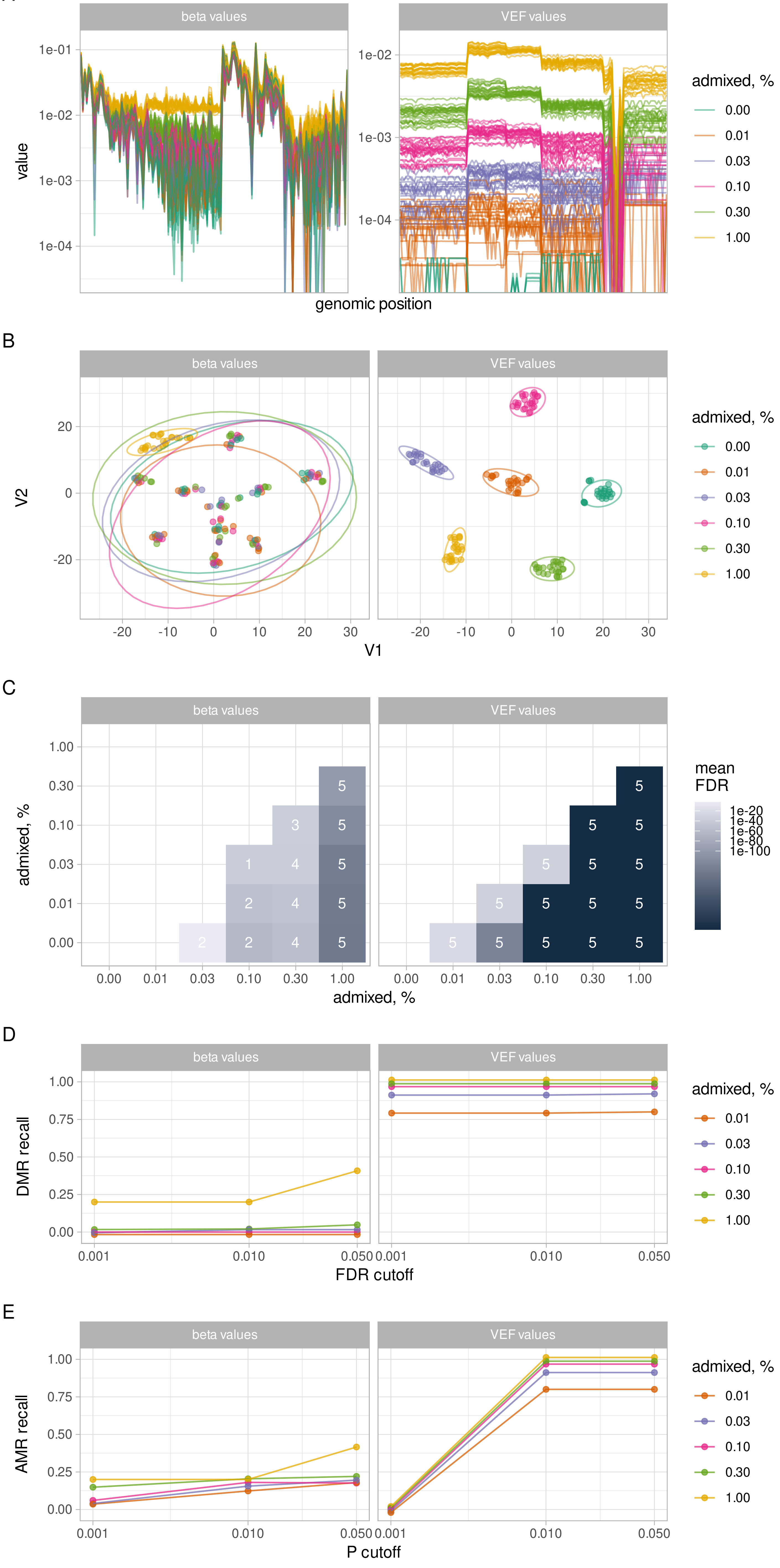

Figure 3

Click here to access/download;Figure;Fig3.pdf

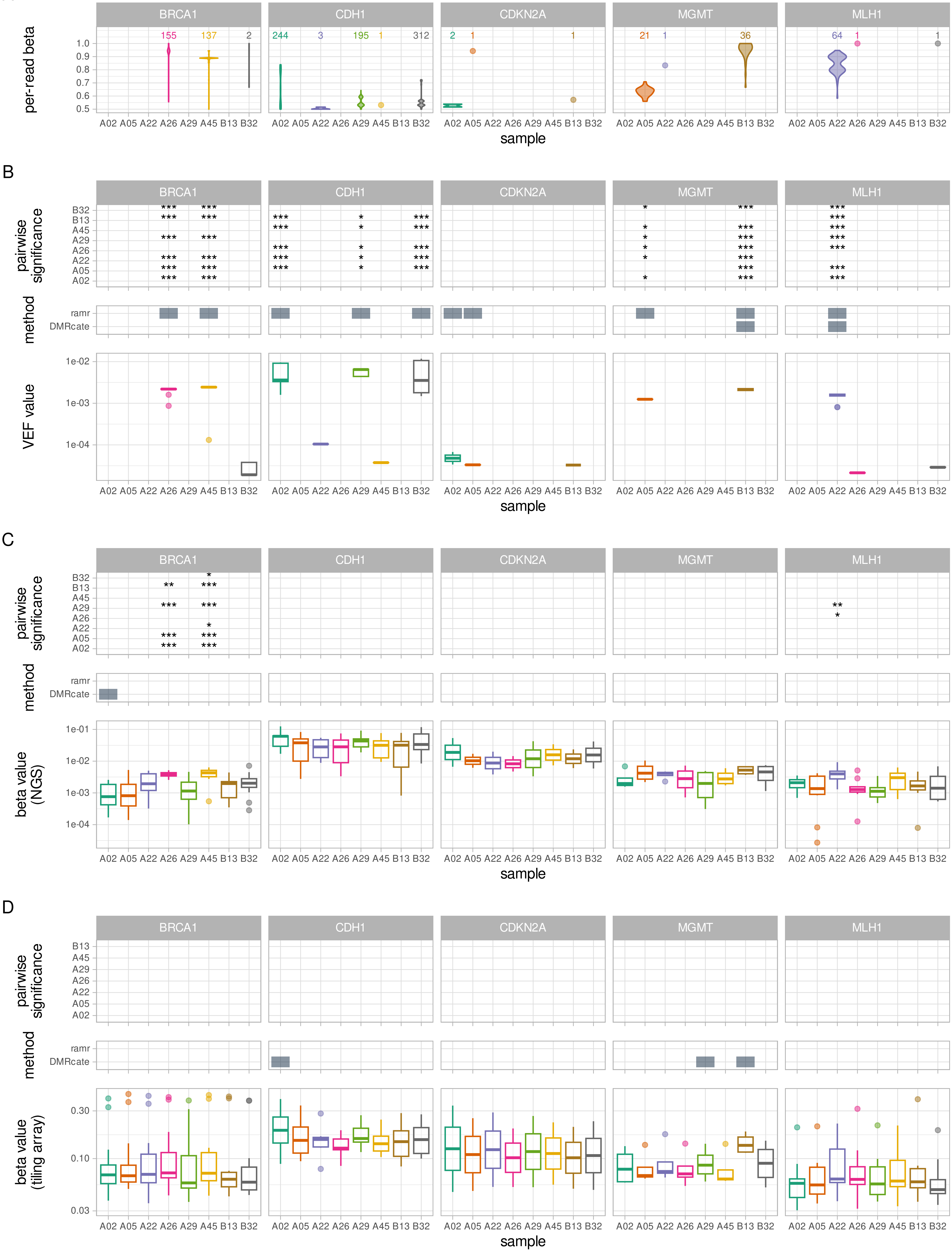

Figure 4

Click here to access/download;Figure;Fig4.pdf

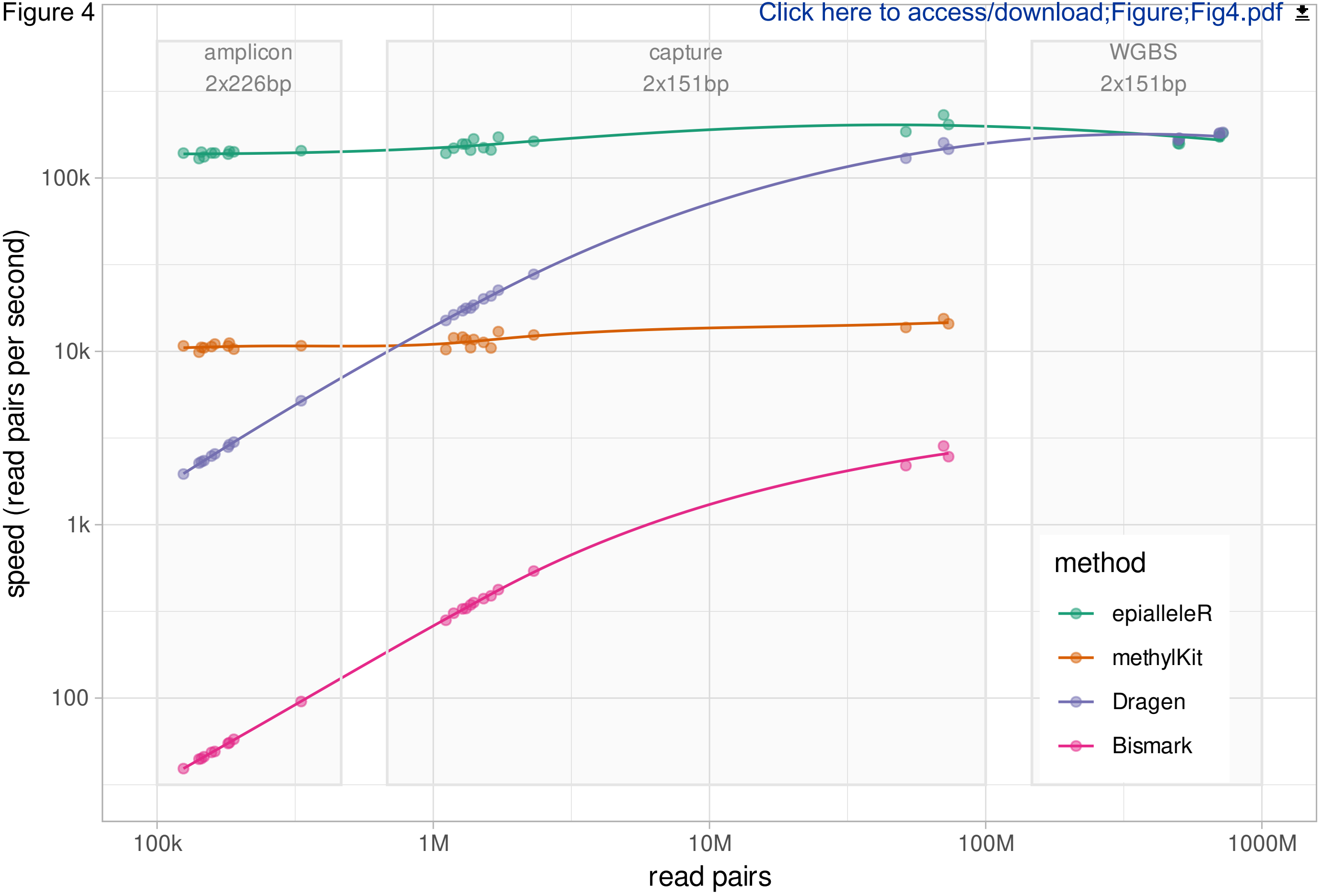

Click here to access/download
Supplementary Material
Supplementary_Material.pdf

Supplementary Table 1

Click here to access/download
Supplementary Material
Supplementary_Table_1.xlsx
