# Supplementary material for: epialleleR: an R/Bioconductor package for sensitive allele-specific methylation analysis in NGS data
Source: Gigascience. 2023 Oct 31;12:giad087. doi: 10.1093/gigascience/giad087 (PMC10622323; doi:10.1093/gigascience/giad087)
Supplement: giad087_Supplemental_Files [file giad087_supplemental_files.zip › Supplementary_Material.pdf]

Supplementary Table 1. Complete metrics of cytosine reporting across all three possible cytosine genomic contexts (CHH, CHG and CpG), obtained using simulated chr19 reads with varying sequencing error rate by selected tools. “reported”, number of cytosines present in the cytosine reports; “valid context”, number of cytosines for which genomic context was correctly identified; “invalid context”, number of cytosines for which genomic context was incorrectly identified; “not covered”, number of cytosines not present in a cytosine report; “mean coverage”, average coverage of all cytosines in this context; “mean” and “variance”, average value and variance for beta values of all cytosines in this context; “is 0.5”, number of cytosines with beta value of exactly 0.5 (ground truth for this dataset); “is not 0.5”, number cytosines with beta value not equal to 0.5.

Supplementary Fig. 1. Scaled density of per-read beta values from all admixed samples combined, split by the level of admixed reads and genomic region of interest. The y axis is scaled to 1 and limited to 0.015. The hypermethylated ($\beta \geq 0.5$) reads are increasingly apparent on the right sides of plots in accordance with an increase in admixed methylated reads.

Supplementary Fig. 2. Methylation patterns from all admixed samples combined, split by the level of admixed reads and genomic region of interest. Lines depict patterns, open and closed circles depict unmethylated and methylated cytosines, respectively. Numbers on the right of every pattern indicate how many times each pattern occurs for every given gene/sample set. Due to very high number of methylation pattern types, only the most abundant pattern (if any) is shown for each range of average beta value: [0,0.2), [0.2,0.4), [0.4,0.6), [0.6,0.8), [0.8,1]. The hypermethylated ($\beta \geq 0.5$) patterns are increasingly apparent at the top of plots in accordance with an increase in admixed methylated reads.

Supplementary Fig. 3. Scaled density of per-read beta values from n=8 real samples used to compare sensitivity of methylation profiling by NGS and array, split by sample and genomic region of interest. The y axis is scaled to 1 and limited to 0.015. The population of hypermethylated ($\beta \geq 0.5$) reads that are apparent on the Fig. 3A in the main text are pointed to by black arrows.

Supplementary Fig. 4. Methylation patterns from n=8 real samples used to compare sensitivity of methylation profiling by NGS and array, split by sample and genomic region of interest. Lines depict patterns, open and closed circles depict unmethylated and methylated cytosines, respectively. Numbers on the right of every pattern indicate how many times each pattern occurs for every given gene/sample set. Due to very high number of methylation pattern types, only the most abundant pattern (if any) is shown for each range of average beta value: [0,0.2), [0.2,0.4), [0.4,0.6), [0.6,0.8), [0.8,1]. The hypermethylated ($\beta \geq 0.5$) patterns represent hypermethylated epialleles that are present in certain samples/regions, as shown by the Fig. 3A in the main text and Supplementary Fig. 3.

Supplementary Fig. 5. (A) Distribution of per-read beta values for NGS reads covering CpGs that are common for NGS and BeadChip array. For clarity, only the reads with average beta of at least 0.5 (i.e., representing hypermethylated epialleles) are included. Single observations are shown as dots, number of observations is given above. (B) Lower panel: box plots of NGS-derived VEF values for individual CpGs; upper panel: significant aberrantly or differentially methylated regions identified by ramr or DMRcate, respectively, based on VEF values. (C) Lower panel: box plots of NGS-derived beta values for individual CpGs; upper panel: significant aberrantly or differentially methylated regions identified by ramr or DMRcate, respectively, based on NGS-derived beta values. (B–C) The lower and upper hinges of boxes correspond to the first (Q1) and third (Q3) quartiles; the bar in the middle correspond to the median value; the upper and lower whisker extend to Q3+1.5*IQR and Q1-1.5*IQR, respectively, while the values outside this range (outliers) are plotted as dots. Zero values are not plotted. The colouring is preserved for n=8 samples used in Fig. 3 of the main text. The n=10 samples used to create admixed samples and not included in Fig. 3 are plotted in light grey.

Supplementary Fig. 6. (A) Heatmap of minimum (equals absolute largest) difference in combinatorial entropy for all pairs of samples, split by genomic region of interest. (B) Heatmap of combinatorial entropy, epipolymorphism, fraction of discordant read pairs (FDRP) and proportion of discordant reads (PDR), split by genomic region of interest. (C) Heatmap of p values for pairwise comparison of samples using WSH scores, split by score and genomic region of interest. *** $p<0.001$, ** $p<0.01$, * $p<0.05$, blank $p \geq 0.05$

Supplementary Table 1

sequencing error rate	method	CHH						CHG						CpG							
		reported		not covered	mean coverage	beta values		reported		not covered	mean coverage	beta values		reported		not covered	mean coverage	beta values			
		valid context	invalid context			mean	variance	valid context	invalid context			mean	variance	valid context	invalid context			is 0.5	is not 0.5	mean	variance
0.00%	dragen	19154385	0	56187	203.68	**0.002501**	1.28E-05	6573937	0	19963	203.80	**0.002500**	1.27E-05	2200279	0	10961	203.25	2189866	10413	0.499997	5.96E-07
	bismark	19154371	0	56201	194.96	0.002501	1.34E-05	6573935	0	19965	195.07	0.002500	1.33E-05	2200275	0	10965	194.56	**2191308**	**8967**	**0.499997**	**5.92E-07**
	methylkit	19154385	0	56187	204.07	0.002503	**1.28E-05**	6573937	0	19963	204.19	0.002501	**1.27E-05**	2200279	0	10961	203.64	2189867	10412	0.499997	5.97E-07
	epialleleR	19154385	0	56187	203.68	**0.002501**	1.28E-05	6573937	0	19963	203.80	**0.002500**	1.27E-05	2200279	0	10961	203.25	2189866	10413	0.499997	5.96E-07
0.10%	dragen	19154394	0	56178	203.54	0.002661	1.37E-05	6573909	0	19991	203.66	0.002662	1.36E-05	2200248	0	10992	203.17	2116830	83418	0.499835	1.73E-06
	bismark	19154378	0	56194	194.83	0.002646	1.42E-05	6573904	0	19996	194.95	0.002648	1.41E-05	2200248	0	10992	194.49	2127434	72814	0.499850	1.69E-06
	methylkit	19154394	156	56178	203.92	0.002662	1.37E-05	6573909	116	19991	204.05	0.002664	1.36E-05	2200248	107	10992	203.55	2116536	83712	0.499835	1.75E-06
	epialleleR	19154393	0	56179	203.57	**0.002624**	**1.35E-05**	6573909	0	19991	203.69	**0.002626**	**1.34E-05**	2200248	0	10992	203.20	**2132685**	**67563**	**0.499872**	**1.54E-06**
0.30%	dragen	19154462	0	56110	203.24	0.002977	1.53E-05	6573961	0	19939	203.34	0.002980	1.53E-05	2200275	0	10965	202.80	1978460	221815	0.499504	3.22E-06
	bismark	19154457	0	56115	194.58	0.002928	1.57E-05	6573957	0	19943	194.67	0.002931	1.57E-05	2200273	0	10967	194.16	2007647	192626	0.499554	3.03E-06
	methylkit	19154462	1849	56110	203.63	0.002978	1.52E-05	6573961	1639	19939	203.72	0.002982	1.52E-05	2200275	1547	10965	203.18	1978911	221364	0.499506	3.21E-06
	epialleleR	19154459	0	56113	203.34	**0.002857**	**1.47E-05**	6573960	0	19940	203.43	**0.002860**	**1.47E-05**	2200275	0	10965	202.90	**2028765**	**171510**	**0.499628**	**2.59E-06**
0.60%	dragen	19154927	0	55645	202.79	0.003498	1.79E-05	6573979	0	19921	202.90	0.003506	1.78E-05	2200281	0	10959	202.36	1763714	436567	0.498942	1.29E-05
	bismark	19154901	0	55671	194.19	0.003393	1.81E-05	6573971	0	19929	194.30	0.003402	1.81E-05	2200279	0	10961	193.79	1819202	381077	0.499051	1.25E-05
	methylkit	19154927	9913	55645	203.17	0.003497	1.78E-05	6573979	8730	19921	203.28	0.003501	1.78E-05	2200281	8115	10959	202.74	1767002	433279	0.498952	1.28E-05
	epialleleR	19154923	0	55649	202.99	**0.003237**	**1.66E-05**	6573976	0	19924	203.11	**0.003241**	**1.65E-05**	2200279	0	10961	202.56	**1866484**	**333795**	**0.499222**	**1.14E-05**

Supplementary Figure 1. Scaled density of per-read beta values in admixed samples

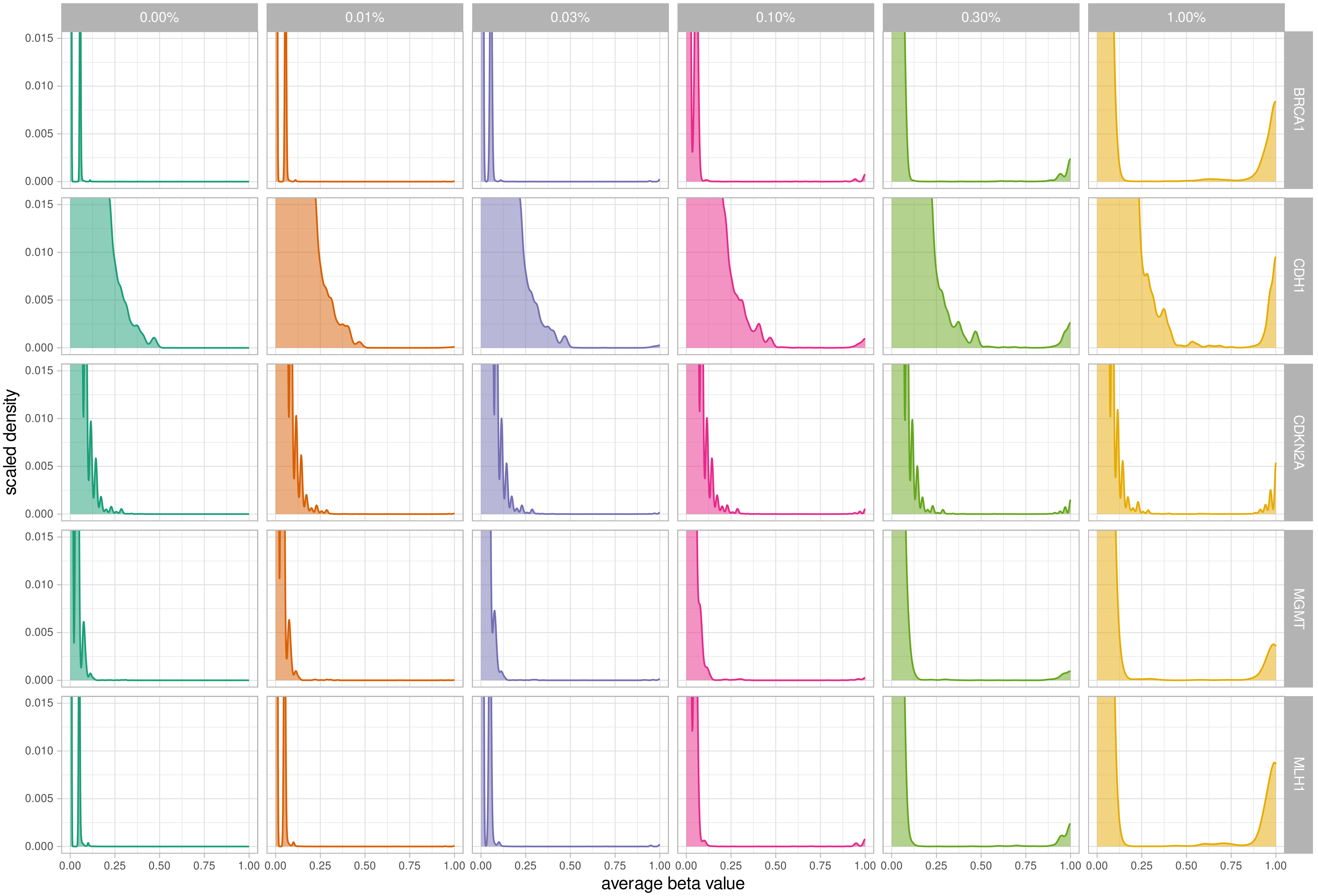

Supplementary Figure 2. Epialleles in admixed samples

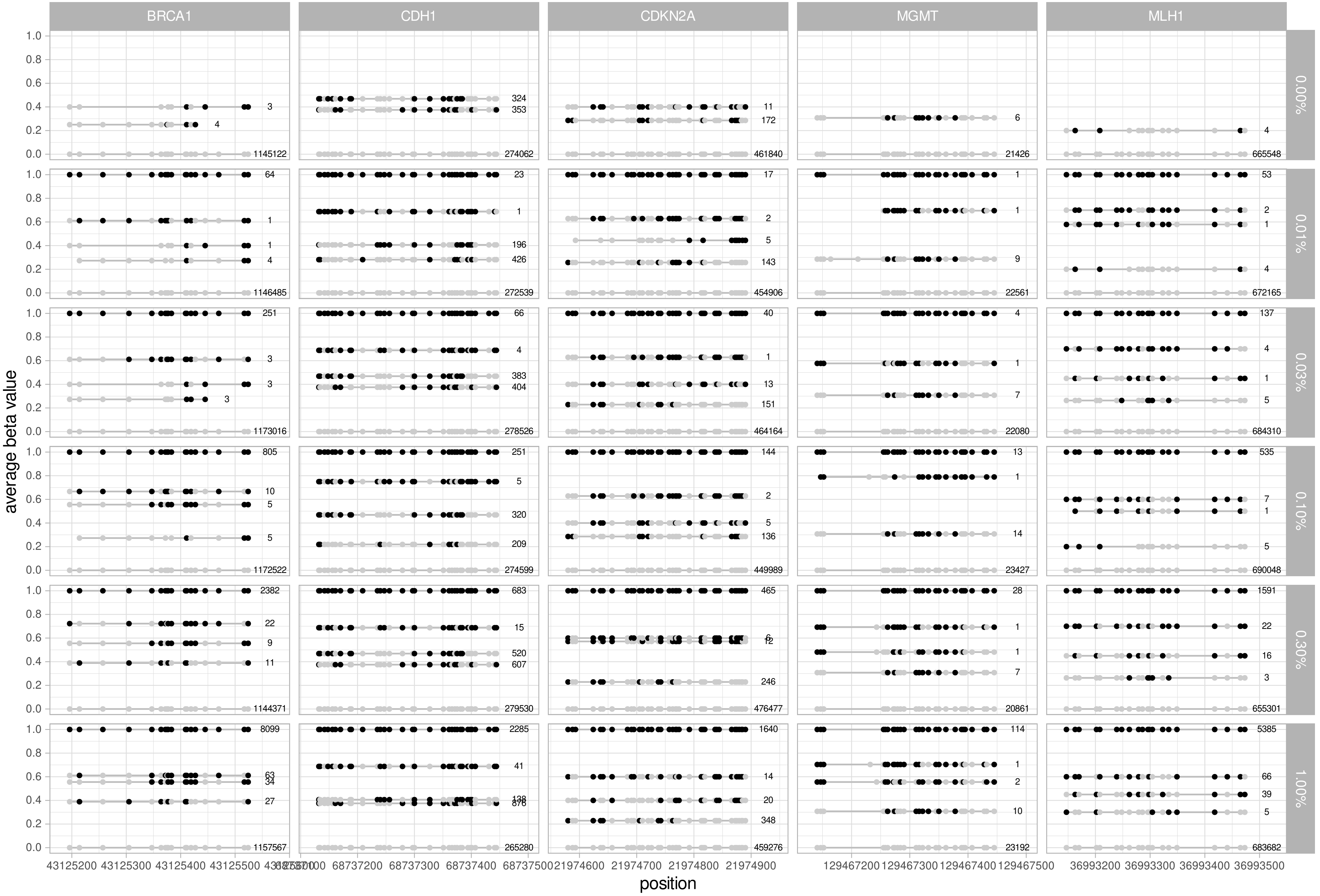

Supplementary Figure 3. Scaled density of per-read beta values in real samples

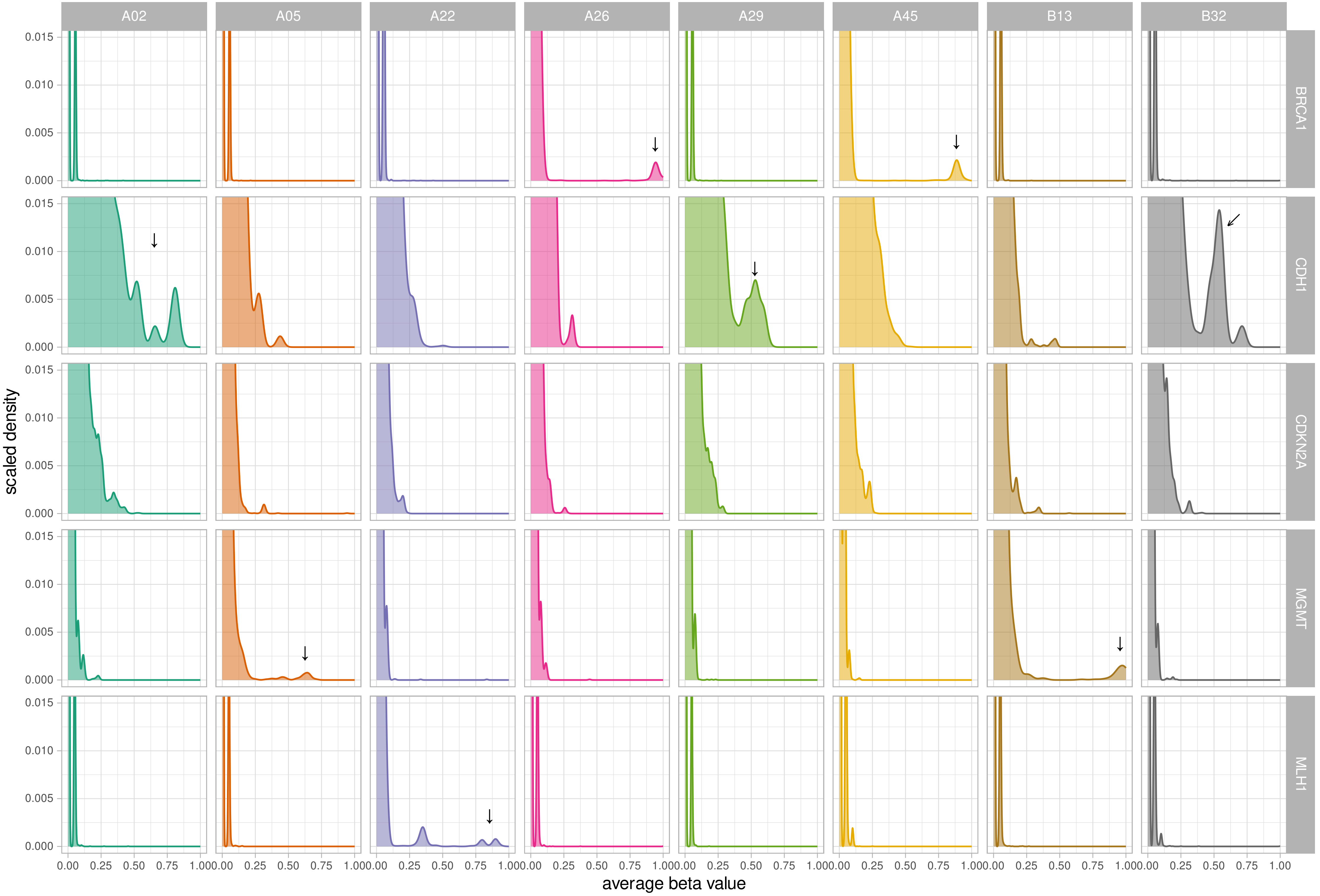

Supplementary Figure 4. Epialleles in real samples

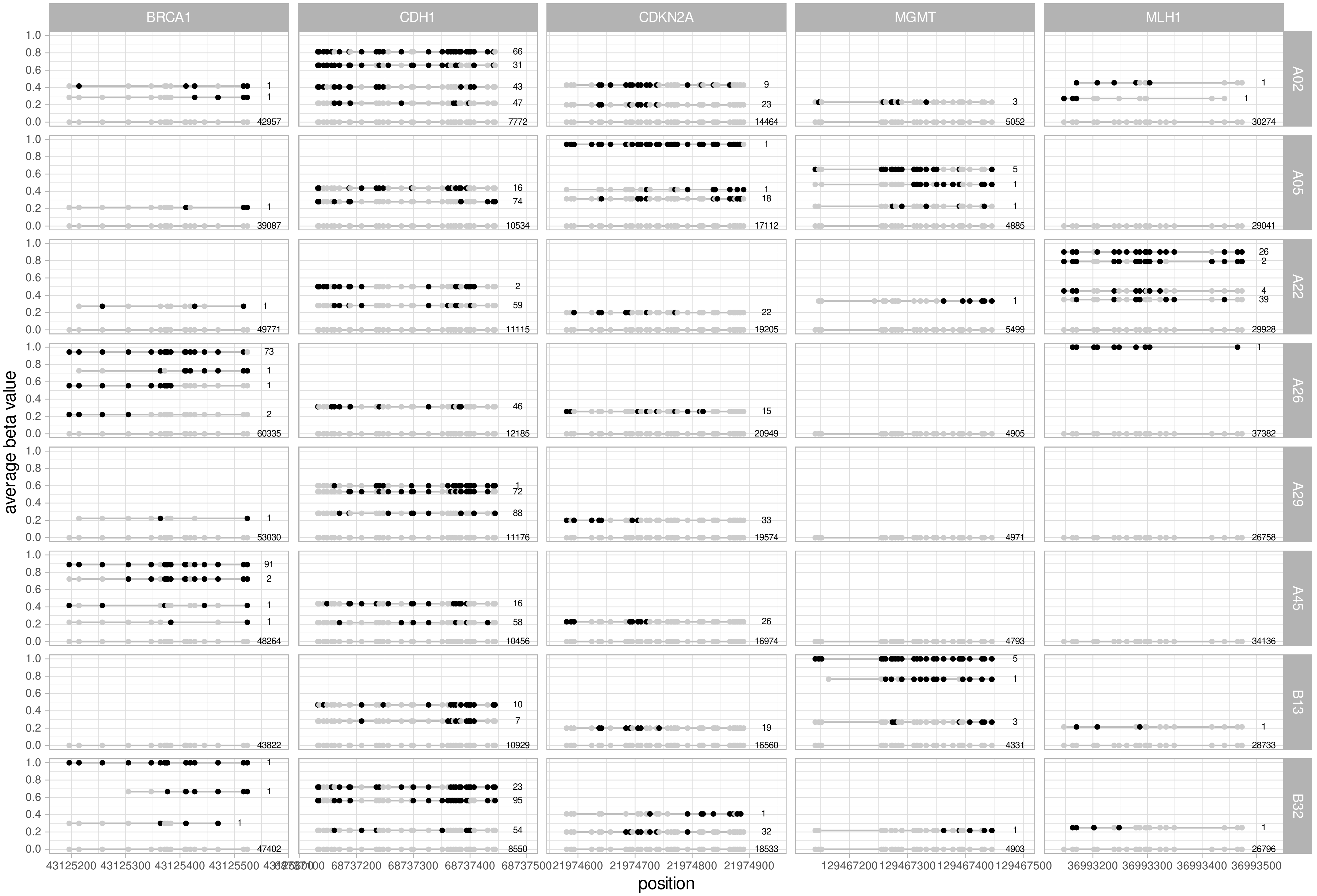

Supplementary Figure 5

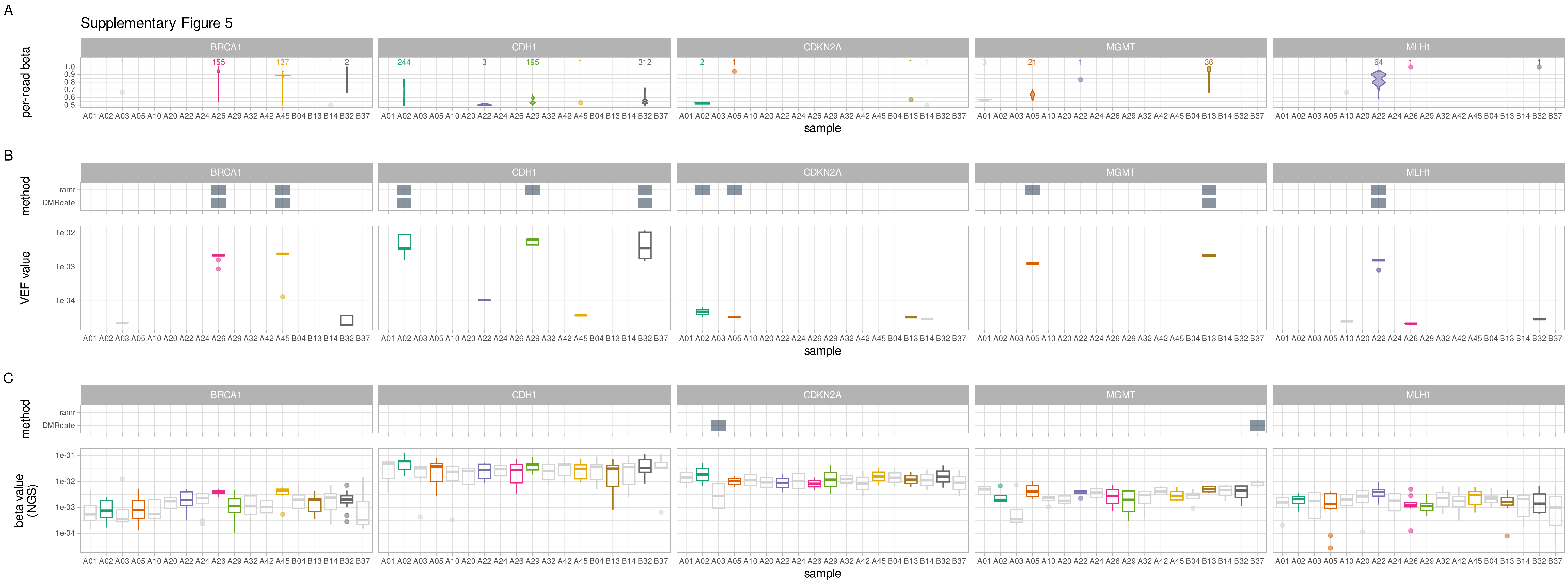

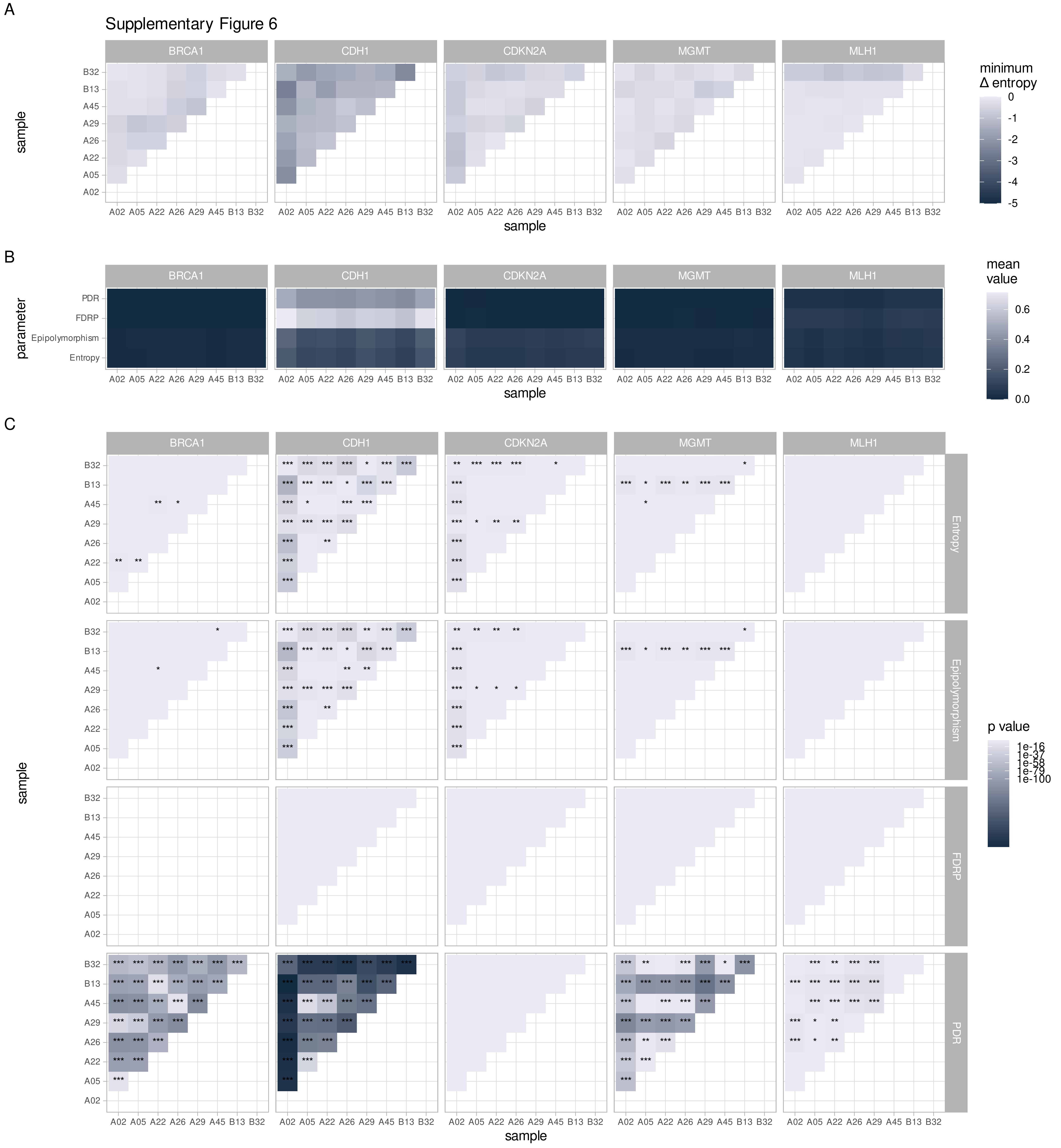
A
Supplementary Figure 6
BRCA1
CDH1
CDKN2A
MGMT
MLH1
sample
B32
B13
A45
A29
A26
A22
A05
A02
minimum
Δ entropy
0
-1
-2
-3
-4
-5
B
PDR
FDRP
Epipolymorphism
Entropy
parameter
mean
value
0.6
0.4
0.2
0.0
C
p value
1e-16
1e-37
1e-58
1e-79
1e-100
Entropy
Epipolymorphism
FDRP
PDR
