# Supplementary material for: epialleleR: an R/Bioconductor package for sensitive allele-specific methylation analysis in NGS data
Source: Gigascience. 2023 Oct 31;12:giad087. doi: 10.1093/gigascience/giad087 (PMC10622323; doi:10.1093/gigascience/giad087)
Supplement: giad087_Response_to_Reviewer_Comments_Original_Submission [file giad087_response_to_reviewer_comments_original_submission.pdf]

Author's Response To Reviewer Comments

Dear Dr. Nogoy,

Thank you for the thorough consideration of our manuscript GIGA-D-23-00149 and for giving us the opportunity to submit a revised version of the manuscript.

We find the comments from Reviewers constructive and useful. In particular, we found that the majority of the comments from Reviewer 2 were due to our phrases, that we realize were imprecise, at different places in the original manuscript. By addressing these comments, we feel we have made the manuscript much clearer for the readers and that there should be no room for any misunderstandings.

All changes are marked in the revised document and a point-by-point explanation for our revisions according to each of the Reviewers' comments is additionally attached in the document "Response-to-reviewers.pdf".

In addition to the revisions, we have also registered epialleleR at bio.tools and Research Resource Identification Portal and the relevant IDs are now included in the availability section of the manuscript.

We hope the revised version of our manuscript will be suitable for publication in GigaScience and look forward to your response in due time.

On behalf of the authors,
Oleksii Nikolaienko, PhD

We thank the Reviewers for their constructive feedback. Below, we have provided comments to each of the Reviewers' questions and explained how we have revised the manuscript accordingly.

Reviewer #1:
The authors provide information and software (epialleleR) on a method to detect, quantify and visualize low-frequency hypermethylation events in NGS-based methylation data together with data showing the ability to link BRCA1 epimutations and cancer risk.

epialleleR is fully adjustable (parameter thresholds) to end-user needs. In the results there is shown, that the level of methylation per genomic position (Variant Epiallele Frequency (VEF)) is better suited for DMR (differentially methylated region) detection than FDR (false discovery rate) based methods.

For me, it seems uncommon to have the section "Material and Methods"at the end of the article, after the sections "Results", "Discussion" and "Conclusion"; but this is only a minor remark and is to authors discretion.

A: We do not have any preference for the order of sections. We see that many articles in the Journal have their "Materials and Methods" section at their end, but we would appreciate advice on this from the Editor and will of course change the order of sections if the Editor wishes.

I wonder, why runtime comparisons were executed in different environments (DRAGEN CX was different than Bismark, methylKit and epialleleR)? This makes it difficult to really compare efficiacy of packages.

Maybe, the authors can add a statement on the reason for this.

A: We understand that this was unclear in the original manuscript. A practical problem for this comparison is that Illumina DRAGEN is a hardware solution that relies on the presence of FPGA accelerator card, which precludes DRAGEN software execution on other platforms. On the other hand, Illumina DRAGEN operating system (Red Hat Enterprise Linux 7 with GCC4.8, R3.6.0) is locked by vendor and quite outdated, which makes it very difficult to install recent development tools, as well as R environment and Bioconductor packages. Therefore, we had two use two different environments, although Bismark, methylKit and epialleleR were compared on the very same workstation. This explanation is now added in the "Processing speed comparison" paragraph in the Materials and Methods section of the revised manuscript.

Overall, I recommend to accept this article.

Reviewer #2: Review of GIGA-D-23-00149

The manuscript presents a computational approach for detecting what the authors term "mosaic epimutations". However, I found it very difficult to review the merits of the contribution owing to some fundamental issues with presentation, missing key details that motivate/justify the work, and problematic choices for evaluation/comparison. Below I list detailed concerns, separated by major and minor.

A: We have now provided additional clarifications in the text of the manuscript as well as in the comments below and we would be happy if Reviewer 2 will consider the manuscript again.

Major:

The manuscript lacks clarity in the presentation of the scientific question. The authors need to state their definition of mosaic methylation, and in addition how it relates to allele-specific methylation.

A: We agree that we could be more clear in these statements and in the revised version we consistently use the term "mosaic epimutations" instead of "mosaic methylation" in order to avoid confusion. In addition, we have provided simple statements defining the terminology in the text (e.g definition of "mosaic" in the introduction). In the Results section, we have also added a statement explaining the need for single-nucleotide variants to enable allele-specific methylation calls, clarifying how such allele-specific calls can be performed using epialleleR.

The motivation for development of new methodology as presented is somewhat contradictory. If the previously identified DNA methylation changes are so small (0.03-1%), and, as the authors state, "indistinguishable from the background methylation level", then how were they detected in the first place? Further, the analysis task is described by the authors as "trivial".

A: We understand that the order of events during the development of the methodology may be confusing. The fact is that we first discovered the qualitative presence of low-level mosaic epimutations in BRCA1 by methylation specific qPCR and used this method for our initial case-control assessment (doi: 10.7326/M17-0101). However, given the low accuracy of qPCR in very high Cp-numbers (in the range of 38-42), we saw it as imperative to develop a high-depth NGS-based assay capable of delivering high sensitivity, methylation calls on individual CpGs as well as allele-specific information. Given the breakthrough findings regarding the biological relevance of low-level mosaic epimutations, we saw it as critical to the field to provide a method by which such epimutations could be detected and quantified reliably.

The specific contributions of this manuscript are unclear. The manuscript claims to present a computational framework, but also cites published work from the same authors that appears to have already used the framework (Lønning et al. 2022). If this is the case, this disclosure should be made more transparent.

A: The present manuscript was deposited and appeared online prior to publication of the Lønning et al., 2022 paper which cites this manuscript. While they are linked, this manuscript describes the computational

method and its benefits as compared to other tools, and is aimed at bioinformaticians, while the subsequent Lønning et al., 2022 paper and another preprint (doi:10.1101/2023.05.14.23289949) describe experimental findings obtained by using this method and are aimed at clinical oncologists. In the revised manuscript, we have added this disclosure in the Declarations section. If this is a breach of journal style and the Editor would prefer us to move this statement somewhere else in the manuscript, we will of course do so.

Justification is needed for why "thresholding sequencing reads by their average methylation level will reduce the effect of biological and technical variance."

A: We realise that this was not properly explained in the original manuscript. To make the point clear in the revised manuscript, we have provided an extended explanation, replacing the previous sentence. It now reads as follows: “As number of events that lead to variance in methylation (base deamination, random single-base methylation events and sequencing errors) is limited at the level of individual reads (only a fraction of CpGs might be affected within the same read), the average methylation level of the read will be moderately affected by such events and can help distinguish hyper- from hypomethylated epialleles. We therefore hypothesized that thresholding sequence reads by their average methylation level will reduce the effect of biological and technical variance and facilitate the detection of infrequent hypermethylation events”. Notably, as we showed by sensitivity analyses, this hypothesis was correct.

Justification and motivation for the VEF metric is not provided, nor are all details provided on how it's calculated. I tried to follow the description for individual cytosines, but am immediately stuck on how to define Calpha for cytosine i. Calpha is described as the number of methylated cytosines in read pairs passing the threshold - it's not clear what is being counted over. Presumably this should only be considering reads that overlap that particular cytosine i, and perhaps it's counting over all cytosines in all such reads, but this is counting multiple times per cytosine (since more than 1 read can overlap a cytosine), so this seems to contradict the description. Moreover, if I just take the description at face value as "epialleles with similar methylation properties that is defined by thresholding", it's not clear what this represents biologically. It seems to intend to measure variability of methylation.

A: We understand that VEF is a newly introduced metric and that it should be explained in detail. Instead of providing more text, we have now included a panel B in Figure 1, where we graphically explain how VEF values are calculated at the level of individual bases. We believe that this figure panel, with its accompanying legend, will make the underlying calculations of VEF more easily accessible for the reader. In addition, the extensive online documentation to which we point our users (at https://bbcg.github.io/epialleleR/articles/epialleleR.html or https://bioconductor.org/packages/devel/bioc/vignettes/epialleleR/inst/doc/epialleleR.html) provides further clarifications and scenarios for specific analyses.

It's not clear how VEF metrics compare to established measures of methylation variability and/or allelic variation of methylation (e.g. Variably Methylated Region identification, methHaplo, methtuple, DAMEfinder). There are alternatives discussed in terms of entropy and discordant reads, but they are not compared in the sensitivity analysis study of WBC.

A: Variably methylated regions (VMR), by definition, are the regions showing variable methylation levels between samples. Such regions are not what epialleleR is designed to quantify. Unlike VMR tools, epialleleR outputs per-base value (VEF) that can significantly boost sensitivity of aberrant or differentially methylated region detection tools for the detection of mosaic epimutations (epimutations present in 1 out of >100 cells). This is presented in the sensitivity analyses that employ the best to our knowledge DMR analysis tool (DMRcate).
We agree with the Reviewer that it is always a good practice to compare to established tools. However, the tools suggested by Reviewer (methHaplo, methtuple, DAMEfinder) actually have different purposes than epialleleR. They are used to assemble methylation haplotypes (methHaplo), detect changes in allele-specific methylation between two groups of samples (DAMEfinder), or count m-tuples in reads (methtuple). While they use BAM files as an input, none of them is able to output a metric which is directly comparable to VEF (per-CpG methylation level) or its purpose (to count concordant hypermethylation events, i.e., epimutations). For example, methHaplo specifically excludes CpGs with low methylation level (beta<0.1)

as they are not informative for its purpose. In contrast, epialleleR is designed to detect and quantify rare epimutations, can extract all epialleles and can use optional VCF data to correlate SNVs with methylation – this was used by us to discover prenatal nature of BRCA1 epimutations that lead to cancer in adulthood. Besides, epialleleR can itself call methylation and use BAM files produced by virtually any aligners (which is not the case for tools mentioned above).
The other metrics (entropy, PDR, etc) are not per-base metrics and cannot be used for differential/aberrant methylation analysis.
As such, we feel it would leave the readers with a misleading impression about the tools and their purpose if we tried to present any comparison, and we have therefore not done this in the sensitivity analysis section.

A major limitation of the interpretation of so-called epialleles is the inability to distinguish allelic variation from cell type heterogeneity. This is acknowledged very briefly and very late in the manuscript. This needs to be stated up front. In addition, a discussion of potential remedies (if any) would be helpful.

A: We agree that cell type heterogeneity is a factor that must be taken into consideration in methylation analyses in general. But, while cell type heterogeneity can explain some of the methylation differences in other studies, the phenomenon we have described here does not depend on it. Only 1 CpG in this study and none of CpGs analysed in Lønning et al., 2022 have significantly different methylation levels between blood cell types. Regarding other regions, it is important to note that our method, epialleleR, is not itself biased by cell type heterogeneity because it is executed upstream of the data interpretation. epialleleR reports frequencies of individual epialleles or their groups (by methylation), while it is the downstream analysis (comparison of samples or their groups) which should account for any confounding factor, including, but not limited to, cell type heterogeneity. Given the possibility to perform allele-specific methylation calls with epialleleR, we actually believe it will be a useful addition to tools that may be used to address cell type heterogeneity, since methylation on different alleles in different tissues will indicate different methylation events to have occurred in different tissues of the same individual.

The simulated data are very unrealistic, which precludes interpretation of results. It seems that all CpGs were simulated with the same methylation level of 50%, and all other cyosines at 0.25% methylation levels. More importantly, introducing random sequencing errors is unrealistic, as per base sequence quality is known to be generally lower in read 2 compared to read 1. Further, there's a known increase in read 2 of M bias in bisulfite sequencing. With a more realistic error generating process, I expect choosing base with highest quality to be effectively the same as trimming read 2, meaning the results presented here are an artifact of simulation choices.

A: The purpose of the simulated data is to have a known level of methylation of all cytosines, so that the reported values can be compared to the expected ones. While the ground truth was chosen to be exactly 50% methylation in CG context and approximately 0.25% in CHG and CHH ones, this does not undermine the results of the comparison. We believe, the values applied are not relevant per se; they could have been 5% or 95% or anything else. The tools are still compared based on the lowest difference of observed (reported) values to the expected ones.
The Sherman bisulfite read simulator used by us here is a state-of-the-art tool, which uses the most realistic simulation strategy available. The errors are not entirely random but introduced according to exponential decay model (derived from real data). Simulated per-base quality scores follow the same curve as well. As pointed out by the Reviewer, it is indeed known that read 2 contains more base substitutions than read 1. However, the number of sequencing errors depends strongly on a sequence context and read length, therefore errors are present in both read 1 and read 2, and are more abundant at their ends. It is a quite common situation that a lower-quality 3' end of read 1 overlaps with higher-quality region of read 2 (either middle or its 5'). A simple trimming of a higher-quality part of read 2 will then result in lower-quality bases retained. This is why the base quality-driven merging will naturally result in fewer errors and we therefore consider it to be favourable.
The subtle increase in reporting accuracy is not very valuable on its own, but together with the vast speed improvement it allows us to recommend our method for virtually every analysis of methylation sequencing BAM files when methylation needs to be called at individual cytosine residues.

Methods are not described in enough detail to reproduce results. For example, the details on usage of

Bismark in the accuracy simulation is not included (only for speed comparison).

A: The methylation reporting for accuracy comparison was done exactly as described for the speed comparison for all tools. For clarity, this has now been specified in the “Cytosine reporting accuracy comparison” paragraph in Materials and Methods of the revised manuscript.

Owing to the major concerns listed thus far, I am not able to fully review the "Sensitivity analyses" section at this time.

A: We hope that the revisions in the manuscript text and the comments made here in our response letter, are clarifying and that all parts of the manuscript are now easily accessible for the reader.

Minor:

Organization of manuscript could use improvement (e.g. breaking up full page+ paragraphs, making sure headers accurately reflect section content).

A: We are uncertain of which specific paragraphs and/or subheadings the Reviewer would recommend to edit. Going through the manuscript, we have found that some of our previous subheadings were not fully precise covering the contents of the following paragraphs. Therefore, we have made some minor changes for better concordance. Thus, new subheadings in the revised manuscripts are “Reporting accuracy analyses” in the Results section and “Processing speed comparison” in the Material and Methods section. In addition, some very long paragraphs were broken into smaller paragraphs.

epialleleR is marked in Table 1 as not requiring a reference genome. But isn't it needed to obtain the bam file? If not, then how was the bam file generated for use with epiallelR? It's not a fair comparison to mark Bismark as needing a reference genome (to carry out the bam file generation step), as far as I'm aware this isn't used in the methylation extractor step in Bismark (the input is a BAM/SAM file).

A: Bismark methylation extractor indeed requires reference genome in addition to BAM input. For the methylation reporting, epialleleR does not require reference genome (similar to methylKit, but unlike methylation reporting by DRAGEN or Bismark).

In table 2, 'mean methylation level' is not generally considered an accuracy metric. Some metric of difference from ground truth should be shown.

A: We agree with the Reviewer that “mean methylation level” is indeed not an accuracy metric. Therefore, we provided “variance” as a measure of the differences between observed and expected values. Both these values allow the reader to see what tool reports closest to expected methylation values (0.5 and 0.0025 for cytosines in CG and CHG/CHH contexts, respectively). In order to clarify this issue, we have changed the title of the table, so that it now additionally includes these expected values.

When the authors state "Concordantly methylated alleles (genomic regions with spatially extended / haplotype-specific methylation)", I think they just mean allele-specific methylation. If not, please clarify. The other terms used (concordant methylation or spatially extended methylation) are nonstandard, and, puzzlingly, not mentioned in the citations provided for this sentence, where I searched for additional context.

A: By concordantly methylated alleles we mean reads with concordant methylation of cytosines (e.g., most of CpGs either methylated or unmethylated). We agree that this was unclear in our original manuscript and have now replaced the sentence with a simple explanation for “Concordantly methylated alleles” in the first sentence of the “Sensitivity analyses” paragraph of the Results section.

In Figure 2A what region is shown and how was this chosen? It appears the beta values are shown on a log scale, which is a nonstandard choice. M-values would be advisable to check as well.

A: Figure 2A shows all 138 CpGs included in the sensitivity analyses, with their genomic position as a

categorical variable along x-axis. We understand that this was unclear and have now included an additional explanation in the figure legend.
Data are presented using log scale to illustrate exponential (3-fold) differences between cytosine methylation levels (1e-4 to 1e-2) between all samples. This would not be possible using a linear scale and we have therefore chosen to keep the log scale in the figure.
As epialleleR cytosine reports are similar to Bismark cytosine reports in that epialleleR outputs both numbers of methylated ("Ca" with thresholding or "C" without thresholding) and unmethylated ("T") cytosines per genomic position, the package users can utilize those counts in any downstream analyses in the form of a beta value or M-value. Here, we chose to present beta/VEF values only, as sequencing data is compared with methylation array data, for which M-values are not available.

In Figure 2D/E, a measure of specificity should be reported as well.

A: The experimental setup allowed us to extract only the total number of real positive regions (P), the number of true positive regions (TP), and the number of false negative regions (FN=P–TP) for every analysis, while the numbers of true negative (TN) or false positive (FP) regions were undefined. Therefore, recall, or true positive rate (TPR=TP/P) was chosen as a sensitivity metric. This is described in Materials and Methods and the TPRs are now presented in the revised manuscript.

Should state much earlier in the manuscript which types of methylation sequencing data are suitable for this approach.

A: We agree that it would be beneficial to make an early statement about this and have now included this earlier in the text (Introduction and epialleleR implementation sections).
--
