# Supplementary material for: epialleleR: an R/Bioconductor package for sensitive allele-specific methylation analysis in NGS data
Source: Gigascience. 2023 Oct 31;12:giad087. doi: 10.1093/gigascience/giad087 (PMC10622323; doi:10.1093/gigascience/giad087)
Supplement: giad087_Response_to_Reviewer_Comments_Revision_1 [file giad087_response_to_reviewer_comments_revision_1.pdf]

Author's Response To Reviewer Comments

Reviewer reports:

Reviewer #2: Thank you to the authors for submitting a revised version of the manuscript and responding point by point to the previously raised concerns. Below you'll find a point by point response with my new comments labeled "Revision", author response labeled "A", and original comments labeled "Initial Submission". I have removed some comments that have been satisfactorily addressed (thank you!).

Answer to revision: we thank the Editor and the Reviewer for the opportunity to further improve our manuscript and hope that our answers and changes to the second revision have made this manuscript more clear and valuable for readers. In the following, the current answers start with the text “Answer to revision”.

***

Major:

Initial Submission: The manuscript lacks clarity in the presentation of the scientific question. The authors need to state their definition of mosaic methylation, and in addition how it relates to allele-specific methylation.

A: We agree that we could be more clear in these statements and in the revised version we consistently use the term "mosaic epimutations" instead of "mosaic methylation" in order to avoid confusion. In addition, we have provided simple statements defining the terminology in the text (e.g definition of "mosaic" in the introduction). In the Results section, we have also added a statement explaining the need for single-nucleotide variants to enable allele-specific methylation calls, clarifying how such allele-specific calls can be performed using epialleleR.

Revision: The term 'mosiac methylation' is still used in the revised version (see paragraph 2 of Introduction. Although many usages of the term have now been changed to 'mosaic epimutations', the term 'epimutation' is still not defined. It is not clear how an epimutation or 'epigenetic disturbances' are distinguished from background inter- and intra-individual variability. The terminology here implies something is abnormal, so the authors need to state how they decide when an epigenetic variant is different from 'normal' variation. With regard to the point about how the concept of epimutation is related to allele-specific methylation, the authors mention they have added a sentence to the results explaining that optional allele-specific methylation calls require SNP data. This seems to suggest that epimutations are distinct from allele-specific methylation, and this should be made clear when defining epimutations.

Answer to revision: We have now replaced the term in the Introduction, for consistency with the rest of the manuscript. In the last sentence of the Discussion, we still use the term “mosaic methylation events” since the meaning in that sentence is slightly different and does not necessarily fulfil the definition of “epimutations”.
In the revised manuscript we have updated the definition of term “epimutation” (early in the Introduction), including the description of an epimutation in a typical transcriptionally active gene as causing downregulation of expression, as well as giving examples on aberrant hypermethylation (epimutations) in BRCA1, MGMT and MLH1 tumour suppressors and their consequences. We also give some references to papers that explain the meaning of this term.
We have also included additional explanation on allele specificity of epimutations (in the epialleleR Implementation section).

***

Original submission: The motivation for development of new methodology as presented is somewhat contradictory. If the previously identified DNA methylation changes are so small (0.03-1%), and, as the

authors state, "indistinguishable from the background methylation level", then how were they detected in the first place? Further, the analysis task is described by the authors as "trivial".

A: We understand that the order of events during the development of the methodology may be confusing. The fact is that we first discovered the qualitative presence of lowlevel mosaic epimutations in BRCA1 by methylation specific qPCR and used this method for our initial case-control assessment (doi: 10.7326/M17-0101). However, given the low accuracy of qPCR in very high Cp-numbers (in the range of 38-42), we saw it as imperative to develop a high-depth NGS-based assay capable of delivering high sensitivity, methylation calls on individual CpGs as well as allele-specific information. Given the breakthrough findings regarding the biological relevance of lowlevel mosaic epimutations, we saw it as critical to the field to provide a method by which such epimutations could be detected and quantified reliably.

Revision: The contradictory nature of describing the methodology as 'trivial' and also 'nontrivial' within the same manuscript remains. This makes it difficult to assess the impact of the work. More importantly, it does not appear that the revisions include any mention of the limitations of the earlier qPCR approach providing motivation for the current methodology, as described in the authors' response. Additionally, the author's response that they "saw it as imperative to develop a high-depth NGS-based assay capable of delivering high sensitivity, methylation calls on individual CpGs as well as allele-specific information" is a bit puzzling given (1) that they have not developed a new assay, only a computational approach, and (2) the previously mentioned lack of clarity on the topic of allele-specific methylation.

Answer to revision: We understand that the descriptions of the methodology was not consistent and have now removed the word “trivial” from the text. We have also included a sentence about the limitations of qPCR in the Introduction. We have kept this as a part of the Introduction since the complete details of wet-lab methods are given in our previous publication (Lønning et al. 2022) and are not a subject of the present manuscript. We have added further explanations for allele-specific methylation (see previous comment/response) and hope that the additional explanations of epimutations are satisfactory.

***

Original submission: The specific contributions of this manuscript are unclear. The manuscript claims to present a computational framework, but also cites published work from the same authors that appears to have already used the framework (Lønning et al. 2022). If this is the case, this disclosure should be made more transparent.

A: The present manuscript was deposited and appeared online prior to publication of the Lønning et al., 2022 paper which cites this manuscript. While they are linked, this manuscript describes the computational method and its benefits as compared to other tools, and is aimed at bioinformaticians, while the subsequent Lønning et al., 2022 paper and another preprint (doi:10.1101/2023.05.14.23289949) describe experimental findings obtained by using this method and are aimed at clinical oncologists. In the revised manuscript, we have added this disclosure in the Declarations section. If this is a breach of journal style and the Editor would prefer us to move this statement somewhere else in the manuscript, we will of course do so.

Revision: Thank you for the additional information regarding prior publication of epialleleR. The Editor will be able to better speak to journal policy, but as a reader I would strongly prefer to also have this declaration included in the main text.

Answer to revision: We have now specified that the method has been used in previous work, in the third to last paragraph of the Introduction. Further, we have also updated the declaration at the end of the manuscript with one more reference that uses epialleleR.

***

Original submission: Justification is needed for why "thresholding sequencing reads by their average methylation level will reduce the effect of biological and technical variance."

A: We realise that this was not properly explained in the original manuscript. To make the point clear in the revised manuscript, we have provided an extended explanation, replacing the previous sentence. It now reads as follows: "As number of events that lead to variance in methylation (base deamination, random single-base methylation events and sequencing errors) is limited at the level of individual reads (only a fraction of CpGs might be affected within the same read), the average methylation level of the read will be moderately affected by such events and can help distinguish hyper- from hypomethylated epialleles. We therefore hypothesized that thresholding sequence reads by their average methylation level will reduce the effect of biological and technical variance and facilitate the detection of infrequent hypermethylation events". Notably, as we showed by sensitivity analyses, this hypothesis was correct.

Revision: The link between the newly added statement and the hypothesis is not entirely clear to me. If the average methylation level per read is moderately affected by events such as base deamination, random single-base methylation events, and sequencing errors, how can one distinguish those types of events from epimutations or 'epialleles' (this term also needs to be defined the first time it is used)?

Answer to revision: In a read covering, e.g., 10 CpGs within a fully methylated allele, the average methylation level would be 1.0 if all 10 CpGs are methylated. If one of the CpGs is affected by an artefact causing no methylation to be detected, the average level would be 0.9 and the read would still be counted as hypermethylated and representing an epimutation (given a threshold of, e.g., 0.5). In the opposite scenario, an unmethylated read with a false-positive CpG methylation will have an average methylation level of 0.1 and not deemed as hypermethylated. We believe the thresholding thus provides more robustness against random artefacts.
We have now included the explanation of the term “epiallele” together with a reference (Introduction section) and also included an additional explanatory sentence for the effect of the thresholding in the “epialleleR implementation” paragraph of the Results section.

***

Original submission: Justification and motivation for the VEF metric is not provided, nor are all details provided on how it's calculated. I tried to follow the description for individual cytosines, but am immediately stuck on how to define Calpha for cytosine i. Calpha is described as the number of methylated cytosines in read pairs passing the threshold - it's not clear what is being counted over. Presumably this should only be considering reads that overlap that particular cytosine i, and perhaps it's counting over all cytosines in all such reads, but this is counting multiple times per cytosine (since more than 1 read can overlap a cytosine), so this seems to contradict the description. Moreover, if I just take the description at face value as "epialleles with similar methylation properties that is defined by thresholding", it's not clear what this represents biologically. It seems to intend to measure variability of methylation.

A: We understand that VEF is a newly introduced metric and that it should be explained in detail. Instead of providing more text, we have now included a panel B in Figure 1, where we graphically explain how VEF values are calculated at the level of individual bases. We believe that this figure panel, with its accompanying legend, will make the underlying calculations of VEF more easily accessible for the reader. In addition, the extensive online documentation to which we point our users (at https://bbcg.github.io/epialleleR/articles/epialleleR.html or https://bioconductor.org/packages/devel/bioc/vignettes/epialleleR/inst/doc/epialleleR.ht ml) provides further clarifications and scenarios for specific analyses.

Revision: Thank you for providing the graphical description. It is now much clearer what the parameters in the VEF calculation are describing. However, related to previous concerns, there is no justification for why each of the example patterns presented in newly added Figure 1B can be attributed based on a single read to: sequencing artifacts, 'true biologically relevant epimutations', incomplete bisulfite conversion, or 'scattered methylation'.

Answer to revision: We have now tried to make this clearer for the reader in two ways: In addition to the new explanations on epimutations included in the Introduction, we have also inserted extra explanatory specifications to the different patterns in Figure 1B, in the legend to the figure. We hope this will help clarifying the point.

***

Original submission: It's not clear how VEF metrics compare to established measures of methylation variability and/or allelic variation of methylation (e.g. Variably Methylated Region identification, methHaplo, methtuple, DAMEfinder). There are alternatives discussed in terms of entropy and discordant reads, but they are not compared in the sensitivity analysis study of WBC.

A: Variably methylated regions (VMR), by definition, are the regions showing variable methylation levels between samples. Such regions are not what epialleleR is designed to quantify. Unlike VMR tools, epialleleR outputs per-base value (VEF) that can significantly boost sensitivity of aberrant or differentially methylated region detection tools for the detection of mosaic epimutations (epimutations present in 1 out of >100 cells). This is presented in the sensitivity analyses that employ the best to our knowledge DMR analysis tool (DMRcate). We agree with the Reviewer that it is always a good practice to compare to established tools. However, the tools suggested by Reviewer (methHaplo, methtuple, DAMEfinder) actually have different purposes than epialleleR. They are used to assemble methylation haplotypes (methHaplo), detect changes in allele-specific methylation between two groups of samples (DAMEfinder), or count m-tuples in reads (methtuple). While they use BAM files as an input, none of them is able to output a metric which is directly comparable to VEF (per-CpG methylation level) or its purpose (to count concordant hypermethylation events, i.e., epimutations). For example, methHaplo specifically excludes CpGs with low methylation level (beta < 0.1) as they are not informative for its purpose. In contrast, epialleleR is designed to detect and quantify rare epimutations, can extract all epialleles and can use optional VCF data to correlate SNVs with methylation - this was used by us to discover prenatal nature of BRCA1 epimutations that lead to cancer in adulthood. Besides, epialleleR can itself call methylation and use BAM files produced by virtually any aligners (which is not the case for tools mentioned above). The other metrics (entropy, PDR, etc) are not per-base metrics and cannot be used for differential/aberrant methylation analysis. As such, we feel it would leave the readers with a misleading impression about the tools and their
purpose if we tried to present any comparison, and we have therefore not done this in the sensitivity analysis section.

Revision: When the authors are addressing my first major concern, which is regarding the clarity of presentation of the purpose of their method, some of the discussion provided in the authors' response to this point may be useful to include in a revised version.

Answer to revision: In this manuscript, we have focused our attention on explaining what epialleleR is, and we are grateful for the Reviewer's comments as they help us to identify parts of manuscript that needed clarifications. Some of the points mentioned above were indeed included in the revised manuscript. At the same time, we would like to restrict the length of explanations on what epialleleR is not, as we believe we have compared epialleleR with the most relevant tools.

***

Original submission: A major limitation of the interpretation of so-called epialleles is the inability to distinguish allelic variation from cell type heterogeneity. This is acknowledged very briefly and very late in the manuscript. This needs to be stated up front. In addition, a discussion of potential remedies (if any) would be helpful.

A: We agree that cell type heterogeneity is a factor that must be taken into consideration in methylation analyses in general. But, while cell type heterogeneity can explain some of the methylation differences in other studies, the phenomenon we have described here does not depend on it. Only 1 CpG in this study and none of CpGs analysed in Lønning et al., 2022 have significantly different methylation levels between blood cell types. Regarding other regions, it is important to note that our method, epialleleR, is not itself biased by cell type heterogeneity because it is executed upstream of the data interpretation. epialleleR reports frequencies of individual epialleles or their groups (by methylation), while it is the downstream analysis (comparison of samples or their groups) which should account for any confounding factor, including, but not limited to, cell type heterogeneity. Given the possibility to perform allele-specific

methylation calls with epialleleR, we
actually believe it will be a useful addition to tools that may be used to address cell type heterogeneity, since methylation on different alleles in different tissues will indicate different methylation events to have occurred in different tissues of the same individual.

Revision: While the authors assert that the limited set of CpGs associated with one particular gene (BRCA1) do not widely exhibit differential methylation levels by cell type, they do not propose that their tool is aimed at analysing only 37 CpGs. Instead, they argue the tool is applicable on methylation sequencing data with base-level resolution - including whole genome bisulfite data. As such, my original concern remains regarding the confounding factor of cell type heterogeneity: "This is acknowledged very briefly and very late in the manuscript. This needs to be stated up front. In addition, a discussion of potential remedies (if any) would be helpful."

Answer to revision: We have now included a specification of this in the last paragraph of the Introduction section. However, we would like to point out that similar to Bismark and other tools that simply transform NGS reads into counts (e.g., of nucleotides or molecules), epialleleR is not confounded by cell-type heterogeneity per se. It is affected by cell-type heterogeneity to the same extent as next-generation sequencing itself, as a pre-analytical bias. In reality, it is the downstream differential gene expression or gene methylation analyses – statistical analyses performed to test hypotheses – that must include all potential confounding factors into the account (such as the sample quality and composition).
We do agree that cell-type heterogeneity is something that many investigators worry about when interpreting methylation data. In addition to the new sentence, we feel that we have acknowledged cell type heterogeneity by the previous statements.
As a note on biological interpretation of hypermethylation within tumour suppressor gene promoters (according to the data we have so far), both quantitative (allele frequency of 0.03% to 20%) and qualitative (appears on the same allele within single individual and on any allele in population) aspects of methylation within BRCA1 and MGMT argue against cell type heterogeneity to influence the findings. The same goes for the fact that the mosaic methylation of such genes are linked to cancer risk, while cell type composition in a blood sample is not.

***

Original submission: The simulated data are very unrealistic, which precludes interpretation of results. It seems that all CpGs were simulated with the same methylation level of 50%, and all other cyosines at 0.25% methylation levels. More importantly, introducing random sequencing errors is unrealistic, as per base sequence quality is known to be generally lower in read 2 compared to read 1. Further, there's a known increase in read 2 of M bias in bisulfite sequencing. With a more realistic error generating process, I expect choosing base with highest quality to be effectively the same as trimming read 2, meaning the results presented here are an artifact of simulation choices.

A: The purpose of the simulated data is to have a known level of methylation of all cytosines, so that the reported values can be compared to the expected ones. While the ground truth was chosen to be exactly 50% methylation in CG context and approximately 0.25% in CHG and CHH ones, this does not undermine the results of the comparison. We believe, the values applied are not relevant per se; they could have been 5% or 95% or anything else. The tools are still compared based on the lowest difference of observed (reported) values to the expected ones. The Sherman bisulfite read simulator used by us here is a state-of-the-art tool, which uses the most realistic simulation strategy available. The errors are not entirely random but introduced according to exponential decay model (derived from real data). Simulated per-base quality scores follow the same curve as well. As pointed out by the Reviewer, it is indeed known that read 2 contains more base substitutions than read 1.
However, the number of sequencing errors depends strongly on a sequence context and read length, therefore errors are present in both read 1 and read 2, and are more abundant at their ends. It is a quite common situation that a lower-quality 3' end of read 1 overlaps with higher-quality region of read 2 (either middle or its 5'). A simple trimming of a higher-quality part of read 2 will then result in lower-quality bases retained. This is why the base quality-driven merging will naturally result in fewer errors and we therefore consider it to be favourable. The subtle increase in reporting accuracy is not very valuable on its own, but together with the vast speed improvement it allows us to recommend our method for virtually every

analysis of methylation sequencing BAM files when methylation needs to be called at individual cytosine residues.

Revision: Thank you for the additional information regarding the exponential decay model of errors - this information would be helpful to add to the manuscript, as not all readers will be intimately familiar with the arguments of this particular simulator software.

Answer to revision: We agree with the Reviewer and have added an additional explanation on quality-based read merging and read simulation to the revised manuscript (including the mentioned decay model of errors) under “Cytosine reporting accuracy comparison” in the Materials and Methods section.

***

Original submission: Owing to the major concerns listed thus far, I am not able to fully review the "Sensitivity analyses" section at this time.

A: We hope that the revisions in the manuscript text and the comments made here in our response letter, are clarifying and that all parts of the manuscript are now easily accessible for the reader.

Revision: Many of the original concerns have not been fully addressed. However, at this time I'd like to point out that the sensitivity analysis section seems to be focused on analyses that only include a handful of specific regions of interest. Are these required for detection of 'epialleles'? If not, an example / evaluation that does not require a priori specification of these regions (e.g. is more of a genome-wide investigation) would be helpful, as well as a discussion of region-based vs individual cytosine-based analyses as they pertain to epialleleR.

Answer to revision: We hope the current revision addresses all Reviewer’s concerns. The applications of epialleleR are of course not limited to five tumour suppressor genes included in this manuscript. In fact, for the accuracy/speed analyses we used whole-genome and genome-wide data as well, and epialleleR is perfectly applicable (if not an optimal tool) for such data. Continuously updated online documentation of epialleleR includes other analyses one could perform, and in every given case they depend on a scientific question one may have. In other studies where we have used epialleleR, we performed ultradeep methylation sequencing of a single gene (BRCA1), while here we report five of them, not only presenting the tool itself but also suggesting that mosaic epimutations in tumour suppressors other then BRCA1 can be widespread in the population. We believe it is very likely that users will expand the application of epialleleR to different targeted panels of promoters of their interest. Expanding the epimutation analysis genome-wide will require ultradeep genome-wide methylation sequencing which we believe is very resource demanding and out of the scope of the present manuscript, but it is certainly something we may foresee for the future.

Minor:

***

Original submission: epialleleR is marked in Table 1 as not requiring a reference genome. But isn't it needed to obtain the bam file? If not, then how was the bam file generated for use with epiallelR? It's not a fair comparison to mark Bismark as needing a reference genome (to carry out the bam file generation step), as far as I'm aware this isn't used in the methylation extractor step in Bismark (the input is a BAM/SAM file).

A: Bismark methylation extractor indeed requires reference genome in addition to BAM input. For the methylation reporting, epialleleR does not require reference genome (similar to methylKit, but unlike methylation reporting by DRAGEN or Bismark).

Revision: Can you please point me to a reference that shows you need a reference genome to run

Bismark's methylation extractor? The documentation for this tool does not mention anything about a reference genome: https://felixkrueger.github.io/Bismark/options/methylation_extraction/. As I pointed out before, like epialleleR, it only requires BAM/SAM from an alignment.

Answer to revision: It is the “--genome_folder” option which is mandatory to make cytosine reports. It does not seem to be required for bed graph or M-bias reports; we have now added this note to the manuscript (Table 1).

***

Original submission: When the authors state "Concordantly methylated alleles (genomic regions with spatially extended / haplotype-specific methylation)", I think they just mean allele-specific methylation. If not, please clarify. The other terms used (concordant methylation or spatially extended methylation) are nonstandard, and, puzzlingly, not mentioned in the citations provided for this sentence, where I searched for additional context.

A: By concordantly methylated alleles we mean reads with concordant methylation of cytosines (e.g., most of CpGs either methylated or unmethylated). We agree that this was unclear in our original manuscript and have now replaced the sentence with a simple explanation for "Concordantly methylated alleles" in the first sentence of the "Sensitivity analyses" paragraph of the Results section.

Revision: Thank you for the clarification. The revised statement now refers only to alleles with most CpGs methylated and is rather vague. It's not clear why reads with most CpGs unmethylated are not biologically important. What do the authors mean by "biological importance" here? Note that lots of reads will show most CpGs either methylated or unmethylated, so the specific references cited don't seem to support this broad claim.

Answer to revision: We have updated this sentence to cover both hypo- and hypermethylated alleles. The references confirm the current statement by giving examples on cancer-related and treatment resistance-related allelic events. We would like to add that although the current knowledge is rather limited, fully methylated BRCA1 alleles are not normal: only 5-8% of women have them at low-mosaic levels, and their presence significantly increases risk of future cancer diagnosis. The same might be the case for many other tumour suppressor genes.

***

Original submission: In Figure 2A what region is shown and how was this chosen? It appears the beta values are shown on a log scale, which is a nonstandard choice. M-values would be advisable to check as well.

A: Figure 2A shows all 138 CpGs included in the sensitivity analyses, with their genomic position as a categorical variable along x-axis. We understand that this was unclear and have now included an additional explanation in the figure legend. Data are presented using log scale to illustrate exponential (3-fold) differences between cytosine methylation levels (1e-4 to 1e-2) between all samples. This would not be possible using a linear scale and we have therefore chosen to keep the log scale in the figure. As epialleleR cytosine reports are similar to Bismark cytosine reports in that epialleleR outputs both numbers of methylated ("Ca" with thresholding or "C" without thresholding) and unmethylated ("T") cytosines per genomic position, the package users can utilize those counts in any downstream analyses in the form of a beta value or M-value. Here, we chose to present beta/VEF values only, as sequencing data is compared with methylation array data, for which M-values are not available.

Revision: M-values are certainly applicable to array data (https://doi.org/10.1186/1471-2105-11-587). However, after reviewing a bit about the experimental setup (that this is focused on primarily unmethylated regions), the motivation for using M-values which allow for comparing regions of varying baseline methylation level is not very relevant.

Answer to revision: We thank Reviewer for the comment.

***

Original submission: In Figure 2D/E, a measure of specificity should be reported as well.

A: The experimental setup allowed us to extract only the total number of real positive regions (P), the number of true positive regions (TP), and the number of false negative regions (FN=P-TP) for every analysis, while the numbers of true negative (TN) or false positive (FP) regions were undefined. Therefore, recall, or true positive rate (TPR=TP/P) was chosen as a sensitivity metric. This is described in Materials and Methods and the TPRs are now presented in the revised manuscript.

Revision: Can the authors please explain what about the experimental setup precludes identification of TN and FP?

Answer to revision: For admixing we used FASTQ files derived from amplicon sequencing of fully methylated DNA sample and 10 male samples that showed no presence of epimutations in any of the five assessed regions. When methylated reads were admixed, all five regions became positive (P), and no regions were negative (N). Therefore, we only knew TP and FN=P-TP, but not TN or FP

***
