# Supplementary material for: epialleleR: an R/Bioconductor package for sensitive allele-specific methylation analysis in NGS data
Source: Gigascience. 2023 Oct 31;12:giad087. doi: 10.1093/gigascience/giad087 (PMC10622323; doi:10.1093/gigascience/giad087)
Supplement: giad087_Response_to_Reviewer_Comments_Revision_2 [file giad087_response_to_reviewer_comments_revision_2.pdf]

Author's Response To Reviewer Comments

Reviewer reports:
Reviewer #2: Thank you to the authors for submitting a second revised version of the manuscript and responding point by point to the previously raised concerns. Below you'll find a point by point response with my new comments labeled "Revision 2", author responses labeled "A1" and "A2", and original comments labeled "Initial Submission" and "Revision", respectively. I have removed some comments that have been satisfactorily addressed (thank you!).

We thank the Reviewer for helping us to further improve this manuscript. Our answers to the latest comments are marked with “A3” and all the recent changes are highlighted in the manuscript.

Major:

Initial Submission: The manuscript lacks clarity in the presentation of the scientific question. The authors need to state their definition of mosaic methylation, and in addition how it relates to allele-specific methylation.

A1: We agree that we could be more clear in these statements and in the revised version we consistently use the term "mosaic epimutations" instead of "mosaic methylation" in order to avoid confusion. In addition, we have provided simple statements defining the terminology in the text (e.g definition of "mosaic" in the introduction). In the Results section, we have also added a statement explaining the need for single-nucleotide variants to enable allele-specific methylation calls, clarifying how such allele-specific calls can be performed using epialleleR.

Revision: The term 'mosiac methylation' is still used in the revised version (see paragraph 2 of Introduction. Although many usages of the term have now been changed to 'mosaic epimutations', the term 'epimutation' is still not defined. It is not clear how an epimutation or 'epigenetic disturbances' are distinguished from background inter- and intra-individual variability. The terminology here implies something is abnormal, so the authors need to state how they decide when an epigenetic variant is different from 'normal' variation. With regard to the point about how the concept of epimutation is related to allele-specific methylation, the authors mention they have added a sentence to the results explaining that optional allele-specific methylation calls require SNP data. This seems to suggest that epimutations are distinct from allele-specific methylation, and this should be made clear when defining epimutations.

A2: We have now replaced the term in the Introduction, for consistency with the rest of the manuscript. In the last sentence of the Discussion, we still use the term "mosaic methylation events" since the meaning in that sentence is slightly different and does not necessarily fulfil the definition of "epimutations".
In the revised manuscript we have updated the definition of term "epimutation" (early in the Introduction), including the description of an epimutation in a typical transcriptionally active gene as causing downregulation of expression, as well as giving examples on aberrant hypermethylation (epimutations) in BRCA1, MGMT and MLH1 tumour suppressors and their consequences. We also give some references to papers that explain the meaning of this term.
We have also included additional explanation on allele specificity of epimutations (in the epialleleR Implementation section).

Revision 2: Thank you for cleaning up the terminology and adding to the definition of epimutation. However, as raised previously, I do not see where the authors have stated how they decide when an epigenetic variant arises to the level of an epimutation as now defined, as opposed to background inter- and intra- individual variability. In light of the newly added definitions and other revisions, I'll ask whether it is the case that epialleleR does not explicitly detect epimutations, but rather, provides an alternate

metric (the VEF) that can be used by other tools (e.g. ramr) to call them. Is this accurate? If so, this took quite some effort to sort out, so needs to be clarified (or if it's not true, then please do add an explanation of how epimutations are called).

A3: The reviewer is correct stating that epialleleR does not explicitly detect epimutations. It aids in detection by providing a quantitative metric (VEF) which allows to detect epimutations with high specificity and sensitivity, as we showed in Sensitivity analyses section (Fig. 2). It is possible to use the quantitative output of epialleleR (VEF values) as an input for differential/aberrant methylation analysis tools. It is also possible to apply a simple threshold and qualitatively split samples in two groups as containing or not containing epimutations, or to use VEF values as a quantitative measure of a frequency of epimutated alleles in samples. The latter (analysis without AMR/DMR tools) was used by us in two studies that cite epialleleR (references #12 and 23). We have now very briefly updated a note on VEF thresholding in the Discussion with references to these studies.
The discussion around "epigenetic variant" and "epimutation" is interesting and in many ways parallels the discussion around "genetic variant" vs. "mutation". We believe the decision on what changes constitute an epimutation should be taken by the user, based on the region of interest and knowledge about the normal methylation state within that region as well as potential pathogenicity of the change. Here, we describe, and used as an example, epimutations in promoters of tumour suppressor genes. In normal tissue, such regions are characterised by hypomethylation. We therefore think that aberrant hypermethylation, that results in gene silencing and increased cancer risk, within such regions would fulfil most researchers' understanding of the epimutation term. For general use, the thresholds for what constitutes a epimutation can vary and we have now inserted a paragraph in the Discussion section explaining that this is adjustable.

***

Original submission: Justification and motivation for the VEF metric is not provided, nor are all details provided on how it's calculated. I tried to follow the description for individual cytosines, but am immediately stuck on how to define Calpha for cytosine i. Calpha is described as the number of methylated cytosines in read pairs passing the threshold - it's not clear what is being counted over. Presumably this should only be considering reads that overlap that particular cytosine i, and perhaps it's counting over all cytosines in all such reads, but this is counting multiple times per cytosine (since more than 1 read can overlap a cytosine), so this seems to contradict the description. Moreover, if I just take the description at face value as "epialleles with similar methylation properties that is defined by thresholding", it's not clear what this represents biologically. It seems to intend to measure variability of methylation.

A: We understand that VEF is a newly introduced metric and that it should be explained in detail. Instead of providing more text, we have now included a panel B in Figure 1, where we graphically explain how VEF values are calculated at the level of individual bases. We believe that this figure panel, with its accompanying legend, will make the underlying calculations of VEF more easily accessible for the reader. In addition, the extensive online documentation to which we point our users (at https://bbcg.github.io/epialleleR/articles/epialleleR.html or https://bioconductor.org/packages/devel/bioc/vignettes/epialleleR/inst/doc/epialleleR.ht ml) provides further clarifications and scenarios for specific analyses.

Revision: Thank you for providing the graphical description. It is now much clearer what the parameters in the VEF calculation are describing. However, related to previous concerns, there is no justification for why each of the example patterns presented in newly added Figure 1B can be attributed based on a single read to: sequencing artifacts, 'true biologically relevant epimutations', incomplete bisulfite conversion, or 'scattered methylation'.

A2: We have now tried to make this clearer for the reader in two ways: In addition to the new explanations on epimutations included in the Introduction, we have also inserted extra explanatory specifications to the different patterns in Figure 1B, in the legend to the figure. We hope this will help clarifying the point.

Revision 2: Thank you to the authors for providing a qualitative description of each of the patterns. Related to the previous outstanding major concern about distinguishing epimutations from background variability, these definitions and cartoon example illustrations do not demonstrate that the chosen thresholds are

generally/widely able to accurately distinguish the 'true biologically relevant' variation from artifacts. In this figure, it should be acknowledged that these are example patterns the thresholds are chosen to distinguish between, but there is no guarantee that the metric will separate true biological variation from artifacts. Related, the thresholds seem to be something that can vary by region (as hinted on page 8 of the manuscript) but no guidance is provided for how to select it beyond the inclusion of default thresholds.

A3: Indeed, thresholds depend on the nature of regions of interest. In response to this and the point above, we have now added a paragraph on default parameters, and the possibility to adjust these, to the Discussion. In addition, we have inserted the requested clarification to the legend of Fig.1B. We hope that these additions will help readers in their analyses.

***

Original submission: A major limitation of the interpretation of so-called epialleles is the inability to distinguish allelic variation from cell type heterogeneity. This is acknowledged very briefly and very late in the manuscript. This needs to be stated up front. In addition, a discussion of potential remedies (if any) would be helpful.

A: We agree that cell type heterogeneity is a factor that must be taken into consideration in methylation analyses in general. But, while cell type heterogeneity can explain some of the methylation differences in other studies, the phenomenon we have described here does not depend on it. Only 1 CpG in this study and none of CpGs analysed in Lønning et al., 2022 have significantly different methylation levels between blood cell types. Regarding other regions, it is important to note that our method, epialleleR, is not itself biased by cell type heterogeneity because it is executed upstream of the data interpretation. epialleleR reports frequencies of individual epialleles or their groups (by methylation), while it is the downstream analysis (comparison of samples or their groups) which should account for any confounding factor, including, but not limited to, cell type heterogeneity. Given the possibility to perform allele-specific methylation calls with epialleleR, weactually
believe it will be a useful addition to tools that may be used to address cell type heterogeneity, since methylation on different alleles in different tissues will indicate different methylation events to have occurred in different tissues of the same individual.

Revision: While the authors assert that the limited set of CpGs associated with one particular gene (BRCA1) do not widely exhibit differential methylation levels by cell type, they do not propose that their tool is aimed at analysing only 37 CpGs. Instead, they argue the tool is applicable on methylation sequencing data with base-level resolution - including whole genome bisulfite data. As such, my original concern remains regarding the confounding factor of cell type heterogeneity: "This is acknowledged very briefly and very late in the manuscript. This needs to be stated up front. In addition, a discussion of potential remedies (if any) would be helpful."

A2: We have now included a specification of this in the last paragraph of the Introduction section. However, we would like to point out that similar to Bismark and other tools that simply transform NGS reads into counts (e.g., of nucleotides or molecules), epialleleR is not confounded by cell-type heterogeneity per se. It is affected by cell-type heterogeneity to the same extent as next-generation sequencing itself, as a pre-analytical bias. In reality, it is the downstream differential gene expression or gene methylation analyses - statistical analyses performed to test hypotheses - that must include all potential confounding factors into the account (such as the sample quality and composition).
We do agree that cell-type heterogeneity is something that many investigators worry about when interpreting methylation data. In addition to the new sentence, we feel that we have acknowledged cell type heterogeneity by the previous statements.

As a note on biological interpretation of hypermethylation within tumour suppressor gene promoters (according to the data we have so far), both quantitative (allele frequency of 0.03% to 20%) and qualitative (appears on the same allele within single individual and on any allele in population) aspects of methylation within BRCA1 and MGMT argue against cell type heterogeneity to influence the findings. The same goes for the fact that the mosaic methylation of such genes are linked to cancer risk, while cell type composition in a blood sample is not.

Revision 2: Thank you for including the statement regarding cell-type heterogeneity up front. By claiming the method detects epiALLELES or hypermethylated ALLELES, the wording 'allele' here directly implies something about the variation -- namely that it is within cell. Cell type heterogeneity directly confounds that. This is why I see this approach as fundamentally different from Bismark, and why I requested the added statement in the introduction.

A3: We thank the Reviewer for the comment and leave the wording from our last revision in the Introduction.

Minor:

***

Original submission: epialleleR is marked in Table 1 as not requiring a reference genome. But isn't it needed to obtain the bam file? If not, then how was the bam file generated for use with epiallelR? It's not a fair comparison to mark Bismark as needing a reference genome (to carry out the bam file generation step), as far as I'm aware this isn't used in the methylation extractor step in Bismark (the input is a BAM/SAM file).

A: Bismark methylation extractor indeed requires reference genome in addition to BAM input. For the methylation reporting, epialleleR does not require reference genome (similar to methylKit, but unlike methylation reporting by DRAGEN or Bismark).

Revision: Can you please point me to a reference that shows you need a reference genome to run Bismark's methylation extractor? The documentation for this tool does not mention anything about a reference genome: https://felixkrueger.github.io/Bismark/options/methylation_extraction/. As I pointed out before, like epialleleR, it only requires BAM/SAM from an alignment.

A2: It is the "--genome_folder" option which is mandatory to make cytosine reports. It does not seem to be required for bed graph or M-bias reports; we have now added this note to the manuscript (Table 1).

Revision 2: The option you mention does indeed require a reference genome, but as pointed out in Bismark documentation (https://felixkrueger.github.io/Bismark/bismark/methylation_extraction/#optional-genome-wide-cytosine-report-output), this is because it adds information in addition to the standard cytosine report, which is not part of a routine analysis: "The main difference to the bedGraph or coverage output is that every cytosine on both the top and bottom strands will be considered irrespective of whether they were actually covered by any reads in the experiment or not. For this to work one has to also specify the genome that was used for the Bismark alignments using the option --genome_folder . As for the bedGraph mode, this will only consider cytosines in CpG context by default but can be extended to cytosines in any sequence context by using the option --CX (cf. Appendix (III)). Be aware though that this might mean an output with individual lines for more than 1.1 billion cytosines for any large mammalian genome..." This is also not comparable to what epialleleR, so I'll reiterate that claiming that Bismark requires a reference genome to make a cytosine report like that given by epialleleR is not a fair comparison.

A3: We believe that the requirement of a reference genome is not something that makes a tool superior or inferior of other tools, but rather a different approach to the same task. Tools that require a reference genome will not report an incorrect context of a cytosine due to a sequencing error, although they may also not report valid bases that differ in their context from the reference genome (because a sample's genome is never the same as the reference genome). Examples of such are DRAGEN and whole-genome cytosine report of Bismark (see "invalid context" column in the Supplementary Table 1).
On the other hand, tools that do not require reference genome will not discard methylation information where the sample's genome is different from the reference genome, although they may report wrong context of a base when, e.g., one of the sequencing reads contained an error. Example of such: methylKit and bedGraph reports of Bismark.
In this manuscript (under "Reporting accuracy analyses") we compared tools using the settings that will lead to their most correct output in testing conditions. That is why we used cytosine reports from Bismark

and not the bedGraph reports. As stated in Table 1, Bismark may not require reference genome, and it is not something that makes it superior or inferior.
Of note, epialleleR is still different from the other tools. It does not require reference genome, however, it does not make mistakes in determining the context, because it collects information on all sequence contexts for each base (whether it is a C or not).
We have now modified Table 1, to clarify this for Bismark. In addition, we have added a sentence in Materials and Methods (under “Processing speed comparison”) to explain the same point and a paragraph in the “Reporting accuracy analyses” paragraph in the Results section that contain this information.
