# Supplementary material for: epialleleR: an R/Bioconductor package for sensitive allele-specific methylation analysis in NGS data
Source: Gigascience. 2023 Oct 31;12:giad087. doi: 10.1093/gigascience/giad087 (PMC10622323; doi:10.1093/gigascience/giad087)
Supplement: giad087_Response_to_Reviewer_Comments_Revision_3 [file giad087_response_to_reviewer_comments_revision_3.pdf]

Author's Response To Reviewer Comments

Dear Editor,
all the requested changes were introduced. We have attached versions with and without tracked changes.

Best regards,
Oleksii Nikolaienko
