# Supplementary material for: epialleleR: an R/Bioconductor package for sensitive allele-specific methylation analysis in NGS data
Source: Gigascience. 2023 Oct 31;12:giad087. doi: 10.1093/gigascience/giad087 (PMC10622323; doi:10.1093/gigascience/giad087)
Supplement: giad087_Reviewer_2_Report_Original_Submission — Keegan Korthauer -- 7/21/2023 Reviewed [file giad087_reviewer_2_report_original_submission.pdf]

Reviewer Report

Title: epialleleR: an R/Bioconductor package for sensitive allele-specific methylation analysis in NGS data

Version: Original Submission **Date:** 7/21/2023

Reviewer name: Keegan Korthauer

Reviewer Comments to Author:

Review of GIGA-D-23-00149
The manuscript presents a computational approach for detecting what the authors term "mosaic epimutations". However, I found it very difficult to review the merits of the contribution owing to some fundamental issues with presentation, missing key details that motivate/justify the work, and problematic choices for evaluation/comparison. Below I list detailed concerns, separated by major and minor.
Major:
The manuscript lacks clarity in the presentation of the scientific question. The authors need to state their definition of mosaic methylation, and in addition how it relates to allele-specific methylation.
The motivation for development of new methodology as presented is somewhat contradictory. If the previously identified DNA methylation changes are so small (0.03-1%), and, as the authors state, "indistinguishable from the background methylation level", then how were they detected in the first place? Further, the analysis task is described by the authors as "trivial".
The specific contributions of this manuscript are unclear. The manuscript claims to present a computational framework, but also cites published work from the same authors that appears to have already used the framework (LÃ¸nning et al. 2022). If this is the case, this disclosure should be made more transparent.
Justification is needed for why "thresholding sequencing reads by their average methylation level will reduce the effect of biological and technical variance."
Justification and motivation for the VEF metric is not provided, nor are all details provided on how it's calculated. I tried to follow the description for individual cytosines, but am immediately stuck on how to define Calpha for cytosine i. Calpha is described as the number of methylated cytosines in read pairs passing the threshold - it's not clear what is being counted over. Presumably this should only be considering reads that overlap that particular cytosine i, and perhaps it's counting over all cytosines in all such reads, but this is counting multiple times per cytosine (since more than 1 read can overlap a cytosine), so this seems to contradict the description. Moreover, if I just take the description at face value as "epialleles with similar methylation properties that is defined by thresholding", it's not clear what this represents biologically. It seems to intend to measure variability of methylation.
It's not clear how VEF metrics compare to established measures of methylation variability and/or allelic variation of methylation (e.g. Variably Methylated Region identification, methHaplo, methtuple, DAMEfinder). There are alternatives discussed in terms of entropy and discordant reads, but they are not compared in the sensitivity analysis study of WBC.

A major limitation of the interpretation of so-called epialleles is the inability to distinguish allelic variation from cell type heterogeneity. This is acknowledged very briefly and very late in the manuscript. This needs to be stated up front. In addition, a discussion of potential remedies (if any) would be helpful.
The simulated data are very unrealistic, which precludes interpretation of results. It seems that all CpGs were simulated with the same methylation level of 50%, and all other cyosines at 0.25% methylation levels. More importantly, introducing random sequencing errors is unrealistic, as per base sequence quality is known to be generally lower in read 2 compared to read 1. Further, there's a known increase in read 2 of M bias in bisulfite sequencing. With a more realistic error generating process, I expect choosing base with highest quality to be effectively the same as trimming read 2, meaning the results presented here are an artifact of simulation choices.
Methods are not described in enough detail to reproduce results. For example, the details on usage of Bismark in the accuracy simulation is not included (only for speed comparison).
Owing to the major concerns listed thus far, I am not able to fully review the "Sensitivity analyses" section at this time.
Minor:
Organization of manuscript could use improvement (e.g. breaking up full page+ paragraphs, making sure headers accurately reflect section content).
epialleleR is marked in Table 1 as not requiring a reference genome. But isn't it needed to obtain the bam file? If not, then how was the bam file generated for use with epiallelR? It's not a fair comparison to mark Bismark as needing a reference genome (to carry out the bam file generation step), as far as I'm aware this isn't used in the methylation extractor step in Bismark (the input is a BAM/SAM file).
In table 2, 'mean methylation level' is not generally considered an accuracy metric. Some metric of difference from ground truth should be shown.
When the authors state "Concordantly methylated alleles (genomic regions with spatially extended / haplotype-specific methylation)", I think they just mean allele-specific methylation. If not, please clarify. The other terms used (concordant methylation or spatially extended methylation) are nonstandard, and, puzzlingly, not mentioned in the citations provided for this sentence, where I searched for additional context.
In Figure 2A what region is shown and how was this chosen? It appears the beta values are shown on a log scale, which is a nonstandard choice. M-values would be advisable to check as well.
In Figure 2D/E, a measure of specificity should be reported as well.
Should state much earlier in the manuscript which types of methylation sequencing data are suitable for this approach.

Methods

Are the methods appropriate to the aims of the study, are they well described, and are necessary controls included? Choose an item.

Conclusions

Are the conclusions adequately supported by the data shown? Choose an item.

Reporting Standards

Does the manuscript adhere to the journal's guidelines on minimum standards of reporting? Choose an item.

Choose an item.

Statistics

Are you able to assess all statistics in the manuscript, including the appropriateness of statistical tests used? Choose an item.

Quality of Written English

Please indicate the quality of language in the manuscript: Choose an item.

Declaration of Competing Interests

Please complete a declaration of competing interests, considering the following questions:

- Have you in the past five years received reimbursements, fees, funding, or salary from an organisation that may in any way gain or lose financially from the publication of this manuscript, either now or in the future?
- Do you hold any stocks or shares in an organisation that may in any way gain or lose financially from the publication of this manuscript, either now or in the future?
- Do you hold or are you currently applying for any patents relating to the content of the manuscript?
- Have you received reimbursements, fees, funding, or salary from an organization that holds or has applied for patents relating to the content of the manuscript?
- Do you have any other financial competing interests?
- Do you have any non-financial competing interests in relation to this paper?

If you can answer no to all of the above, write 'I declare that I have no competing interests' below. If your reply is yes to any, please give details below.

I declare that I have no competing interests

I agree to the open peer review policy of the journal. I understand that my name will be included on my report to the authors and, if the manuscript is accepted for publication, my named report including any attachments I upload will be posted on the website along with the authors' responses. I agree for my report to be made available under an Open Access Creative Commons CC-BY license (http://creativecommons.org/licenses/by/4.0/). I understand that any comments which I do not wish to be included in my named report can be included as confidential comments to the editors, which will not be published.

Choose an item.

To further support our reviewers, we have joined with Publons, where you can gain additional credit to further highlight your hard work (see: https://publons.com/journal/530/gigascience). On publication of this paper, your review will be automatically added to Publons, you can then choose whether or not to claim your Publons credit. I understand this statement.

Yes Choose an item.
