# Supplementary material for: epialleleR: an R/Bioconductor package for sensitive allele-specific methylation analysis in NGS data
Source: Gigascience. 2023 Oct 31;12:giad087. doi: 10.1093/gigascience/giad087 (PMC10622323; doi:10.1093/gigascience/giad087)
Supplement: giad087_Reviewer_2_Report_Revision_1 — Keegan Korthauer -- 8/14/2023 Reviewed [file giad087_reviewer_2_report_revision_1.pdf]

Reviewer Report

Title: epialleleR: an R/Bioconductor package for sensitive allele-specific methylation analysis in NGS data

Version: Revision 1 **Date:** 8/14/2023

Reviewer name: Keegan Korthauer

Reviewer Comments to Author:

Thank you to the authors for submitting a revised version of the manuscript and responding point by point to the previously raised concerns. Below you'll find a point by point response with my new comments labeled "Revision", author response labeled "A", and original comments labeled "Initial Submission". I have removed some comments that have been satisfactorily addressed (thank you!).
Major:
Initial Submission: The manuscript lacks clarity in the presentation of the scientific question. The authors need to state their definition of mosaic methylation, and in addition how it relates to allele-specific methylation.
A: We agree that we could be more clear in these statements and in the revised version we consistently use the term "mosaic epimutations" instead of "mosaic methylation" in order to avoid confusion. In addition, we have provided simple statements defining the terminology in the text (e.g definition of "mosaic" in the introduction). In the Results section, we have also added a statement explaining the need for single-nucleotide variants to enable allele-specific methylation calls, clarifying how such allele-specific calls can be performed using epialleleR.
Revision: The term 'mosiac methylation' is still used in the revised version (see paragraph 2 of Introduction. Although many usages of the term have now been changed to 'mosaic epimutations', the term 'epimutation' is still not defined. It is not clear how an epimutation or 'epigenetic disturbances' are distinguished from background inter- and intra-individual variability. The terminology here implies something is abnormal, so the authors need to state how they decide when an epigenetic variant is different from 'normal' variation. With regard to the point about how the concept of epimutation is related to allele-specific methylation, the authors mention they have added a sentence to the results explaining that optional allele-specific methylation calls require SNP data. This seems to suggest that epimutations are distinct from allele-specific methylation, and this should be made clear when defining epimutations.
Original submission: The motivation for development of new methodology as presented is somewhat contradictory. If the previously identified DNA methylation changes are so small (0.03-1%), and, as the authors state, "indistinguishable from the background methylation level", then how were they detected in the first place? Further, the analysis task is described by the authors as "trivial".
A: We understand that the order of events during the development of the methodology may be confusing. The fact is that we first discovered the qualitative presence of lowlevel mosaic epimutations in BRCA1 by methylation specific qPCR and used this method for our initial case-control assessment (doi: 10.7326/M17-0101). However, given the low accuracy of qPCR in very high Cp-numbers (in the range of

38-42), we saw it as imperative to develop a high-depth NGS-based assay capable of delivering high sensitivity, methylation calls on individual CpGs as well as allele-specific information. Given the breakthrough findings regarding the biological relevance of lowlevel mosaic epimutations, we saw it as critical to the field to provide a method by which such epimutations could be detected and quantified reliably.
Revision: The contradictory nature of describing the methodology as 'trivial' and also 'nontrivial' within the same manuscript remains. This makes it difficult to assess the impact of the work. More importantly, it does not appear that the revisions include any mention of the limitations of the earlier qPCR approach providing motivation for the current methodology, as described in the authors' response. Additionally, the author's response that they "saw it as imperative to develop a high-depth NGS-based assay capable of delivering high sensitivity, methylation calls on individual CpGs as well as allele-specific information" is a bit puzzling given (1) that they have not developed a new assay, only a computational approach, and (2) the previously mentioned lack of clarity on the topic of allele-specific methylation.
Original submission: The specific contributions of this manuscript are unclear. The manuscript claims to present a computational framework, but also cites published work from the same authors that appears to have already used the framework (LÃ¸nning et al. 2022). If this is the case, this disclosure should be made more transparent.
A: The present manuscript was deposited and appeared online prior to publication of the LÃ¸nning et al., 2022 paper which cites this manuscript. While they are linked, this manuscript describes the computational method and its benefits as compared to other tools, and is aimed at bioinformaticians, while the subsequent LÃ¸nning et al., 2022 paper and another preprint (doi:10.1101/2023.05.14.23289949) describe experimental findings obtained by using this method and are aimed at clinical oncologists. In the revised manuscript, we have added this disclosure in the Declarations section. If this is a breach of journal style and the Editor would prefer us to move this statement somewhere else in the manuscript, we will of course do so.
Revision: Thank you for the additional information regarding prior publication of epialleleR. The Editor will be able to better speak to journal policy, but as a reader I would strongly prefer to also have this declaration included in the main text.
Original submission: Justification is needed for why "thresholding sequencing reads by their average methylation level will reduce the effect of biological and technical variance."
A: We realise that this was not properly explained in the original manuscript. To make the point clear in the revised manuscript, we have provided an extended explanation, replacing the previous sentence. It now reads as follows: "As number of events that lead to variance in methylation (base deamination, random single-base methylation events and sequencing errors) is limited at the level of individual reads (only a fraction of CpGs might be affected within the same read), the average methylation level of the read will be moderately affected by such events and can help distinguish hyper- from hypomethylated epialleles. We therefore hypothesized that thresholding sequence reads by their average methylation level will reduce the effect of biological and technical variance and facilitate the detection of infrequent hypermethylation events". Notably, as we showed by sensitivity analyses, this hypothesis was correct.
Revision: The link between the newly added statement and the hypothesis is not entirely clear to me. If the average methylation level per read is moderately affected by events such as base deamination, random single-base methylation events, and sequencing errors, how can one distinguish those types of

events from epimutations or 'epialleles' (this term also needs to be defined the first time it is used)?
Original submission: Justification and motivation for the VEF metric is not provided, nor are all details provided on how it's calculated. I tried to follow the description for individual cytosines, but am immediately stuck on how to define Calpha for cytosine i. Calpha is described as the number of methylated cytosines in read pairs passing the threshold - it's not clear what is being counted over. Presumably this should only be considering reads that overlap that particular cytosine i, and perhaps it's counting over all cytosines in all such reads, but this is counting multiple times per cytosine (since more than 1 read can overlap a cytosine), so this seems to contradict the description. Moreover, if I just take the description at face value as "epialleles with similar methylation properties that is defined by thresholding", it's not clear what this represents biologically. It seems to intend to measure variability of methylation.
A: We understand that VEF is a newly introduced metric and that it should be explained in detail. Instead of providing more text, we have now included a panel B in Figure 1, where we graphically explain how VEF values are calculated at the level of individual bases. We believe that this figure panel, with its accompanying legend, will make the underlying calculations of VEF more easily accessible for the reader. In addition, the extensive online documentation to which we point our users (at https://bbcg.github.io/epialleleR/articles/epialleleR.html or https://bioconductor.org/packages/devel/bioc/vignettes/epialleleR/inst/doc/epialleleR.ht ml) provides further clarifications and scenarios for specific analyses.
Revision: Thank you for providing the graphical description. It is now much clearer what the parameters in the VEF calculation are describing. However, related to previous concerns, there is no justification for why each of the example patterns presented in newly added Figure 1B can be attributed based on a single read to: sequencing artifacts, 'true biologically relevant epimutations', incomplete bisulfite conversion, or 'scattered methylation'.
Original submission: It's not clear how VEF metrics compare to established measures of methylation variability and/or allelic variation of methylation (e.g. Variably Methylated Region identification, methHaplo, methtuple, DAMEfinder). There are alternatives discussed in terms of entropy and discordant reads, but they are not compared in the sensitivity analysis study of WBC.
A: Variably methylated regions (VMR), by definition, are the regions showing variable methylation levels between samples. Such regions are not what epialleleR is designed to quantify. Unlike VMR tools, epialleleR outputs per-base value (VEF) that can significantly boost sensitivity of aberrant or differentially methylated region detection tools for the detection of mosaic epimutations (epimutations present in 1 out of >100 cells). This is presented in the sensitivity analyses that employ the best to our knowledge DMR analysis tool (DMRcate). We agree with the Reviewer that it is always a good practice to compare to established tools. However, the tools suggested by Reviewer (methHaplo, methtuple, DAMEfinder) actually have different purposes than epialleleR. They are used to assemble methylation haplotypes (methHaplo), detect changes in allele-specific methylation between two groups of samples (DAMEfinder), or count m-tuples in reads (methtuple). While they use BAM files as an input, none of them is able to output a metric which is directly comparable to VEF (per-CpG methylation level) or its purpose (to count concordant hypermethylation events, i.e., epimutations). For example, methHaplo specifically excludes CpGs with low methylation level (beta < 0.1) as they are not informative for its purpose. In contrast, epialleleR is designed to detect and quantify rare epimutations, can extract all

epialleles and can use optional VCF data to correlate SNVs with methylation - this was used by us to discover prenatal nature of BRCA1 epimutations that lead to cancer in adulthood. Besides, epialleleR can itself call methylation and use BAM files produced by virtually any aligners (which is not the case for tools mentioned above). The other metrics (entropy, PDR, etc) are not per-base metrics and cannot be used for differential/aberrant methylation analysis. As such, we feel it would leave the readers with a misleading impression about the tools and their purpose if we tried to present any comparison, and we have therefore not done this in the sensitivity analysis section.
Revision: When the authors are addressing my first major concern, which is regarding the clarity of presentation of the purpose of their method, some of the discussion provided in the authors' response to this point may be useful to include in a revised version.
Original submission: A major limitation of the interpretation of so-called epialleles is the inability to distinguish allelic variation from cell type heterogeneity. This is acknowledged very briefly and very late in the manuscript. This needs to be stated up front. In addition, a discussion of potential remedies (if any) would be helpful.
A: We agree that cell type heterogeneity is a factor that must be taken into consideration in methylation analyses in general. But, while cell type heterogeneity can explain some of the methylation differences in other studies, the phenomenon we have described here does not depend on it. Only 1 CpG in this study and none of CpGs analysed in LÃ¸nning et al., 2022 have significantly different methylation levels between blood cell types. Regarding other regions, it is important to note that our method, epialleleR, is not itself biased by cell type heterogeneity because it is executed upstream of the data interpretation. epialleleR reports frequencies of individual epialleles or their groups (by methylation), while it is the downstream analysis (comparison of samples or their groups) which should account for any confounding factor, including, but not limited to, cell type heterogeneity. Given the possibility to perform allele-specific methylation calls with epialleleR, we actually believe it will be a useful addition to tools that may be used to address cell type heterogeneity, since methylation on different alleles in different tissues will indicate different methylation events to have occurred in different tissues of the same individual.
Revision: While the authors assert that the limited set of CpGs associated with one particular gene (BRCA1) do not widely exhibit differential methylation levels by cell type, they do not propose that their tool is aimed at analysing only 37 CpGs. Instead, they argue the tool is applicable on methylation sequencing data with base-level resolution - including whole genome bisulfite data. As such, my original concern remains regarding the confounding factor of cell type heterogeneity: "This is acknowledged very briefly and very late in the manuscript. This needs to be stated up front. In addition, a discussion of potential remedies (if any) would be helpful."
Original submission: The simulated data are very unrealistic, which precludes interpretation of results. It seems that all CpGs were simulated with the same methylation level of 50%, and all other cyosines at 0.25% methylation levels. More importantly, introducing random sequencing errors is unrealistic, as per base sequence quality is known to be generally lower in read 2 compared to read 1. Further, there's a known increase in read 2 of M bias in bisulfite sequencing. With a more realistic error generating process, I expect choosing base with highest quality to be effectively the same as trimming read 2, meaning the results presented here are an artifact of simulation choices.
A: The purpose of the simulated data is to have a known level of methylation of all cytosines, so that the reported values can be compared to the expected ones. While the ground truth was chosen to be

exactly 50% methylation in CG context and approximately 0.25% in CHG and CHH ones, this does not undermine the results of the comparison. We believe, the values applied are not relevant per se; they could have been 5% or 95% or anything else. The tools are still compared based on the lowest difference of observed (reported) values to the expected ones. The Sherman bisulfite read simulator used by us here is a state-of-the-art tool, which uses the most realistic simulation strategy available. The errors are not entirely random but introduced according to exponential decay model (derived from real data). Simulated per-base quality scores follow the same curve as well. As pointed out by the Reviewer, it is indeed known that read 2 contains more base substitutions than read 1. However, the number of sequencing errors depends strongly on a sequence context and read length, therefore errors are present in both read 1 and read 2, and are more abundant at their ends. It is a quite common situation that a lower-quality 3' end of read 1 overlaps with higher-quality region of read 2 (either middle or its 5'). A simple trimming of a higher-quality part of read 2 will then result in lower-quality bases retained. This is why the base quality-driven merging will naturally result in fewer errors and we therefore consider it to be favourable. The subtle increase in reporting accuracy is not very valuable on its own, but together with the vast speed improvement it allows us to recommend our method for virtually every analysis of methylation sequencing BAM files when methylation needs to be called at individual cytosine residues.
Revision: Thank you for the additional information regarding the exponential decay model of errors - this information would be helpful to add to the manuscript, as not all readers will be intimately familiar with the arguments of this particular simulator software.
Original submission: Owing to the major concerns listed thus far, I am not able to fully review the "Sensitivity analyses" section at this time.
A: We hope that the revisions in the manuscript text and the comments made here in our response letter, are clarifying and that all parts of the manuscript are now easily accessible for the reader.
Revision: Many of the original concerns have not been fully addressed. However, at this time I'd like to point out that the sensitivity analysis section seems to be focused on analyses that only include a handful of specific regions of interest. Are these required for detection of 'epialleles'? If not, an example / evaluation that does not require a priori specification of these regions (e.g. is more of a genome-wide investigation) would be helpful, as well as a discussion of region-based vs individual cytosine-based analyses as they pertain to epialleleR.
Minor:
Original submission: epialleleR is marked in Table 1 as not requiring a reference genome. But isn't it needed to obtain the bam file? If not, then how was the bam file generated for use with epiallelR? It's not a fair comparison to mark Bismark as needing a reference genome (to carry out the bam file generation step), as far as I'm aware this isn't used in the methylation extractor step in Bismark (the input is a BAM/SAM file).
A: Bismark methylation extractor indeed requires reference genome in addition to BAM input. For the methylation reporting, epialleleR does not require reference genome (similar to methylKit, but unlike methylation reporting by DRAGEN or Bismark).
Revision: Can you please point me to a reference that shows you need a reference genome to run Bismark's methylation extractor? The documentation for this tool does not mention anything about a reference genome: https://felixkrueger.github.io/Bismark/options/methylation_extraction/. As I pointed out before, like epialleleR, it only requires BAM/SAM from an alignment.

Original submission: When the authors state "Concordantly methylated alleles (genomic regions with spatially extended / haplotype-specific methylation)", I think they just mean allele-specific methylation. If not, please clarify. The other terms used (concordant methylation or spatially extended methylation) are nonstandard, and, puzzlingly, not mentioned in the citations provided for this sentence, where I searched for additional context.

A: By concordantly methylated alleles we mean reads with concordant methylation of cytosines (e.g., most of CpGs either methylated or unmethylated). We agree that this was unclear in our original manuscript and have now replaced the sentence with a simple explanation for "Concordantly methylated alleles" in the first sentence of the "Sensitivity analyses" paragraph of the Results section.

Revision: Thank you for the clarification. The revised statement now refers only to alleles with most CpGs methylated and is rather vague. It's not clear why reads with most CpGs unmethylated are not biologically important. What do the authors mean by "biological importance" here? Note that lots of reads will show most CpGs either methylated or unmethylated, so the specific references cited don't seem to support this broad claim.

Original submission: In Figure 2A what region is shown and how was this chosen? It appears the beta values are shown on a log scale, which is a nonstandard choice. M-values would be advisable to check as well.

A: Figure 2A shows all 138 CpGs included in the sensitivity analyses, with their genomic position as a categorical variable along x-axis. We understand that this was unclear and have now included an additional explanation in the figure legend. Data are presented using log scale to illustrate exponential (3-fold) differences between cytosine methylation levels (1e-4 to 1e-2) between all samples. This would not be possible using a linear scale and we have therefore chosen to keep the log scale in the figure. As epialleleR cytosine reports are similar to Bismark cytosine reports in that epialleleR outputs both numbers of methylated ("Ca" with thresholding or "C" without thresholding) and unmethylated ("T") cytosines per genomic position, the package users can utilize those counts in any downstream analyses in the form of a beta value or M-value. Here, we chose to present beta/VEF values only, as sequencing data is compared with methylation array data, for which M-values are not available.

Revision: M-values are certainly applicable to array data (https://doi.org/10.1186/1471-2105-11-587). However, after reviewing a bit about the experimental setup (that this is focused on primarily unmethylated regions), the motivation for using M-values which allow for comparing regions of varying baseline methylation level is not very relevant.

Original submission: In Figure 2D/E, a measure of specificity should be reported as well.

A: The experimental setup allowed us to extract only the total number of real positive regions (P), the number of true positive regions (TP), and the number of false negative regions (FN=P-TP) for every analysis, while the numbers of true negative (TN) or false positive (FP) regions were undefined. Therefore, recall, or true positive rate (TPR=TP/P) was chosen as a sensitivity metric. This is described in Materials and Methods and the TPRs are now presented in the revised manuscript.

Revision: Can the authors please explain what about the experimental setup precludes identification of TN and FP?

Methods

Are the methods appropriate to the aims of the study, are they well described, and are necessary controls included? Choose an item.

Conclusions

Are the conclusions adequately supported by the data shown? Choose an item.

Reporting Standards

Does the manuscript adhere to the journal's guidelines on minimum standards of reporting? Choose an item.

Choose an item.

Statistics

Are you able to assess all statistics in the manuscript, including the appropriateness of statistical tests used? Choose an item.

Quality of Written English

Please indicate the quality of language in the manuscript: Choose an item.

Declaration of Competing Interests

Please complete a declaration of competing interests, considering the following questions:

- Have you in the past five years received reimbursements, fees, funding, or salary from an organisation that may in any way gain or lose financially from the publication of this manuscript, either now or in the future?
- Do you hold any stocks or shares in an organisation that may in any way gain or lose financially from the publication of this manuscript, either now or in the future?
- Do you hold or are you currently applying for any patents relating to the content of the manuscript?
- Have you received reimbursements, fees, funding, or salary from an organization that holds or has applied for patents relating to the content of the manuscript?
- Do you have any other financial competing interests?
- Do you have any non-financial competing interests in relation to this paper?

If you can answer no to all of the above, write 'I declare that I have no competing interests' below. If your reply is yes to any, please give details below.

I have no competing interests

I agree to the open peer review policy of the journal. I understand that my name will be included on my report to the authors and, if the manuscript is accepted for publication, my named report including any attachments I upload will be posted on the website along with the authors' responses. I agree for my report to be made available under an Open Access Creative Commons CC-BY license (http://creativecommons.org/licenses/by/4.0/). I understand that any comments which I do not wish to be included in my named report can be included as confidential comments to the editors, which will not be published.

Choose an item.

To further support our reviewers, we have joined with Publons, where you can gain additional credit to further highlight your hard work (see: https://publons.com/journal/530/gigascience). On publication of this paper, your review will be automatically added to Publons, you can then choose whether or not to claim your Publons credit. I understand this statement.

Yes Choose an item.
